# Exploring the characteristics of a local demand for African wild meat: A focus group study of long-term Ghanaian residents in the Netherlands

**DOI:** 10.1371/journal.pone.0246868

**Published:** 2021-02-16

**Authors:** Sandrella M. Morrison-Lanjouw, Roel A. Coutinho, Kwasi Boahene, Robert Pool

**Affiliations:** 1 University Medical Center Utrecht (UMCU/Julius Center), Utrecht, Netherlands; 2 PharmAccess Foundation, Amsterdam, Netherlands; 3 Utrecht University, Utrecht, Netherlands; 4 Department of Anthropology, University of Amsterdam, Amsterdam, Netherlands; Universidad de La Frontera, CHILE

## Abstract

While there is a growing body of research documenting unregulated African wild meat imports into Europe from the Africa continent, the drivers of this demand are virtually unknown. This study employs focus group discussions and a survey questionnaire to examine the attitudes and practices related to African wild meat consumption in the city of Amsterdam, Netherlands. The Ghanaian community was selected as the object of this study, as it is the largest West African population in the Netherlands and represents an important part of Dutch society. We model our report on a recent US study of the Liberian community of Minneapolis, Minnesota, which allows for the comparison of results between two Western countries. The overall perceived health risk of consuming African wild meat in The Netherlands is low and unlikely to deter consumption. However, local prices for the meat may be prohibitive in some cases. Incentives include health benefits, cultural drivers and a strong preference for the taste of African wild meat over all local meat alternatives. The study calls for further research into the nature of the drivers of demand for African wild meat as well as its public health consequences, in the Netherlands and beyond.

## Introduction

Since the beginning of time people have hunted wild meat for food. Wild game is still coveted today across the world from the global North to the global South, though the species of animals may vary. Throughout West and Central Africa wild meat (also referred to as ‘bushmeat’) is a primary source of protein and micronutrients for a part of the population [1–7]. However, while populations residing in remote rural settings in West and Central Africa may consume this meat out of necessity, it has been observed that there has also been an increase in this type of meat consumption in urban and peri-urban areas where it may be considered a luxury item [8, 9].

Recent studies provide evidence that significant quantities of African wild meat enter Europe each year [10–14]. Chaber [10] estimated that upwards of 270 tonnes of African wild meat may be passing through major European airports annually. It is unclear whether this meat is arriving to meet a local demand or is in transit through Europe to other markets such as the United States, where there is a very large African community [15]. As with most illicit trades, quantifying the African wild meat coming into a country is challenging. This is compounded by the fact that African wild meat, when it is seized at border points of entry, is often lumped together by customs officers, and immediately incinerated pursuant local biohazard protocols. This makes it exceedingly difficult for global health experts, tasked with tracking the movement of pathogens from biosurveillance hotspots, to precisely assess the possible health risks posed by the African wild meat trade.

While an extensive literature exists concerning the drivers of a local demand for African wild meat in West and Central Africa, there is a relative paucity of academic research into the demand for this meat within émigré communities [16–19]. Yet, a possible motivation may be a desire to stay connected with one’s cultural heritage. After all, food is at the heart of many cultural practices, beliefs about health and, in some cases, spiritual beliefs [7]. Consequently, immigrants from countries where African wild meat was traditionally consumed may continue to seek it out long after leaving their country for a new home [20]. Furthermore, studies by Wilkie et al. [21] and [2] demonstrate that even in the case of internal migrants within African countries such as Gabon, wild meat is used to stay connected to rural living after moving to an urban life. This could very easily be extrapolated to Africans that move abroad.

The very nature of African wild meat as a product may also be driving demand. One of the most significant findings from an ongoing study by Brashares [22] is that the demand for African wild meat in the West is not driven by poverty; he observes that prices paid for African wild meat are often higher than that of local domestic meat. These results suggest that African wild meat can be regarded as what economists would term a “normal” or even a “luxury” good, in other words where the income elasticity of demand is at least positive and possibly greater than one. Rising incomes may thus be expected to drive increased demand for wild meat with, in the case of luxury goods, demand increasing disproportionately. Brashares [22] suggests that, particularly in expatriate communities, African wild meat is, indeed, considered a luxury good, as volunteers participating in the ongoing Brashares study found that primate meat (which is relatively expensive compared to other wild meat species) made up a greater share of the wild meat sold in overseas markets as opposed to in West- and Central-African markets. As a result, urbanization–and more specifically the rise in income often associated with it–may well be expected to lead to increased demand for wild meat not only in Africa, but wherever the diaspora exists.

Understanding the incentives for, and attitudes towards, the import and consumption of African wild meat is a first step towards identifying the scale and scope of the trade and, thereby, how human, animal, and environmental health may be affected by it. In this paper we seek to gain additional insights into the demand for African wild meat through a qualitative study of attitudes towards African wild meat consumption within the local Ghanaian community in The Netherlands. The Ghanaian community represents the largest West African community in the country, and it continues to grow both in size and wealth. With this study we improve our understanding of the intercontinental supply chain for African wild meat.

## Methods

This study was granted written approval by the Ethics Review Board of the University Medical Center Utrecht (UMCU) pursuant to the rules and regulations created and updated regularly by the Board of UMCU. The criteria about how to carry out studies involving what could be considered a vulnerable population was given considerable attention. The Board decided that there were no issues of compliance. The identities of all members of the three focus groups in addition to the answers provided in the written questionnaires were kept anonymous.

### Design

We report on an exploratory qualitative study of African wild meat consumption among long-term Ghanaian residents in the Netherlands using focus group discussions and a written questionnaire. The study focusses on three key themes: 1) consumption frequency and motivations 2) perceived health risks and 3) acquisition and pricing. Analyzing these three areas offers insights into the perceived health risks associated with, and socio-economic drivers of, African wild meat consumption in the Netherlands. The study is modelled on a recent United States focus group study that explored the local demand for African wild meat within the Liberian community in Minneapolis, Minnesota [23]. As such we employ the same definition of African wild meat as in that study, considering meat from animals such as rodents, hooved animals, carnivores, primates and bats. Note that this includes neither fish nor fowl. Basing our study on this model allows us to make tentative comparisons between the two countries. In addition to the questions included in the Walz [23] study, we included a series of additional questions to further explore the socio-economic and anthropological drivers of a local demand. For a complete overview of the questionnaire used in this study, see S1 Appendix.

A pilot and three focus groups (5–8 participants, each 90 min) were conducted over a period of 6 weeks, in the Fall of 2018. A standard discussion map (S2 Appendix) was used for each session. Information from the pilot is not used in the analysis reported here. A written questionnaire was administered at the conclusion of each focus group discussion. A fourth group was administered a questionnaire, but did not participate in any discussion. This group was selected from contacts of the participants in the third focus group. A couple of questions in the questionnaire were modified after the first focus group, to reflect new topics that were discussed but not reflected in the original questionnaire.

### Setting and participants

The participants were recruited by a Ghanaian leader residing in Amsterdam. He contacted members of his community and asked them to share their thoughts and feelings on the topic of African wild meat and health. Inclusion criteria included: i) minimum age of 18; ii) self-identification as a Ghanaian iii) willingness to discuss African wild meat in a group setting. Each focus group session was conducted in the home of a participant of the focus group. At the beginning of each session participants were informed about the nature and aims of the study and participating members of the study team were introduced. Poole and Boahene were both present for the pilot and the first focus group discussion. Morrison-Lanjouw was present at all sessions.

### Data collection and treatment

All discussions were recorded and transcribed by an independent third party. Two of the authors analyzed each transcript by assigning labels to different parts of the data (open coding) and then identifying any connections between them (axial coding). Numerical data from the answers collected in the questionnaires were stored in Excel and converted into raw percentages, recalculated removing non-answers from the denominator and rounded off to the nearest tenth.

## Results

The results of the study are organized according to the three overarching themes previously described. First, we discuss the consumption frequency of African wild meat and the underlying drivers of this demand. Next, we explore the perceived health implications of African wild meat consumption. Finally, we consider the characteristics of the local African wild meat market in terms of both acquisition and pricing. All numerical data is derived from the aggregation of questionnaire responses. Further context is provided using insights from the three discussion sessions.

Given the small sample size of this study, individual characteristics are likely to be associated with specific results. Accordingly, the demographic characteristics of the sample of participants should be noted. However, given the formulation of questions and the group discussions, it seems likely that individual responses represent the collective practices and behavior of the households to which respondents belong. It is not clear that responses can be interpreted to represent the choices and preferences of individual respondents. A summary of participants’ characteristics is provided in Table 1, below.

**Table 1:** Demographic characteristics of focus group participants.

Category		Number of Participants	Share (%)*
**Gender**	Male	15	54.6
	Female	13	46.4
**Age (years)**	18–29	1	3.6
	30–50	5	17.9
	51–65	17	60.7
	66–80	5	17.9
**Years of residency in the Netherlands**	< 1	2	7.1
	1–4	0	0
	5–10	3	10.7
	11–25	5	17.9
	26–35	14	50
	36–50	4	14.3

*All percentages are rounded to the nearest tenth.

### Consumption frequency and drivers

There were many drivers discussed in the focus sessions and underscored in the questionnaire. The most significant of these was taste. When asked, in the written questionnaire, what participants liked most about the African wild meat they ate, the most common answer was taste (44%). This was followed by its cultural and traditional importance (16%). Others emphasized the medicinal and other health-related qualities associated with African wild meat consumption. Those who did not eat bushmeat either did not answer the question or indicated that they did not eat African wild meat. During discussions participants stated that they prefer the taste of African wild meat to any meat available in the Netherlands.

The smoking process used in the preparation of African wild meat contributes heavily to giving it the sought-after flavor. During discussions there was a range of taste preferences, from pink centered smoked meats to almost burnt (S3 Appendix). The traditional smoking process, which involves flame-singeing of the meat with the fur on, gives the meat a unique flavor which cannot be found in locally available meat. Some types of African wild meat were claimed to be so fragrant that a small amount suffices to flavor an entire meal.

With regards to the preferred species of African wild meat, there was no consensus. Akrantie (also called a grasscutter, *Thryonomys swinderianus)* was discussed most frequently as the preferred species of African wild meat. Monkey meat was also highly sought after. There did not appear to be a preferred species of monkey as participants distinguished only between two categories: *adow* (a “house” or pet monkey) and *okokwo* (a monkey hunted in the wild and eaten). Deer, greater cane rat, porcupine, bush pig, lizard, wild fox, gorilla, squirrel, baboon, bat, wild rabbit, cat and possibly jackals were all included in the list of animals that could be consumed. Some of these species are not known to be present in Ghana, suggesting that the meat may have a different country of origin, although potential misidentification of species cannot be discounted.

Perhaps unsurprisingly, given that African wild meat was described in focus group discussions not only as superior in taste but also in quality compared to locally available meat, participants were unwilling to consider local meat as an alternative. When asked how they felt about substituting local domestic meat for African wild meat in recipes that traditionally use African wild meat, the majority (69%) of participants were not in favor. When asked about substituting European wild meat for African wild meat, the response was even less favorable, with 92% responding negatively. This is not to say that participants do not consume local domestic meat. In fact, the majority (69.5%) of participants consume African wild meat less than twice a year (see Table 2). Indeed, African wild meat is described, in both the written questionnaires and group discussion, as too expensive to form a regular item in the family diet.

**Table 2:** Wild meat consumption frequency.

Frequency of African wild meat consumption (Annually)	Number of Participants	Share (%)*
**No Answer**	5	-
**Never**	3	13
**Less than once**	7	30.4
**1–2 times**	6	26.1
**3–5 times**	3	13
**6–10 times**	0	0
**more than 10 times**	4	17.4

*Percentages are rounded to the nearest tenth. Non-answers are excluded.

In the written questionnaire, the consumption of African wild meat was said by all participants to form part of their culture. This sentiment was echoed during the focus group sessions. Indeed, in all focus groups discussions African wild meat was seen by participants as an important way to stay connected to the continent and their cultural roots (S3 Appendix). Many claimed that it had been a part of their diet since a young age. Some participants expressed a belief that they will continue consuming African wild meat for as long as they live regardless of where they reside (S3 Appendix).

In contrast, some female participants described a certain negative attitude towards eating African wild meat amongst the younger generations. They are perceived to prefer local foods, such as “patat” (French fries) and “krokets” (fried snacks containing meat ragout). As a result, some in the discussion groups believed that the consumption of African wild meat in the Ghanaian community may die out over time. Others disagreed with this assessment arguing that when a child grows-up they will come back to traditional food, possibly to stay connected to their more distant cultural roots. During discussions it was also expressed that it makes a difference *when* African wild meat is introduced into the diet of young people. If a young child is exposed to this meat it is thought that they are likely to acquire a taste for it later in life. Given these various theories, it is difficult to predict whether future generations’ demand for African wild meat will remain strong and, if so, whether it will be driven by taste, cultural practices, or a combination of both.

### Perceived health risks

Participants were generally skeptical of the alleged health risks associated with African wild meat consumption, arguing that if African wild meat consumption was as dangerous as some make it out to be, then the number of deaths in Ghana would be higher. Moreover, they pointed out, outbreaks would not be localized; they would be more widespread. As a result, the majority (60%) of participants expressed no concerns over the health risks of African wild meat consumption. During discussions regarding the existence of Ebola in Ghana, there were divergent points of view. The general perception was that the Ebola crisis in West Africa led to a temporarily fall in the consumption of African wild meat in Ghana. The most serious risk associated with the consumption of contaminated African wild meat, identified by the members of the focus groups, was diarrhea. Even then, however, they argued that the source of the bacteria is unclear–water, common food poisoning–and did not consider it to be a serious threat.

This confidence is striking, particularly given that, during discussions, participants frequently admitted that the origins of the African wild meat they consumed were unknown. During the discussion sessions, participants proposed that much of it was likely sourced from Ghanaian markets (also referred to as “*abbatoirs”)* where both raw and smoked meat can be bought. At least two focus group respondents, however, raised the possibility that some of the African wild meat found in these markets may have been caught in neighboring countries and sold in Ghana, as a result of the forest degradation occurring in that country. Participants described common methods used to prepare this meat including singeing or using hot water to remove fur, smoking and dry roasting. While raw meat can be purchased in the Netherlands, it was common belief amongst participants that the drier the meat, the more certain its quality, since it is less likely to mold. During group discussions about wild meat in Ghana there was no mention of any sanitary or veterinary quality checks.

Despite the uncertainty over its exact origins, participants commonly believed African wild meat to be of superior quality to the meat sold in Dutch local grocery stores. Group discussion participants indicated a general concern about the chemicals and preservatives used in the local meat industry in the Netherlands and described African wild meat as a more organic alternative. In addition, wild animal meat from Africa is considered to be leaner, more natural, and healthier than any local meat alternatives.

While concern over the health risks associated with African wild meat was generally low, participants did discuss approaches and precautions taken to minimize said risks. Of those who answered the written question regarding the best way to kill pathogens in African wild meat, 40% believed that boiling the meat is most effective, while 25% preferred smoking the meat. The smoking method was argued to be an effective way of preventing weevils and other insects from entering the meat, while also allowing the meat to be stored in the oven or freezer for extended periods of time. There was a consensus that one way to ascertain if meat was properly smoked was to check for the presence of parasites and fungus or mold.

In general, participants expressed confidence in the experience of their forefathers who have hunted, butchered, and prepared meat for centuries. Traditional hunters are believed to be aware of which animals are reservoir hosts for certain diseases due to communal knowledge accumulated over the generations. During group discussions it was mentioned that, as a result, it is not uncommon in parts of Ghana for hunters to bring home animals found dead in the forest to butcher and consume, without risking disease.

As consequence of this faith in tradition, the focus group participants expressed their concern about the fact that hunting methods in Ghana appear to be changing. They felt that traditional subsistence hunting methods were increasingly being replaced by unconventional commercial methods of hunting that may make use of harmful chemicals and poisons. These commercial hunters, they added, lack important traditional knowledge about what different animals eat, and what kind of leaf, herb or bark could remedy adverse effects if the meat were to be contaminated. As result, the reasons people get sick from wild meat may no longer be the same, due to the more widespread use of poison and chemical preservatives.

### Acquisition and pricing

With regards to the price of African wild meat on the local market, there was little consensus. Some participants described African wild meat as so costly that it is sold by the gram. Others, during one of the discussion sessions, quoted a price of 15–20 euros for a small piece of meat. On the written questionnaire, several participants indicated prices upwards of 25 euros per kilo. While this is within the broad range of prices observed for local wild meats, it is well above the price charged for most common domestically-farmed meats. The broad pricing spectrum of African wild meat can likely be attributed at least in part to differences between participants with regards to the species being considered, as these were not specified on the questionnaire. It was very likely that respondents referred to only the species purchased most recently. Alternatively, the variance may simply reflect a lack of certainty amongst the participants. This would be understandable considering the vast majority (63%) indicated that they acquire their African wild meat from visiting friends and family, as opposed to purchasing it from local providers (see Table 3).

**Table 3:** Primary African wild meat acquisition methods.

Most common source of African wild meat	Number of Participants	Share (%)*
**No answer**	4	-
**Through friends/family**	15	62.5
**Mail/Courier**	0	0
**Local Butcher**	5	20.8
**Local Restaurant**	0	0
**I do not buy wild meat in the Netherlands**	2	8.3
**Bring it myself**	2	8.3

*Percentages are rounded to the nearest tenth. Non-answers are excluded.

It is apparent, however, that African wild meat is relatively expensive. In both their responses to the written questionnaire and the group discussions, participants emphasized that African wild meat is expensive in the Netherlands relative to Ghana. Several participants even described how, as a result of these high prices, it is sometimes necessary to carefully balance the purchase of African wild meat within the household budget in order to ensure that the bills can be paid each month.

During group discussion participants expressed a belief that these elevated prices are a result of the internalization of the costs and risks associated with transporting African wild meat internationally. The participants did not clarify whether there are price differences associated with transporting African wild meat via air mail, shipment by sea or some other transportation method. They did suggest, however, that there exists a commercial trade in African wild meat beyond that which is brought over from Ghana via passenger air travel. Seasonal availability was also said to affect the flow of African wild meat into the Netherlands, with the rainy season (a period of relative bounty) stretching roughly from April to October and the closed hunting season spanning from August to December.

Participants of group discussions noted that it is a common misconception that African wild meat is consumed only by the poor. The reality is that both the rich and the poor consume, and therefore compete for, the same meat in Ghana. In the Netherlands, African wild meat is often very costly and as a result, it has taken on a role as a status symbol. Although there was no consensus as to its exact form, focus group participants described a hierarchy of species in which a higher price is accompanied by an increased sense of pride. Some argued, for example, that the purchase of an *akrantie* to share with family and friends brings more pride to an individual than serving a dish using the meat of other small rodents or mammals. These participants strongly felt that, given the means, one can afford to be specific and acquire the meat of only certain desirable species, such as antelope or *akrantie*.

## Discussion

This study explores dynamics of a local demand for African wild meat among long-term Ghanaian migrants in the Netherlands. As indicated in S3 Appendix, there is significant overlap between the findings of this study and those from an earlier study carried out in the US which explored African wild meat consumption in the Liberian community of Minneapolis, MN [23]. These include the cultural drivers of a local demand, taste preferences, health risk perceptions and methods of acquiring the meat in both the US and The Netherlands. This lends credence to the notion that similar drivers of demand are in play across different African expatriate communities.

In both study populations taste was considered one of the main reasons that African wild meat is consumed. One method of preparing the meat involves flame-singeing the meat, with the fur on, which gives it a smoky flavor that is highly sought after by Central and West African people. Meat prepared this way has been referred to as “smokies” by butchers who use this method in Europe but it is strictly prohibited by EU law, as health authorities continue to investigate the related health risks [24].

The present study further explores the question of whether local meat can substitute for African wild meat in traditional recipes. It is striking that the majority of participants felt negatively about this alternative, which specifically included local *wild* meat. This suggests that it is not only the “wild” aspect that drives the demand for African wild meat, but other factors, such as the fact that it is specifically from Africa (i.e. from “home”). This is consistent with the US study that describes participants as seeking out this meat to feel connected to their cultural roots–their homeland. Given that many preferred species of animals are not found in Europe, and some desired methods of smoking the meat are not common practice, it is unlikely that African wild meat can be entirely replaced with local alternatives.

Health perceptions about the consumption of African wild meat are consistent across both the Dutch and US studies. In both study populations boiling African wild meat for a long period of time was believed to be an effective way to kill all dangerous pathogens. Both groups were skeptical of potential zoonoses, pointing to the generally incident-free experience of their ancestors. This perception may be problematic, because there is extensive literature describing diseases associated with the hunting, butchering and consumption of African wild meat that includes retroviruses such as Simian Foamy Virus (SMF), HIV I and II, Monkeypox, Ebola, and Herpesviruses [15, 25–29]. Furthermore there is still a gap in knowledge regarding the full range of pathogens in commonly consumed African wild meat species, and the identification of reservoir hosts is ongoing. Despite this, the individual lifetime risk of contracting a deadly zoonotic disease is near zero. Should a disease spillover occur, however, the public health consequences could be disastrous.

Both studies report that African wild meat is believed to be more “healthy” and “natural” than locally available meat because it is free of chemical additives, pesticides and it is a leaner meat. Such sentiments resonate even within native-born communities, as witnessed by the rapid growth of the organic-food movement in the U.S. and the Netherlands. At the same time, roughly two thirds of subjects reported eating wild meat no more than twice a year. As many as two fifths of respondents had either not eaten wild meat in the last year or had not consumed African wild meat at all. Based on these responses it remains a bit unclear exactly how, and to what extent, wild-meat consumption is thought to contribute to better health.

Further research is needed to clarify whether African wild meat is a “normal” good for which consumption would be expected to rise as incomes grow, or even a luxury good which may be considered a status symbol. The status value of this meat is inextricably tied to the high prices that people are willing to pay. African wild meat is difficult to acquire and the high purchase prices reflect this dynamic. However, this should not be confused with the intrinsic cultural symbolism of sharing meat from their homeland. These considerations lend support to the idea that while African wild meat may be considered a luxury item and at times may even be prohibitively expensive in the Netherlands, other considerations may reduce the sensitivity of demand, within the Ghanaian community, to price signals. This implies that there may be more regular consumption of African wild meat than can be inferred from the responses to the questionnaire.

While culture, taste preferences, health considerations and an increase in disposable income may all be driving the local demand for African wild meat, there is also some suggestion that the younger generations within the Ghanaian community are less predisposed. As can be seen in Table 1 it is noteworthy that most respondents in the study are over the age of 50 (80%) and have been residing in the Netherlands for over 25 years (64%). The parameters governing their demand are likely different from those of the younger generation and of recent arrivals.

More research is needed to explore these multigenerational issues in a larger and more varied sample of Africans living in the Netherlands, and beyond. Such research would provide insight into the future of African wild meat demand, as well as help determine the drivers most likely to persist across generations as direct links to the African continent fade. Research should also explore the possibility that demand for African wild meat derives from non-African residents in the Netherlands. Anecdotes exist that also point to an interest in wild mean consumption within other communities.

There is an increasing awareness of the transboundary health implications attached to the movement of animal products on a global scale but many gaps in knowledge exist regarding both the demand and supply sides of the industry. For example, it is not widely known what species are most commonly implicated in the wild-meat trade. Scrutiny of the different species of African wild meat consumed in the Netherlands reveals a wide range of animals including rodents, bats, and primates. This is useful information as it relates to current research in the identification of reservoir hosts, notably in light of the fact that it is estimated that up to 75% of emerging infectious diseases are zoonotic in nature [30, 31].

Yet another avenue for further research is to study the health profiles of consumers of African wild meat. It is not likely that local primary physicians are including questions about African wild meat consumption to their West and Central African patients, given the prevailing perception that no one is eating it. In this context it would be important also to differentiate between those risks posed by the emerging infectious diseases versus non-communicable diseases associated with African wild meat consumption.

The hunting methods employed in supply countries may pose another health risk. Study participants believed that traditional, small-scale hunting methods are giving way to large-scale commercial hunting of wild meat and that this has negative repercussions for the quality of the meat. Indeed, this challenges African wild meat’s perception as a healthy, more natural alternative to local meat. While participants in our study distinguished between traditional hunting methods and a new method of hunting that makes use of poison, this is perhaps too simplistic a distinction given that poisons have long been an important part of the hunting process. Nevertheless, the unregulated use of unidentified poisons on animals destined for human consumption should be a major concern for public health authorities. The two chemicals that were mentioned most frequently in this regard were DDT (*Dichlorodiphenyltrichloroethane*, an insecticide) and formaldehyde, though these claims could not be substantiated. It is likely that “DDT” is used as a generic term for a range of unidentified poisons, as DDT would not kill large animals.

In both the Dutch and US studies African wild meat is brought into the country through airports primarily via passenger air travel. In the Dutch study it is described as being brought into the country by friends who go back and forth to visit Ghana. Also mentioned is the possibility of placing an order with a local butcher and subsequently picking it up at the cargo area of Schiphol airport. This suggests the presence of an organized trade structure. Sea ports were further mentioned as a way African wild meat has been imported into Europe, but no further details were provided. When purchasing African wild meat in the Netherlands participants acknowledge that they cannot determine whether the wild meat they consume originates from Ghana, outside of Ghana, or even outside Africa. It cannot be ruled out that the meat was sourced from one of Ghana’s neighboring countries, areas that may represent emerging zoonotic disease hotspots. This may represent another potential biosurveillance blind spot for Dutch authorities with serious public health consequences.

### Limitations and future recommendations

This was a qualitative, exploratory study. The small sample size, age of the participants (most were aged between 51–65), and the fact that most were from the same region in Ghana and have been living in the Netherlands for an extended time, limits the degree to which findings can be generalized. African wild meat cannot be legally brought into the Netherlands, and this may have influenced the nature of the discussions, though we note the enthusiasm with which the participants discussed the topic. The discussions were carried out prior to the completion of the written questionnaire. This could have resulted in answers that were influenced by other participants during the discussion period within each group. Future studies examining the economic dimensions of the African wild meat trade should take pains to establish price points for individual species of meat. A multigenerational study would also be valuable in improving our understandi

## Concluding remark

This exploratory study of the consumption habits and attitudes of members of the Ghanaian community in the Netherlands has been valuable in contributing to our understanding of some of the social and cultural factors that drive a local demand for African wild meat. It cannot not be ascertained whether the Ghanaian community, residing in the Netherlands, represents a lower or higher demand for African wild meat compared to other local African communities. Unregulated African meat imports do not undergo any veterinary or food quality assessment before entering the Netherlands. Therefore, a new research agenda focused on quantifying and evaluating the quality of unregulated African wild meat imports is warranted. Based on what is currently known the lifetime risk of any individual contracting a zoonotic disease from wild meat is tiny. However, should there be a disease spillover to even just one person, this could lead to an outbreak of great consequence as shown by the recent Ebola outbreak in West-Africa.

## Supporting information

S1 Appendix Survey questionnaire. (DOCX)

S2 Appendix Focus group discussion diagram. (DOCX)

S3 Appendix Themes and quotes from two studies. (DOCX)

S4 Appendix Focus group questionnaire results. (DOCX)

S1 File Africa focus group: Session 1. (DOCX)

S2 File Africa focus group: Session 2. (DOCX)

S3 File Africa focus group 3. (DOCX)

S4 File Tabulated results—African focus group data compilation 2018–2019. (DOCX)

S1 Fig (JPG)
