# Supplementary material for: Exploring the characteristics of a local demand for African wild meat: A focus group study of long-term Ghanaian residents in the Netherlands
Source: PLoS One. 2021 Feb 16;16(2):e0246868. doi: 10.1371/journal.pone.0246868 (PMC7886224; doi:10.1371/journal.pone.0246868)
Supplement: S1 Appendix — (DOCX) [file pone.0246868.s001.docx]

S1 Appendix

Food and Culture Survey: African Wild Meat Consumption in the Netherlands

1. What is your age?
2. How long you have lived in the Netherlands
3. What is your country of origin?
4. How often do you travel to your country of origin every year?

- Less than once a year
- 1-2 times a year
- More than 2 times a year

1. What is the name of your favorite traditional recipe that includes African wild meat?
2. How do you feel about substituting local domestic meat instead of African wild meat for this recipe?

- Negative
- Positive

1. Do you have any concerns regarding the health risks that may be associated with wild meat originating from Africa?
   - 1. Yes
     2. No
2. What do you like about African wild meat?
   - 1. Taste
     2. Tradition and Culture
     3. Community Building
     4. Religion
     5. Health Benefits
     6. Other… explain
3. What do you dislike about African wild meat?
4. How often do you consume African wild meat?

- Never
- 0 times a year
- 1-2 times a year
- 3-5 times a year
- 5-10 times a year
- 10+ times a year

1. Are there particular times of the year or special occasions during which African wild meat is consumed here in the Netherlands?
2. If you buy it, how do you acquire bushmeat in the Netherlands?

- Through friends
- Mail/Courier
- Local Butcher
- Local restaurant
- Other… explain:

1. Is it more or less difficult to buy rare kinds of African wild meat in the Netherlands than in West Africa?
   - - More difficult
     - Less difficult
     - Equally as difficult
2. Is African wild meat more expensive in the Netherlands?
   - 1. 1. Yes

2. No

- - 1. If you answered yes, give an example of price per kilo per animal

1. How do you or members of your community like for the African wild meat to be prepared?
   - - Boiled
     - Smoked
     - Grilled
     - Other … Explain
