# Supplementary figures and images for: Exploring the characteristics of a local demand for African wild meat: A focus group study of long-term Ghanaian residents in the Netherlands
Source: PLoS One. 2021 Feb 16;16(2):e0246868. doi: 10.1371/journal.pone.0246868 (PMC7886224; doi:10.1371/journal.pone.0246868)

**S2 Appendix**

**Focus Group Discussion Diagram**

**
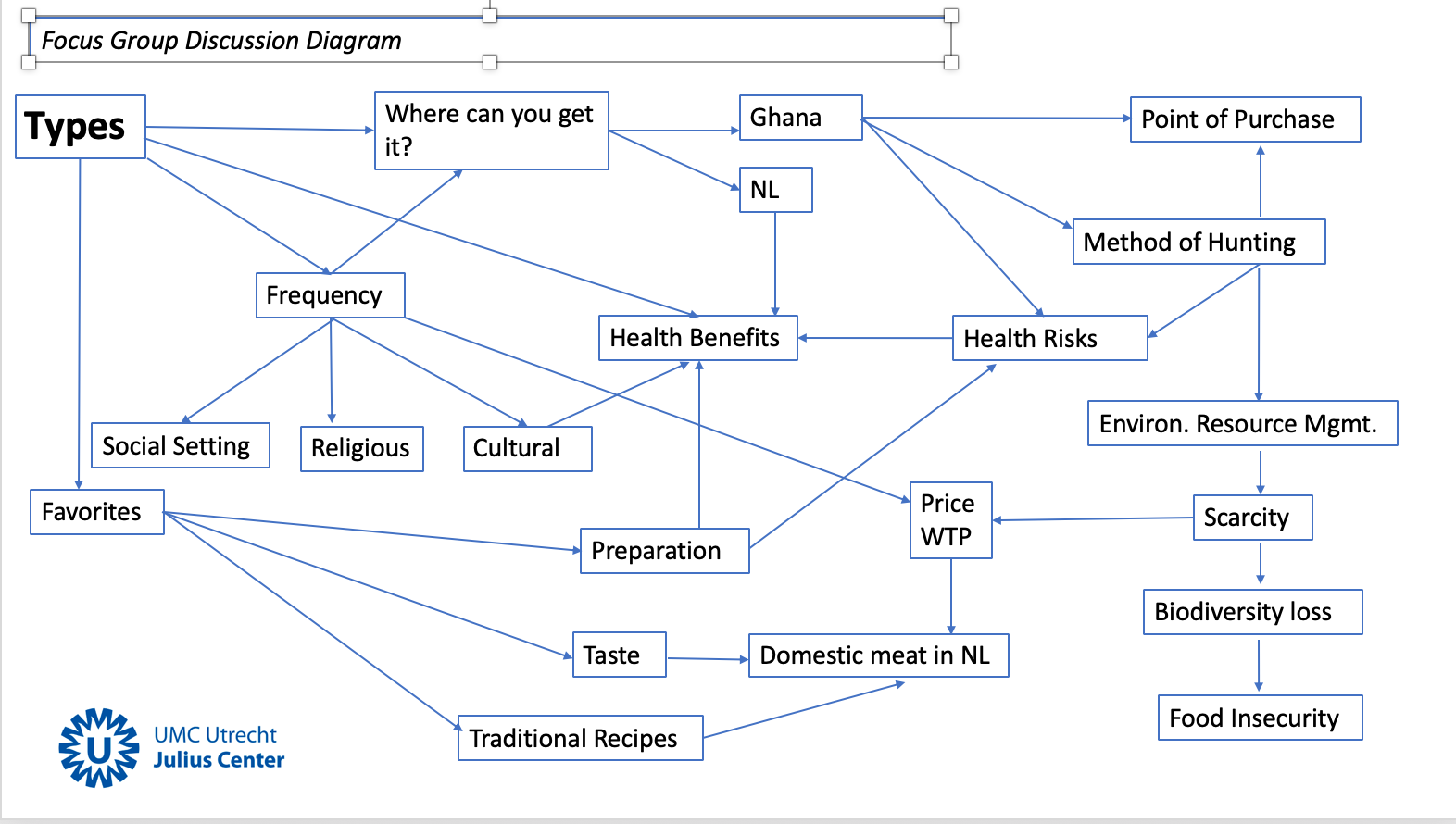
**

**Appendix 4**

Supplement: S2 Appendix — (DOCX) [file pone.0246868.s002.docx]

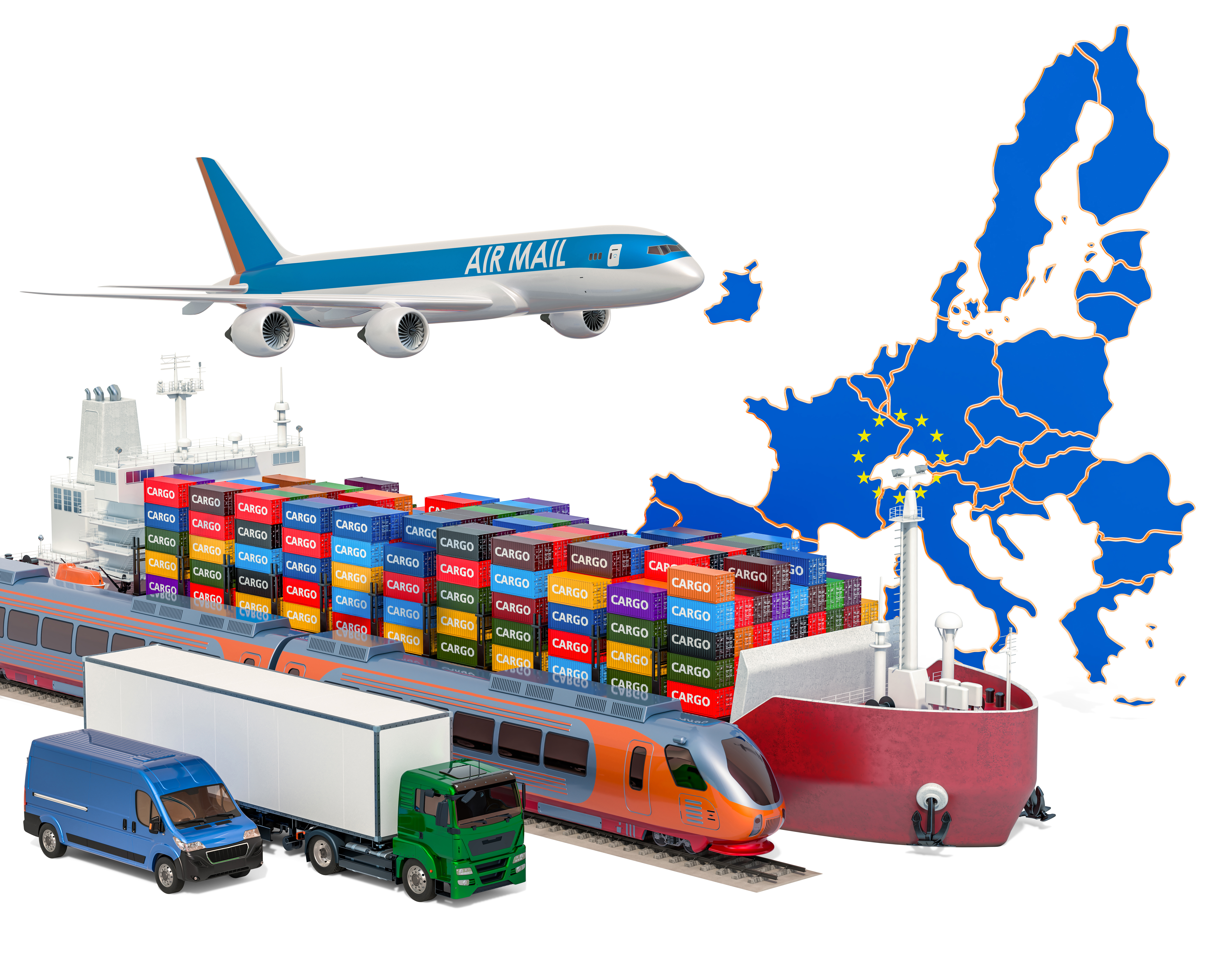

Supplement: S1 Fig — (JPG) [file pone.0246868.s009.jpg]
