# Supplementary material for: Exploring the characteristics of a local demand for African wild meat: A focus group study of long-term Ghanaian residents in the Netherlands
Source: PLoS One. 2021 Feb 16;16(2):e0246868. doi: 10.1371/journal.pone.0246868 (PMC7886224; doi:10.1371/journal.pone.0246868)
Supplement: S3 Appendix — (DOCX) [file pone.0246868.s003.docx]

**S3 Appendix: Themes and quotes from two studies**

|  | **Nostalgia/Cultural Drivers** | **Health Considerations** | **Food Preparation** | **Acquiring African wild meat locally** |
| --- | --- | --- | --- | --- |
| **US Study**  **(Walz et al. 2017)** | “So it goes back to a cultural thing, like she said. The taste and that which you are used to. I mean it’s how you were brought up, and all that stuff. It’s just something like you go away to school and you just miss your mom’s cooking. So that’s just what it is.”  Taste is a driver both in wild meat preferences and method of preparing the meat (ie. smoking process) | Bushmeat is desirable because it is natural.  Wild taste preference.  Consuming meat has special health properties.  “I don’t believe monkey or bats is carrying this virus”.  If there is a risk to eating African wild meat it is still worth it.  Food preparation and sanitation are key to preventing disease or illness when eating African wild meat | “I don’t care how the virus or bacteria is, when you put it on the fire it will not survive a minute.”  ”When we start talking about Ebola, well Ebola did not come from bushmeat, but Ebola may have been on the meat, but when you put it on the fire I don’t think that the Ebola virus could survive”.  African wild meat could carry diseases, but risks are mitigated by cooking. | “You used to be able to bring it in airline luggage, but not anymore.”  “We don’t eat African wild meat because it is not available in MN.”  You can bring African wild meat into the US on a plane in airline baggage.  Postal service used to send African wild meat to the US.  African wild meat sold at some local U.S. stores.  Experiences purchasing bushmeat to bring back to the US.  Acquiring meat from a friend or relative that has traveled back from Liberia |
| **Ghanaian Focus Group Responses** | “We don’t want to eat food from Europe. We want to do our own thing.”    “We have been eating this from our infancy so it is part of our culture.”  “We love eating it, it’s part of our food wherever we go, so long as we are on this earth, we eat it everywhere we go.”  A range of taste preferences exist from smoked with a pink center to almost burnt (which has a more bitter taste).  “It (African wild meat) reminds you of home.”  “If anybody will buy akrantie here being 10 grams or one kilo, it is pure prestige.” | Chemicals used to hunt and preserve African wild meat are a concern.  “It is natural (African wild meat).”    Consuming African wild meat has unique health properties.  Disbelief that bats and monkeys may carry potentially harmful diseases.  “We are not thinking about germs.”  Meat found on the forest floor is not considered dangerous if prepared using traditional methods of cooking. | “About 100 degrees, you will kill any bacteria except the stubborn ones, maybe not Ebola … you put it in the oven and make sure its above 100 degrees.”  Smoked African wild meat can be kept for up to 6 months.  “So when you have dry meat transported from there to here, after a week or two if shouldn’t affect the taste.”  The quality of African wild meat available for purchase in the Netherlands is variable. | “Some animals are more available at some times of the year.”  “No, not only importing it. We smuggle it (into the Netherlands).”  “Yes, it goes very fast for people to place orders. “Do you have it?” Ok, write down your number. This is the quantity.”  Customers are called when the meat has arrived and told they can go directly to the airport to pick it up where items from the “cargo section come out”.  African wild meat can be purchased at local stores and restaurants. |
