# Supplementary material for: Exploring the characteristics of a local demand for African wild meat: A focus group study of long-term Ghanaian residents in the Netherlands
Source: PLoS One. 2021 Feb 16;16(2):e0246868. doi: 10.1371/journal.pone.0246868 (PMC7886224; doi:10.1371/journal.pone.0246868)
Supplement: S4 Appendix — (DOCX) [file pone.0246868.s004.docx]

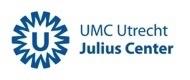


**S4 Appendix**

**Focus Group Questionnaire Results**

Group 1: September 22, 2018 (**8 respondents**)

Group 2: September 29, 2018 (**7 respondents**)

Group 3: October 6, 2018.

(**5 respondents**)

Group 4: October 7, 208.

(**8 respondents**)

**Total = 28 Respondents**

**What is your age?**

**GROUP 1**

(P1):58 Male

(P2): 61 Female

(P3):45 Female

(P4):63 Female

(P5):57 Male

(P6):58 Male

(P7):63 Male

(P8):63 Male

**GROUP 2**

(P1):54 Male

(P2):60 Female

(P3):52 Female

(P4):50 Female

(P5):67 Male

(P6):46 Female

(P7):66 Male

**GROUP #3**

(P1): 70 Male

(P2):42 Female

(P3):55 Male

(P4):44 Male

(P5):56 Male

**GROUP #4**

(P1):22 Female

(P2):67 Male

(P3):63 Female

(P4):53 Male

(P5):57 Female

(P6):48 Female

(P7):52 Female

(P8):68 Male

1. **How long you have lived in the Netherlands?**

**GROUP 1**

(P1): 20 yrs.

(P2): 30 yrs.

(P3): 9 months

(P4): < 6 months

(P5): 31 yrs.

(P6) 28yrs

P7) 27yrs

(P8) 38yrs

**GROUP 2:**

(P1): 25 years

(P2): 36 years

(P3): 30 years

(P4): 30 years

(P5): 29 years

(P6): 20 years

(P7): 38 years

**GROUP 3:**

(P1): 39 years

(P2): 32 years

(P3): 32 years

(P4): 10 years

(P5): 31 years

**GROUP 4**

(P1): 7 years

P(2): 31 years

(P3): 33 years

(P4): 20 years

(P5): 10 years

(P6): 15 years

(P7): 29 years

(P8): 30 years

1. **What is your country of origin?**

**Group 1** – Ghana - All participants

**Group 2** – Ghana - All participants

**Group 3:** Ghana – All participants

**Group 4:** Ghana – All participants

1. **How often do you travel to your country of origin every year?**

- Less than once a year
- 1-2 times a year
- More than 2 times a year

**GROUP 1**

(P2)(P4)(P5)(P6)(P7): 1-2 times a year

(P8): Less than once a year

(P1)(P3):More than 2 times a year

**GROUP 2**

(P2)(P7):1-2 times a year

(P1)(P3)(P4)(P5)(P6): Less than once a year-

More than 2 times a year - 0

**Group 3**

(P4)(P5):1-2 times a year

(P2)(P3): Less than once a year

(P1): More than 2 times a year

**GROUP 4**

P(1)(P2)(P6)(P7): 1-2 times a year

(P3)(P4)(P8): Less than once a year

(P5): More than 2 times a year

1. **What is the name of your favorite traditional recipe that includes wild meat?**

**GROUP 1**

***The following section are all free hand responses**

(P1):Don’t eat meat

(P2):Don’t eat meat

(P3):Light soup with grasscutter

(P4):Grasscutter

(P5):None

(P6):Fufu with Antelope

(P7):Fufu with wild meat

(P8):No answer

**GROUP 2**

(P1): Fufu and Palm nut soup

(P2): No answer

(P3): African wild meat soup

(P4): Fufu and soup

(P5): Soup with grasscutter

(P6): Fufu and soup

(P7): Fufu and Hwee nkwae

**GROUP 3**

(P1): Light soup

(P2) Fufu and soup

(P3)- Remix soup of two or more AWM types

(P4)- Light soup

(P5): Fufu Soup

**GROUP 4**

(P1): Palm nut Soup

(P2): Fufu and goat meat

(P3): Rice with fish

(P4): No answer

(P5): I do not eat meat

(P6): I do not like wild meat so I have no favorite traditional recipe

(P7): Soup

(P8): Do not know because I do not eat wild animals

**What is the best way to kill any infectious diseases that may be present in wild meat (Only groups 2, 3, 4 had this question in their questionnaires. It was added after the first session).**

**GROUP 2**

***The following section consists of free hand responses - where comments added is indicated**

(P1): Boiled ( it kills everything)

(P2): Both boiled and smoked because by long cooking and smoking it dry and very dry

(P3): Boiled and smoked

(P4): No answer

(P5): Boiled- Fully boiled and cooked with fresh hot pepper & onions

(P6): Boiled

(P7): Grilled- this helps to remove all the fat from the meat

**GROUP 3**

(P1): Boiled and smoked

**Comment added:** “The high temperature will kill what’s in the meat”

(P2): Boiled

(P3): Smoked

**Comment added: “**After an animal is killed smoking in my opinion is the best wat to preserve it for two reasons: killing of bacteria and long stay of meat.”

(P4): Smoked

**Comment added:** “You remove all the intestines and put them in the oven after spicing them.”

(P5): Smoked

**GROUP 4**

(P1)(P2)(P7)(P8): Boiled

(P4)(P6): Smoked

None: Smoked and Boiled

None: Grilled

(P3)(P5): No answer

7. **A) How do you feel about substituting local domestic meat instead of wild meat for this recipe?**

**Negatively**

**Group 1**

(P1)

(P2)

(P6)

**Group 2**

(P1)

(P2)

(P3)

(P4)

**Group 3**

P(P1)

P(P2)

P(P3) Comment: Because we are born in that area and associated with that meat

P(P5)

**Group 4**

P(2)

P(3)

P(4)

P(5)

P(6)

P(7)

P(8)

**Positively**

**Group 1**

(P3)

(P4)

(P5)

**Group 2**

(P5)

(P6)

(P7)

**Group 3**

P(4)

**Group 4**

P(1)

- - **No answer**

**Group 1**

(P7)

(P8)

**B) What if the wild meat comes from Europe? (Group 3 & 4)**

*** This question was added after the second focus group session as a result of discussions (as indicated by results from only group 3&4).**

**GROUP 3 -**

(P1) Negative. Meat from the forest is always sold. We should not make difference between continents

(P2) Negative.

(P3) Negative. Not really because we are always not used to the environment

(P4) Negative. I will not be comfortable because am used to what we have in Ghana

(P5) Negative. I can’t really explain it

**Group 4:**

P(1): Positive .It would be acceptable, considering the measures put in place assure consumers that the animals are killed in an appropriate wat

P(2): Negative about substituting for local meat and no comment about European meat

P(3): Negative. No comment about European sourced meat

P(4): Negative. No comment about European sourced meat

P(5) Negative.

P(6): Negative. It would not be acceptable

P(7): Negative. No comment “…”

P(8): Negative. Hygienically processed

1. **Do you have any concerns regarding the health risks that may be associated with wild meat originating from Africa?**

**GROUP 1**

(P1): No. I have concerns about eating meat in general (No answer)

(P2):No

(P3):No answer

(P4):No

(P5):No

(P6):No

(P7):Yes

(P8):Yes

**GROUP 2**

(P1):No

(P2):No

(P3):No

(P4):Yes

(P5):Yes

(P6);No

(P7);No

**GROUP 3**

(P1):No

(P2):Yes

(P3): No, because we are eating this through generations

(P4):No

(P5):Yes

**Group 4**

(P1):Yes

(P2):No

(P3): Yes

(P4): No answer

(P5): No

(P6):Yes

(P7):No

(P8): Yes

**9. What do you like about wild meat?**

- - 1. **Taste**
    2. A.Tradition and Culture
    3. B. Community Building
    4. C. Religion
    5. D. Health Benefits
    6. E. Other… explain

**GROUP 1**

(P1):A&B Comment: People who eat this meat cite these answers

(P2):E

(P3): No answer

(P4): A

(P5):A

(P6):A, B,C

(P7):A

(P8):A

**GROUP 2**

(P1):A, B, E

(P2):E

(P3):B, E

(P4)-B

(P5)- B

(P6)- B

(P7): A,B, E

**GROUP 3**

(P1): A

(P2):A

(P3):A,E

(P4):A

(P5):B

**Group 4:**

(P1): A

(P2): A

(P3): C

(P4): No answer

(P5): D

(P6): A

(P7): A

(P8): B

**10. What do you dislike about wild meat?**

**Group 1**

(P1): The way they are hunted are sometimes with poison

(P2): How to trap it

(P3): No answer

(P4):Nothing

(P5): Nothing

(P6):Nothing

(P7):Uncontrolled killing/Not knowing the source

(P8):How it is hunted

**Group 2**

(P1): Nothing

(P2): No answer

(P3): Nothing

(P4): No answer

(P5): Some methods of getting them

(P6):The preservation

P7: N/A

**Group 3**

(P1)- No idea

(P2)-Nothing

(P3)- The way some of them have been killed

(P4)- They are difficult to get

(P5): No answer

**Group 4:**

(P1): It can sometimes harbor germs due to the means of preservation

(P2): I do like wild meat

(P3): No answer

(P4): No answer

(P5): No answer

(P6): It’s very difficult to know how it was killed

(P7): No answer

(P8): What the animal eats

**11. How often do you consume wild meat?**

**Group 1**

(P1)(P5): Never

(P2)(P8): 0 times a year

(P4)(P7):1-2 times a year

No participants: 3-5times a year

No participants: 5-10 times a year

(P6): 10+ times a year

(P3) No answer

**Group 2**

(P2): Never

(P4): 0 times a year – P4

(P7): 1-2 times a year

(P3)(P5): 3-5 times a year

No participants:5-10times a year

(P1)(P6): 10+ times a year

No answer

**Group 3**

Never

(P2) (P3) (P5): 0 times a year

No participants: 1-2times a year

(P1): 3-5 times a year

No participants: 5-10times a year

(P4) 10+ times a year

No answer

**Group 4**

(P3) (P4) (P7) (P8): Never

(P6): 0 times a year

(P1) (P2) (P5): 1-2 times a year

No participants: 3-5 times a year

No participants: 5-10 times a year

10+ times a year

No answer

1. **Are there particular times of the year or special occasions during which wild meat is consumed here in the Netherlands? Explain:**

**Group 1**

(P1)-:No

(P2): No

(P3)-:No answer

(P4): No

(P5):No

(P6): No

(P7):No

P8- (P8):No

**Group 2**

(P1): No particular time/All the time

(P2): No answer

(P3)-:No

(P4): No answer

(P5):NA

(P6): No-we do it when we feel like it/we want to feel like home

(P7):No

**Group 3**

(P1):No

(P2):No answer

(P3):During traditional gatherings

(P4):No

(P5):No answer

**Group 4**

(P1:)No you can it purchase it anytime there is a need for it

(P2):I haven’t consumed meat here in the Netherlands

(P3):Never

(P4):No answer

(P5):None

(P6):I can’t explain it since I don’t know anything about it

(P7):No answer

(P8); Do not know

1. **If you buy it, how do you acquire AWM in the Netherlands?**

Through friends

Mail/Courier

Local Butcher

Local restaurant

Other… explain:

**Group 1**

(P1): Friends or bring it over themselves

(P2): NA

(P3):-Friends

(P4): Friends

(P5): NA

(P6): Friends

(P7): Friends or bring it over themselves

(P8): Local Ghanaian butcher

**Group 2:**

(P1): Local butcher/restaurant

(P2): No answer

(P3): I bring it myself

(P4): Through friends

(P5): Shops

(P6):Through Friends

(P7):Ghanaian shops

**Group 3:**

(P2) (P3)(P4)(P5):Through friends

None: Mail/courier

None:Local restaurant

(P1): Other … explain –I bring it from Africa myself

Through friends

**Group 4**

(P1): Through friends

(P2): I buy wild meat here in the Netherlands

(P3): I do not buy them

(P4): No answer

(P5): Through friends

(P6): I do not buy AWM in the Netherlands

(P7): Through friends

(P8): Through friends

1. **Is it more or less difficult to buy rare kinds of wild meat in the Netherlands than in West Africa?**

**Group 1**

(P1)(P2)(P3)(P4)(P6)(P7)(P8): More difficult

None: Less difficult

None: Equally as difficult

Don’t know: (P5)

**GROUP 2**

(P1):Less difficult

(P2): More difficult

(P3)- More difficult – shops often don’t have any

(P4)- More difficult

(P5)- More difficult

(P6)- More difficult

(P7)- Less difficult

**GROUP 3**

(P1)- More difficult

(P2)- More difficult

(P3)- More difficult because it is not allowed

(P4)- More difficult

(P5)- More difficult

**Group 4**

(P1)- More difficult

(P2)- Less difficult

(P3)- I don’t know

(P4)- No answer

(P5)-More difficult

(P6)- More difficult

(P7)-More difficult

(P8)- More difficult

1. **Is wild meat more expensive in the Netherlands?**

**Group 1**

**Y:Yes**

**N:No**

1. (P1)(P2)(P3)(P4)(P6)(P8):Y

2. None: No

3. (P5): No idea

4. (P7): Not allowed to be imported

If you answered yes, give an example of price per kilo per animal

(P1):Y -200% more

(P2):Y- 25 euros a kilo

(P3):Y- 25 euros kilo

(P4):Y- 100% more expensive

(P5): No idea

(P6):Y- 25 euros a kilo

(P7) :Imported by friends

(P8): Not allowed

**Group 2**

(P1): Y prices are 100% more than in Africa

(P2): Y from friends I heard it costs around 20 euros a kilo

(P3): Y – 15 euros a piece not sold in kilos

(P4): Y- I am not sure but I think it is around 30 euros

(P5): Y- at shop owners’ discretion

(P6): Y- 50 euros

(P7): Y- between 15 to 20 euros a kilo

**Group 3**

(P1)- Y – I don’t go for it.

(P2)- Y

(P3) -Y – Because you have smuggled it before bringing it. Price per kilo plus or minus 30 euros

(P4)- Y – Because a kilo can 20-25 euros

(P5)- Y

Group 4

P1- N

P2- Y- 20 euros a kilo

P3- I do not know

P4- No answer

P5- Y - 10 euros

P6- Yes

P(7) – Yes

P(8)- Yes, it is presumable because of its scarcity

1. **How do you or members of your community like for the wild meat to be prepared?**

**Group 1**

(P1)(P4)(P5)(P7): Boiled

P1)(P2)(P3)(P6): Smoked

Grilled

Other … Explain

(P5): Cooked in soup

(P8)- No answer

**G Group 2**

(P1): Boiled

(P2): Smoked

(P3): Smoked

(P4): Smoked

(P5): Smoked

(P6): Smoked

(P7): Grilled

**G**

**Group 3**

- (P1): Boiled, smoked
- (P2): Smoked
- (P3): Smoked
- (P4): Smoked
- (P5): Smoked

**Gr Group 4**

- (P1): Boiled
- (P2): Boiled
- (P3): Smoked
- (P4): No answer
- (P5): Boiled
- (P6): Boiled
- (P7): Smoked
- (P8): Boiled
