# Supplementary material for: Exploring the characteristics of a local demand for African wild meat: A focus group study of long-term Ghanaian residents in the Netherlands
Source: PLoS One. 2021 Feb 16;16(2):e0246868. doi: 10.1371/journal.pone.0246868 (PMC7886224; doi:10.1371/journal.pone.0246868)
Supplement: S1 File — (DOCX) [file pone.0246868.s005.docx]

**Africa Focus Group: Session 1**

**Coding key**

| **Pink** | **Luxury Item/Status symbol** |
| --- | --- |
| **D.Blue** | **Willingness to Pay (WTP)/ Bushmeat Price Comparison - Ghana vs. Netherlands** |
| **Yellow** | **Food Preparation/Taste** |
| **Green** | **How to avoid infection / Health considerations** |
| **Purple** | **Generation changes in bushmeat consumption** |
| **Red** | **Species** |
| **Orange** | **Local Demand & Trade/** **Methods of Acquiring meat locally** |
| **L.blue** | **Methods of hunting** |

******Notes on body language, gesturing, tone and volume have been made in blue italics transferred from notes taken by the interviewer during the discussions.******

**================================================================**

[crosstalk]

**Moderator:** I'm going to record this so that I can listen and write at the same time.

**Female participant:** Eto.

**Moderator:** How do I spell it?

**Male Participant:** Eto.

**Moderator:** Eto. Thank you.

[background conversation]

**Female participant:** The food is ready, please.

[crosstalk]

**Female participant:** [crosstalk] so that at least you have something, both to your list as well.

**Moderator:** Yes, and it's really nice. I really love it.

[background conversation]

**Female participant:** May God bless the hand that cooks the food and the stomach that is going to enjoy it.

**Participants:** Amen.

**Female participant:** And the pocket that provides.

**Moderator:** Is eto throughout Ghana, or in the north, or in the south, east, west? Is it the same dish?

**Female participant:** I think western, it's west, and over Accra. I think it's only the northern, yes.

**Moderator:** Okay.

[background conversation]

**Female participant:** It's everywhere.

**Moderator:** Everywhere?

**Female participant:** Yes.

[background conversation]

**Female participant:** It's everywhere in Ghana. [foreign language]

**Male Participant:** [foreign language]

**Female participant:** It's not everywhere that use it for-

**Moderator:** For this celebration. That particular--

**Female participant:** Yes. I think the northern, they don't, but the western, the eastern, the Ashanti.

**Moderator:** Oh, the Ashanti. I saw them at the party.

**Male Participant:** Southern coastal.

**Female participant:** The southern coastal I think, yes, mostly they use it. 90% of Ghanaian do.

**Moderator:** Yes. I'm excited.

**Male Participant:** [foreign language]

**Male Participant:** [foreign language] There is a place they're doing the dance with the Royals. I went there. I had to play a part. We haven't made any arrangement, so I just went there to see if it was going well, and it was.

**Female participant:** [foreign language]

**Male Participant:** [foreign language] I think that ambassador is there.

[crosstalk]

**Male Participant:** *The dilemma of a Ghost.*

**Male Participant:** *The dilemma of a Ghost.* I've read it. Do you know who wrote it?

**Male Participant:** Yes, Ama.

**Male Participant:** Ama Ata Aidoo.

**Male Participant:** [foreign word]

**Male Participant:** Is Ama Ata Aidoo the ambassador?

[crosstalk]

[background conversation]

**Male Participant:** H-O-R-N-D-R. **[unintelligible 00:02:58].**

**Female participant:** **[unintelligible 00:02:59].**

[background conversation in] [foreign language]

**Male Participant:** They [crosstalk] as mere hunters.

**Male Participant:** They the hunters. They're hunters. We're all, of course. At one moment, it was people **[unintelligible 00:03:26].**

**Male Participant:** [foreign language]

**Moderator:** Thank you very much.

**Male Participant:** [foreign language]

**Male Participant:** But Usman is not one.

**Male Participant:** Usman is not one.

**Female participant:** No, Usman is a westerner.

**Male Participant:** Oh, there is some **[unintelligible 00:03:40]** in, how do they call it? Even in century today.

[background conversation] [foreign language]

**Male Participant:** Are you enjoying it? Enjoying the meal.

**Male Participant:** [crosstalk]

[background conversation] [foreign language]

**Moderator:** [crosstalk]

**Male Participant:** [laughs] It's public.

**Moderator:** [crosstalk]

**Male Participant:** From that kind areas of Ghana. We used to not to have this **[unintelligible 00:04:26].**

[background conversation] [foreign language]

**Male Participant:** I just came. Before I had around 10 something, then I went at 12:30 I left the house, and **[unintelligible 00:04:50]** to where they're doing the culture manifestation, and then I took a taxi and came here.

**Moderator:** This one is made with what?

**Male Participant:** You can make it with **[unintelligible 00:05:15].** You can also make it with yam.

**Moderator:** With yams also. Okay.

**Male Participant:** Cocoa yam, [crosstalk].

[background conversation]

**Moderator:** What are the main vegetables and the main diet? The main vegetables. The ones that you see the most often in the food. Which vegetables are the more common?

**Male Participant:** Garden eggs, pepper, tomato, Spinach.

[background conversation] [foreign language]

**Male Participant:** Aubergine.

**Moderator:** Aubergine.

[background conversation] [foreign language]

**Male Participant:** It is aubergine. It's the same long thing, and then you have the--

**Female participant:** The long one is aubergine or what?

**Male Participant:** That's what they call it. They're the geun family. G-E-U-N. They're all the garden eggs. They all belong to the same family.

**Female participant:** **[unintelligible 00:07:14]** that they're called different.

**Male Participant:** They call the other one [foreign word] and then they have [foreign word]. [foreign word] is the aubergine.

**Moderator:** Are there areas of Ghana where it's spicier and not spicy, or it's the same?

**Male Participant:** It's the same [crosstalk].

[background conversation] [foreign language]

**Moderator:** Then everybody just uses different spices. Like in India, the south is spicier than the north. I thought maybe **[unintelligible 00:08:20].**

**Male Participant:** In India, they have thousands of--

**Moderator:** Spices, yes.

**Male Participant:** Thousands of them. You can't count their-- Especially in the south.

**Moderator:** Yes, I know. My husband worked a lot in India. When we go, then I can taste the really strong spicy food. It's quite strong.

**Male Participant:** Which part of India?

**Moderator:** Nick Stern, I don't know if you know Nick Stern with the Stern Report? Who does climate change. He is an economist. Used to be the chief economist of the World Bank. They have been working in a village called **[unintelligible 00:09:08]** for, now this is the third decade. They do property measurement. All throughout India, he does property mapping. That's why we taste a lot of a different food. Sometimes in the north and the south, the food is so different. In Ghana, there is no difference in spice. Like the spiciness.

**Male Participant:** It's only in the Accra region when you go and eat this kenkay and grand pepper.

**Male Participant:** In the north, we also have spices, but they prepare it differently. They use, what do you call it? Yes. It's all a question of taste. Someone likes it a bit spicier than the other. In the North, they have their cocoa and their porridge even full of different spices.

**Moderator:** Maybe it's what you grow up with. Because, in my family, at a very young age, my children asked tabasco sauce spice, because their grandfather, my father, puts it on everything, and I used a lot of spaces, so, at a very young age, they do it. I don't know of many other children of their age then. They learn at home, it's the same thing with the amount of salt. If you teach your children to add salt at a young age, by the time they are maybe in their twenties, they're adding a lot of sauce and they can't eat without adding a lot of salt.

**Male Participant:** Yes.

[background talk]

**Male Participant:** It's raining there.

**Male Participant:** But they told us it wasn't going to rain today

[laughter]

**Male Participant:** **[unintelligible 00:11:23]** the opposite of what they say.

**Moderator:** Do you all have a favorite grilling restaurant in Amsterdam?

**Male participant:** Amsterdam?

**Moderator:** Yes.

**Male participant:** Yes.

**Moderator:** Do you have a favorite?

**Male Participant:** Yes.

**Moderator:** Which one?

**Male Participant:** How many are they?

**Moderator:** [laughs] How many are they?

**Male Participant:** The World of foods.

**Male Participant:** Okay. The guy there cooks very well. Particularly--

**Male Participant:** There's also a kenkey spot.

**Male Participant:** A kenkey-- Will you call that a restaurant?

**Male Participant:** A spot.

**Male Participant:** Yes, but you get your kenkey, then go away.

**Male participant:** You can eat there.

**Male Participant:** You can eat it once.

**Male Participant:** Okay. If it is **[unintelligible 00:12:15]**, then you eat only kenkey, can you have--

**Female Participant:** There's one thing **[unintelligible 00:12:19]**

**Male Participant:** There's only one thing?

**Male participant:** No, you can get everything else.

**Male Participant:** Okay.

**Male participant:** It's a **[unintelligible 00:12:23],** so I don't know what your definition of a restaurant is.

**Male Participant:** Exactly.

**Male Participant:** No. My point is, I know the place. There's a place just by the roadside, you can buy kenkey and co

**Female Participant:** **[unintelligible 00:12:35]**

**Male Participant:** I didn't know it was a restaurant that you can sit and--

**Male participant:** It's not a restaurant, It's a **[unintelligible 00:12:40]**

**Female Participant:** [foreign language]

**Male participant:** Shut up and eat.

**Female Participant:** [foreign language]

[laughter]

**Male Participant:** Where's the other lady? There's one in the kitchen, right?

**Female Participant:** Is it? I was the only one in the kitchen.

**Male Participant:** You were the only one. No, no, I thought they were--

**Male Participant:** There were two ladies in the kitchen. Okay.

**Moderator:** I know every time that-- When I was living in America, over time, even the Mexican food changed. Then, it was called *Texmex,* so Texas, Mexican. That's what Americans thought was Mexican food, but if you go to Mexico and you eat Mexican food, you're like, "This is not Mexican food the way we know it because they change it."

**Male Participant:** Yes. The kenkey here is also different.

**Male Participant:** The Italians say it, the Chinese say it. If you go to a Chinese restaurant, what you have here is not the same as--

**Moderator:** Yes.

**Female Participant:** I was even telling Kufreh about it this morning, that the kenkey here to me, is not kenkey.

**Female Participant:** [foreign language] too much salt.

**Female Participant:** It's not the salt. the kenkey is made of corn, fermented corn, but that's not what they use here. In the UK, you can't sell this kenkey that you eat here. Nobody will buy it. Yes. The Kenkey in UK is exactly like the kenkey in Ghana.

**Male Participant:** Okay. Obviously, the UK's kenkey has also gone through a certain evolution. Let me put it that way.

**Female Participant:** No. Straight from Ghana, the corn and everything.

**Male Participant:** No, it's the quality we're talking about

**Female Participant:** The quality is the same. If you eat kenkey in the UK, you will never be able to differentiate the kenkey from the UK and Ghana, it's exactly the same.

**Male participant:** Is it because they--

**Female Participant:** They use the same ingredients.

**Male participant:** How did they get it?

**Male Participant:** They import the whole thing.

**Female Participant:** They import.

**Male Participant:** They don't import the kenkey, they import the maize. No, you cannot do that

**Female Participant:** No.

**Male Participant:** **[unintelligible 00:15:01]**

**Female Participant:** No, but even here. The maize is here, but I don't know why they don't use it, because if you go to the Ghanian shops, it's there. I buy it to make my banku, but when it comes to kenkey, they use something different. I don't know why.

**Male Participant:** Okay.

**Moderator:** Okay, so it's available, they just don't use it.

**Female Participant:** It's there, but they just don't use it.

**Moderator:** Is it more expensive maybe?

**Female Participant:** I think so.

**Moderator:** Okay.

**Female Participant:** I think so.

**Male Participant:** In Ghana, the temperature is different--

**Female Participant:** Kenkey has to be done with fermented corn.

**Male Participant:** Yes. Fermentation takes certain temperature into consideration--

**Male participant:** Maybe in Ghana, even the flies **[inaudible 00:15:45]**

[laughter]

**Male Participant:** True. Serious.

[foreign language]

[laughter]

**Male participant:** What's that?

**Male participant:** Little flies in Ghana too, contributed to the taste of the kenkey.

**Male Participant:** The flies?

[laughter]

**Male Participant:** It is the most nonsense statement I've ever heard.

**Male participant:** No. I won't agree with you. I affirm it.

**Male participant:** No, because these flies land on it and they deposit something on it.

**Male Participant:** On my kenkey no fly --

**Male Participant:** That is why the taste is different

**Male Participant:** On my kenkey, no fly lands on it.

**Male participant:** It's not the kenkey that you're eating to your stomach. It's during the preparation time.

**Female Participant:** This is rubbish.

[crosstalk]

**Male participant:** It is during the preparation. I can do it but it's supposed to be funny.

**Male participant:** Let's do it. Welcome ladies and gentlemen. Sandrella, this is the group. We are at less two or three. The called off last minute. Over to you. I wouldn't explain everything, I'll just leave it to you to tell your own story. They know it's about bushmeat.

**Moderator:** Yes. It is, so--

**Male participant:** How many people were here the first time that we met?

**Moderator:** Three. Just the three of you. Am I recollecting it? There was--

**Male participant:** No. You were here.

**Moderator:** Yes, that's four.

**Male participant:** We have--

**Male participant:** Imagining we have a different--

**Moderator:** Yes. I learned a lot that day. A lot. I didn't record it, and I was trying to write as fast as I could but I can't write and listen as well, as fast as I can, so I just record it so I can write it at home. Again, my name is Sandrala Moristana, I'm a student. My focus is public health and nutrition. I'm in the field of epidemiology. I work with **[inaudible 00:18:24]** who is good friends with Kwadi and Dante. Also, I think you all know him very well. Especially Kwadi, he does a lot of work at **[inaudible 00:18:40]** too. **[unintelligible 00:18:41]** is the-- For those of you who don't know who he is, he is an epidemiologist, very well known epidemiologist here, in the Netherlands in infectious diseases.

**Male participant:** You know him. Right?

**Male participant:** Who?

**Male participant:** **[unintelligible 00:18:56]**

**Male participant:** No.

**Male participant:** It's Okay.

**Moderator:** Former director of infectious diseases here, in the Netherlands, and he's done very, very good work with HIV as well.

**Male Participant:** You are speaking very fast. Sometimes I think it's difficult to--

**Moderator:** Sorry. My kids say that when-- I have four kids, and I talk very fast. Sorry. Just keep reminding me if I do that. He's done a lot of work here in the Netherlands, in HIV, and in Africa also. A lot of work in Cameroon. We designed a study that is similar to a study that was done in the United States, just getting to know more about food and culture, and nutrition related to African wild meat. I think a lot of people don't know very much about that kind of meat, who eats it? Why they eat it? Where they get it? What recipes? If you can exchange one meat with another and it doesn't change the recipe.

These things are very unclear to a lot of people- There just hasn't been a lot of [cough] studies on it at all, so then people make up their own stories and come up with their own conclusions. I think it's always better to do a proper study so that we can educate and learn something new. That's my study. It's just a focus group talking about different parts of that question and seeing what everybody has to contribute or what they think about it, and I can learn about it. I think that it's not just about the Ghanaian community, but also to learn- to also talk to other communities like the Congolese, Liberians, also, possibly the Cameroons, and see what's similar, what's different, that's my study.

I started it last year and this is the first part. We had a pilot where we had a really, really nice conversation. I learned a lot but I couldn't remember it all. It was a lot going on and I forgot to write stuff down, so I was just listening and I lost a lot of that information, unfortunately, in that pilot study.

Robert is here with me, he's an anthropologist. He's done a lot of work in Africa on a range of many, many different subjects, and he's here basically because I'm a new student and I have never really done a focus group before where I sit and I talk and I listen, so he's here to help guide me, to make sure I understand the process and how to stay organised. Otherwise, I'm just going to talk and listen and then nothing is ever, ever, ever- This study is not going to make any progress [chuckles] That's it.

**Male Participant:** Okay.

**Robert:** I'm just **[unintelligible 00:22:15]** this one, I'm not going to be following her around.

**Moderator:** Yes.

**Robert:** I'm just going to give her some suggestions, after which maybe you could do it like this or you should have asked that, but otherwise, I'm not going to be following her around for the discussions and stuff.

**Male Participant:** Are you Dutch?

**Robert:** I'm-- I've got a dutch national passport, if that's what you mean.

[laughter]

[crosstalk]

**Male Participant:** Not the passport.

**Male Participant:** Who are you?

**Robert:** Well, I'll tell you him if he wants to know but I'd like to- [crosstalk] You sound American, right?

**Moderator:** I'm so sorry.

**Multiple Speakers:** No.

**Male Participant:** Don't you worry.

**Robert:** I originally come from South Africa but I left South Africa for almost 50 years ago.

**Female Participant:** Wow.

**Ass. Moderator:** I have lived in the UK-

**Female Participant:** Yes, because you sound **[inaudible 00:23:04].**

**Robert:** -and in the Netherlands for a long time. I've also lived in different African countries for about almost 20 years. I've lived all over the place.

**Moderator:** I am American but I have a French passport. My mother is French; they live in France.

**Male Participant:** Okay. You?

**Male Participant:** I'm Ghanaian, I'm Dutch. He wouldn't say so. I have a dutch passport.

[laughter]

**Male Participant:** Are you **[unintelligible 00:23:28]** him?

**Male Participant:** No, are you Ghanaian or Dutch?

**Male Participant:** I'm both.

**Female Participant:** No.

**Male Participant:** Every human being has multiple identities. Okay.

**Male Participant:** What are the rest? [laughs]

**Male Participant:** What else do you want to know?

**Male Participant:** No, you said multiple but that doesn't mean--

**Male Participant:** It depends-it depends. Do you want **[unintelligible 00:23:46]**?

**Female Participant:** Let's not divert because if you start here--

**Multiple Speakers:** No, no, no.

**Female Participant:** [laughs]

**Male Participant:** It's part of the [crosstalk].

**Male Participant:** No, because you have many identities. You have your-- I can identify myself as a teacher. I can identify myself as a student. I can identify myself as Dutch,

Ghanaian, Christian, Muslim, Traditionalist, all of them. Ga, Fante, everything.

**Male Participant:** Do you eat bushmeat?

[laughter]

**Male Participant:** I have a right to remain silent.

[laughter]

**Moderator:** **[unintelligible 00:24:13].**

**Male Participant:** A girlfriend of mine called me and said, "Oh, Nii, I wish you were in Ghana, so you can eat some of this soup I'm eating now." "So, what kind of soup is this." She said, "This is light soup with Kusie." Kusie is not even the-- It's rat; a kind of rat. Bigger than-

**Robert:** It's not the ones we have here?

**Male Participant:** It's a rat?

**Male Participant:** It's a rat. It's a rodent.

**Male Participant:** No, no, no. I resist. It's good that he explains because [crosstalk]

**Male Participant:** Talk about rat, you look at the one **[unintelligible 00:24:43]**

**Male Participant:** Yes, it's not the ones here. They're huge, they're big, and-

**Male participant:** Oh.

**Female Participant:** [foreign language].

**Male Participant:** I'm not talking about guinea. I'm not talking about the--

**Male Participant:** Hold on **[unintelligible 00:24:52]**

**Male Participant:** -the grasscutter. I'm talking about the other ones. I just said, "You're inviting me to come and eat Kusie light soup?" She said, "Yes." I started laughing [laughs] Because I've never eaten Kusie before. I've had grasscutter, I've had antelope, but I've never had Kusie. Kusie belongs to the class bushmeat.

**Male Participant:** Let's go back to your- Rat is rat.

**Moderator:** What's Kusie, by the way?

**Male Participant:** Whether you're in America or your're here. I've seen rats here, they're the same. [crosstalk]

**Male Participant:** It's not true.

**Male Participant:** No, no, no. It's not.

[crosstalk]

**Male Participant:** Do you know that animals-

**Male Participant:** There's a difference between Kusie and rat. Rat--

**?Man:** [foreign language].

**Male Participant:** There is a difference. There is mouse. Yes-

**Male Participant:** **[unintelligible 00:25:40]** a mouse, I know

**Male Participant:** There is a mouse.

**Male Participant:** **[unintelligible 00:25:42].**

**Male Participant:** Mouse and rat-- Rat is Kusie. It's a senior brother of mouse.

[laughter]

**Male Participant:** No, no, no.

**Male Participant:** They're in the same family.

**Male Participant:** You people, you want to be polite.

**Female Participant:** No, no, no.

[crosstalk]

**Male Participant:** It's not true. Do you know the animal, Capybara?

**Male Participant:** Do you know **[unintelligible 00:26:01]** Capybara?

**Male Participant:** Do you know it looks exactly like the grasscutter?

**Moderator:** What is a grasscutter, I'm so sorry.

**Male Participant:** A grasscutter is- it's a rodent in Ghana-

**Male Participant:** It's not a rodent. Oh, come on.

[crosstalk]

**Female Participant:** It's in the family.

**Male Participant:** It's not a rodent.

**Male Participant:** No, it's not a rodent. Okay [crosstalk]

**Male Participant:** Grasscutter eats only grass and greens, but a rodent eats- You're talking about a mouse.

**Male Participant:** Paper and everything. Okay.

**Male Participant:** Yes.

**Male Participant:** It belongs to the-- I've forgotten the-

**Male Participant:** It's a big-- It's like--

**Male Participant:** I've forgotten the classification.

**Male Participant:** It's almost-- It's like half the size of a goat.

**Moderator:** It's big.

**Male Participant:** Yes, it's big.

**Moderator:** Okay.

**Male Participant:** Yes. Now, the Capybara-

**Male Participant:** It looks like goat but it's very-- Goat has long legs and they don't have **[unintelligible 00:26:47].**

**Robert:** Cane rat.

**Moderator:** A cane rat?

**Robert:** Cane rat.

[crosstalk]

**Participants:** Yes.

**Male Participant:** That is very **[unintelligible 00:26:55].**

**Moderator:** That's a grasscutter? That's what a grasscutter is called?

**Robert:** Big front teeth.

**Multiple Speakers:** Yes.

**Moderator:** Okay, okay, got it.

**Male Participant:** No, but there's also [foreign language]. Don't make a mistake.

[crosstalk]

**Male Participant:** [foreign language] has got thorns--

[crosstalk]

**Female Participant:** [foreign language].

**Male Participant:** No.

[crosstalk]

**Male Participant:** Porcupine is also different.

[crosstalk]

**Male Participant:** [foreign language] is hedgehog.

[crosstalk]

**Male Participant:** Heard of hedgehog [foreign language] in Ghana.

**Male Participant:** [foreign language].

**Female Participant:** [foreign language] is different from grasscutter?

**Multiple Speakers:** Yes.

[crosstalk]

**Male Participant:** [foreign language].

**Female Participant:** [foreign language] Okay.

**Male Participant:** [foreign language] is nicer than [foreign language].

**Moderator:** How do they catch them?

**Male Participant:** Which ones?

**Moderator:** The grasscutters or-

**Male Participant:** They shoot them. They trap them.

**Male Participant:** They don't shoot grasscutters.

**Male Participant:** I've eaten [crosstalk] They shoot them.

**Female Participant:** Yes, yes, yes.

[crosstalk]

**Female Participant:** They do. [crosstalk] The hunters.

**Male Participant:** They shoot it. They know how to shoot it so that you don't spoil it.

**Female Participant:** Yes.

**Male Participant:** We've had [foreign language] at home. Agya Kojo and then aunty will be preparing it and they will go like **[unintelligible 00:27:57]** You see the pellets-

**Female Participant:** **[unintelligible 00:27:58].**

**Male Participant:** -from the back--

**Female Participant:** Yes, yes, yes. [foreign language] that's what cracked his tooth.

[crosstalk]

**Male Participant:** Most of them, the small ones, you catch them.

**Moderator:** Okay, because they're **[unintelligible 00:28:11]?**

[crosstalk]

**Multiple Speakers:** Traps.

**Moderator:** They're **[unintelligible 00:28:13].** They're fast? Are they fast?

**Female Participant:** We trap them.

**Female Participant:** Trap them.

**Moderator:** Yes. Are they very fast?

[crosstalk]

**Male Participant:** [foreign language].

**Male Participant:** How is it like?

**Female Participant:** [crosstalk] drop it on them and [crosstalk]

[crosstalk]

**Female Participant:** You mean the one in the hole?

**Male Participant:** That one they put-

**Female Participant:** The one in the hole.

**Female Participant:** Fire.

**Male Participant:** They start a fire and then they blow the smoke into it. [crosstalk] They come out of the hole or another hole?

**Female Participant:** The other hole, yes.

[crosstalk]

**Male Participant:** That is the rat **[unintelligible 00:28:38]?**

[crosstalk]

**Male Participant:** That is the rat. [crosstalk] That is Kusie.

[crosstalk]

**Male Participant:** That's Kusie. Even the grasscutters, we've been running after them with sticks, making fire, and then we will catch them-

**Male Participant:** yes. It's because-- in the dry seasons they always find it difficult to get a place to hide, so people who go after them, they make fire around them, they go and stand and they would be hitting them with-

**Male Participant:** They would come out and then they would catch the [crosstalk]

**Male Participant:** They also use dogs-

**Male Participant:** In my village, in the bush areas, it's a different thing. They come across they come to-- maize farms. When they come to maize farm they make a-- around it-- the traps-

**Male Participant:** Traps.

**Male Participant:** -around it. That is where we get them.

**Male Participant:** That's one way-

[crosstalk]

**Male Participant:** Then we can shoot them-

**Male Participant:** That's one means of getting them. Where you have the farm-

**Male Participant:** I've mentioned three.

**Male Participant:** Yes. I think, usually, people would go and search for them. You will use-- Normally they live in holes, so you put some fire in the hole and start fanning it, and then the-

**Male Participant:** The grasscutter?

**Female Participant:** Not the grasscutter.

[crosstalk]

**Female Participant:** You mean--

**Male Participant:** Yes, because they cannot stand to smoke, they will come out.

**Male Participant:** Not grasscutter?

**Female Participant:** No, [crosstalk] bush rat.

[crosstalk]

**Female Participant:** Bush rat.

**Male Participant:** The grasscutter doesn't live in holes, but they also-- they don't live in holes. They live in the--

**Male Participant:** In, what do you call it? Indentation in the ground. How do you call--?

**Male Participant:** Yes. I think that they rush out, all of them run when they see smoke because it is disaster coming. They will get burnt if they don't go.

**Female participant:** One thing I must say, I think most animals that we've got at home like cats, dogs, we've got the bush one of it as well. Many a time they don't eat the ones at home, and rather, we eat the ones in the bush. Many pets that we've got at home, we hold in the European place, that they're pets, but we've got the same identical in the bush as well. Something like monkey, people have them as pets at home, but we got bush-monkeys as well.

**Male Participant:** The ones at home, we eat them too.

**Male participant:** People eat the ones at home too.

**Female Participant:** Many [crosstalk]--

**Male Participant:** No, they don't.

**Male participant:** Not everybody. No, not everybody, but people who eat--

**Female Participant:** No, but 90% of people don't eat the ones at home.

**Male participant:** People who eat dogs, they eat dogs even at home.

**Female Participant:** 90% of people who have these animals at home, does not eat the bush ones.

**Male participant:** [crosstalk] someone like me--

**Male participant:** May I say something? When we talk about pets at home, what is pet for someone is meat for the other. You cannot say, if it's at home, they don't eat it. Some eat it, some don't.

**Male participant:** I understand where he is coming from. How would you consider goats and sheep? Because they are all--

**Male participant:** You can classify them as my pets. Chicken, you can classify them as pets till Christmas, then they will see their fate.

[laughter]

**Female Participant:** What I'm saying is, all these one, we've got them in the bush as well.

**Male participant:** Yes, but you are saying-- You made an assertion which is not completely true. You said, the ones at home, we don't eat them, and the ones in the bush, we eat them, but the ones at home, we eat them. That's what we are telling you.

**Moderator:** Which kinds of monkeys do you eat, and which kinds do you not eat?

**Male participant:** Every monkey. [crosstalk] All the monkeys we eat them.

**Moderator:** Why? Because it tastes good?

**Male Participant:** No. **[inaudible 00:32:23]**. Let's not make any-- Those who eat monkeys--

**Male Participant:** Hold on.

**Male Participant:** -- those who eat monkeys, eat monkeys, whether it's from the bush or at home.

**Male Participant:** That's not true.

**Female Participant:** I've ate monkey before, but I've never eaten the house one before.

**Male Participant:** That's not. There are--

**Male Participant:** Then you should--

**Male Participant:** Now hold on. We can get into the details, but maybe just for the process, right? We will get to the talk, so we can talk about the monkeys we eat and--

**Male Participant:** I don't eat-- I've never consciously eaten a monkey.

**Male Participant:** We would talk about the cats that we eat and we don't eat. By the way, those at home, we brought them from the bush, so they were once in the bushes. They have their counterparts still there. For this study, how many groups discussions do you want to have?

**Moderator:** 3, or 4 with groups of 10. The last time we spoke, there was a gentleman, and I think he mentioned something that he was concerned about, which eventually I wanted to talk about with how to catch the animals. He said that he was getting worried--

**Male Participant:** We would get into that, but of course, we are here so we also have to look at how we are going to organize the [crosstalk]--

**Moderator:** Yes, that's right.

**Male Participant:** We are here. How many people are here now? Eight?

**Male Participant:** Yes.

**Male Participant:** Is this counting towards the-- How many do you say you want to have? How many--?

**Moderator:** Three or four.

**Male Participant:** Is this part of the--

**Male Participant: [unintelligible 00:34:04]** you said two. You're trying to say three [crosstalk] saying four.

**Moderator:** No, no. In the--

**Male Participant:** I'm very clear on that. You said two, then it became three, now you said four. I think two would be too little.

**Moderator:** I'm feeling my way. The first one I felt was going to be count, but it was a pilot, because that was my very first time. I didn't take any notes, they didn't-- That's true so then--

**Male Participant:** Let's just establish it. Do you want three or four?

**Moderator:** Okay,

**Male Participant:** How many?

**Moderator:** Three would be fine. Three would be great.

**Male Participant:** Okay, so this is one, and then, can Ochima help organizing--

**Male Participant:** No, the second one is taking place at **[unintelligible 00:34:49]** place. I've already done [crosstalk]It would be outside. That is the next week Saturday about the same time. Is it possible to get 10 people?

**Male Participant:** I think for the 10 people, that was also a miscommunication between us. I thought 10 it included all of us. Now, I've been made to understand that we should be outside to organize, 10 outside this group.

**Male Participant:** Who should be outside?

**Moderator:** Robert and I, because Robert--

**Male Participant:** That's what I understand, but--

**Male Participant:** You said there'll be two of us. That's what she said earlier, but the two of us were outside.

**Moderator:** Well, maybe that's not--

**Male Participant:** You have to get very clear.

**Moderator:** Yes, I did say that, because if we keep counting the two of you, then that means that it's less new participants, but that doesn't have to be. The design is just to have 2, 3, 4 groups of 10, and I'm finding my way. The reason why it's not clear to you is because it's not entirely so rigid to me either. We have some movement. I have to adjust to who's available too.

**Male Participant:** It's also reasonable that we are not always there because we are counting ourselves in all the groups. If we are here today in the next time-

**Male Participant:** We don't have to be.

**Male Participant:** -even if you are there, we should [crosstalk] part of you as well. That one, I think you are right.

**Moderator:** I think, maybe, yes, but it's just an idea.

**Male Participant:** The next group is on--

**Robert:** I think, five or six people excluding you guys, I think is also enough.

**Male Participant: [unintelligible 00:36:30]** where the next group is--

**Male Participant:** No, but then the question is-- We don't have to be if [crosstalk] next time we are not part of it, but I think for today, we can be part of it, right, or not?

**Moderator:** Yes, absolutely. I would like that. I find it very helpful.

**Male Participant:** Okay. This is one, and then-- The next Saturday we're at **[unintelligible 00:36:53]** place. Then we have to see whether **[unintelligible 00:36:58]** can organize. The gentleman who joined us for the first time, if he can organize the third one. I can reach out to him and see if he would do it. Then, we have the three. Then, would the topics be the same for all the three?

**Moderator:** Yes, I think. Yes, I think it should be, because then I can organize them. If they have the same questions, then I'd say, "Okay these many people in total answered it this way, and these people said this together, and these people-- This number of people said--." Otherwise, you can't compare groups, until everybody has something different to say.

**Male Participant:** For the meeting, you would like to record, right?

**Moderator:** Yes, because then I--

**Male Participant:** Are you recording this?

**Moderator:** Yes, I just, I'm recording it.

**Male Participant:** Yes, okay.

**Male Participant:** Let's go back to the subject. We were clear with the basics?

**Male Participant:** Yes, I'm clear with the basics, but I want to make one more point. For the discussions, you have some key questions that--?

**Moderator:** I do.

**Male Participant:** Yes, okay.

**Moderator:** I have a diagram here, and then I have in my head some questions. It's based on the study in Liberia, and the Liberian community in America, so that we can then make a comparison about how different groups of people--

**Male Participant:** Yes, but we Ghanaian cook more delicious soup than **[inaudible 00:38:32]**.

[laughter]

**Male Participant:** Obviously--

**Moderator:** I will make sure I [crosstalk] that.

**Male Participant:** It's true. No, it's true.

**Moderator:** I promise to include that--

**Male Participant:** They always they always eat rice and peanuts. No. Rice and--

**Male Participant:** Sugar.

**Male Participant:** Now they even have cassava leaves [crosstalk]--

**Male Participant:** Cassava leaves.

**Male Participant:** Then, they have these pork legs. That is a delicious meal.

**Male Participant:** Okay. We use rice and coconut stew--

**Male Participant:** Cocoa yam stew. You change it up, but they are okay.

**Male Participant:** None **[unintelligible 00:39:09]**, so then we can-- You were not there the last time we met, right? The part of the discussion is also to understand the culture, and see how people adapt, if they are in a society for some time, yes? You have Ghanaians, and I think I'm going to say, and his group is a nice example where, last week they did this event, and instead of giving us **[unintelligible 00:39:39]**, they gave us donuts.

[laughter]

**Male Participant:** You could see that they are adapting to the Dutch culture.

[laughter]

**Male Participant:** All you are saying is very, very true. She **[unintelligible 00:39:54]** I was quarreling with them. I said, "How on earth did you think about making the donuts?" Finally, I chipped the chicken in.

**Male Participant:** Yes, so that's also the other part.

**Male Participant:** Yes, I completely understand it **[unintelligible 00:40:10]**.

**Male Participant:** I define culture as the sum total of the interaction within a certain community based on what is there in a peoples' life. You can see their food culture, you can see their dress culture, you can see many aspects of culture, and if the culture, the things that made it possible for us to enjoy certain things back home in Ghana, there are factors here mitigating against doing the same things here. When you get here, you definitely have to adapt, and so this is an example, what you just mentioned--

**Female Participant:** Such as not having the proper kenkey. [laughs]

**Male Participant:** Sorry, do you see this as progress?

**Male Participant:** Progress is also a relative thing. Does it serve the purpose at that time? If it does, yes. In the beginning, the transition phase, people will kick against it, they will not see it as part of the culture, but as it goes on, it becomes accepted and it becomes a part of it.

**Male Participant:** Okay, so specifically they made donuts instead of giving us eto. Is it progress?

**Male Participant:** I think it's progressive--

**Male Participant:** No. Not always.

**Male Participant:** It's progressive because you see, how we're eating the eto, some people will eat with their hands, some will never accept it to eat any other thing with their hands. That is one. Two, the spoons we were using to eat would have been very, very complicated for us to serve 450 people with spoons. So I think it was progressive that they do the--

**Male Participant:** I will use the word progressive. The adaptation, whether it's good or not you can say it, and then whether it's good or not is a question of how many people do it and how many people accept it, and how many people believe in it, but yes, if you cannot find planting and the time to make it and you can find the time to--

**Male Participant:** **[unintelligible 00:42:02]?**

**Male Participant:** Yes, sort of.

**Female Participant:** I think I quite agree with you in terms of how many people do it and how many people accept it, because if for instance, I have been to a Nigerian wedding in UK, and they served ebà at the reception--

**Male Participant:** Which is pounded wheat--

**Male Participant:** No, pounded yam [crosstalk]

**Male Participant:** Pounded yam [laughs].

**Female Participant:** Whilst everybody sits and eats with their hand.

**Male Participant:** And they are proud to do it.

**Female Participant:** And they were so proud.

**Male Participant:** That is again the identity question, the identity issue.

**Male Participant:** Likewise, Indian wedding where there's long table and everybody was eating with their hands.

**Male Participant:** Sometimes it's not convenient to eat with your hands.

**Male Participant:** You said sometimes.

**Male Participant:** I went to India, and it was a very big conference--

**Female Participant:** In the UK, this was a wedding in the UK, and I'm telling you, they served the table and they ate with their hands.

**Male Participant:** Okay, they served meals et cetera. There were no cutleries. Everybody had to use-- from professor to student, they all used their hands.

**Female Participant:** What about **[unintelligible 00:43:06]** adaptation times also, the food depends on the time because there are sometimes when you can't eat eto.

**Male Participant:** Yes, I agree.

**Female Participant:** Sometimes you can't eat eto. You eat it maybe in the morning or in the afternoon--

**Male Participant:** Okay, but not in the night.

**Female Participant:** In the night-- Even the eba you were saying, maybe they ate it in the afternoon, because if you eat eba in the night, how are you going to sleep?

**Male Participant:** Some people eat it and sleep.

**Female Participant:** Some people will eat--

[crosstalk]

**Female Participant:** Not everyone will eat.

**Male Participant:** Point well taken. Okay, so let's then kick off, and Sandrala, where do we start?

BEGINNING OF SESSION -------

**Moderator:** Okay, so last time we met, there was a gentleman that was seated in your place and he was concerned, if I recall correctly, because some of the wild meat that they were eating, he was talking about how they were using poison as a new way of hunting for this wild meat, and he was saying, "Okay, is somebody going to do a study about that because it's a public health issue?" Is that something that you have heard about? Are they using that as a way of hunting for--

**Male Participant:** Yes. That's more reason why I do not take bushmeat which is not coming from a hunter I know.

**Moderator:** Okay, so that's another question, so you have your trusted hunters or members of the family who could--

**Male Participant:** Yes, a hunter I know well.

**Moderator:** What about the markets? Would it be [crosstalk] like there's abattoir?

**Female Participant:** That's difficult to do.

**Moderator:** If you have somebody that you trust there?

**Male Participant:** No. I don't trust--

**Male Participant:** They buy and sell.

**Male Participant:** Yes, people bring it to them. You wouldn't know how they got it.

**Male Participant:** I don't, but of course there's people who eat them. **[unintelligible 00:45:10].**

**Moderator:** What is it when they singe the fur off, so what is that process? What does that do to the taste of the meat? Is it just a more flavorful taste to the meat?

**Female Participant:** [crosstalk] some use hot water. Some use hot water for the grasscutter and all those things. Some people use hot water and some use fire.

**Male Participant:** Don't they roast them together with the--

**Female Participant:** No, some use hot water--

**Male Participant:** Some use hot water and **[unintelligible 00:45:49]** used to use hot water to **[unintelligible 00:45:48]** the fur off, and then they would roast it.

**Female Participant:** Before they put it on the fire, yes, but they cut it--

**Male Participant:** It's different from what the-- The goats and the sheep.

**Male Participant:** No, I know goats they smoke it.

**Female Participant:** That's **[unintelligible 00:46:04].**

**Female Participant:** That's not **[unintelligible 00:46:05]**

**Male Participant:** With the sheep it's different. It is, how do you call it? Smoked or whatever, but I can tell you it's not done that way.

**Female Participant:** No, they use hot water to **[unintelligible 00:46:24]**, yes.

**Male Participant:** That's what I'm used to at home, but the ones they sell at the roadside if you're going to Cape Coast, always they have burnt off the skin, and that is not roasting. You see, burning off the skin is just taking the hair off, and then they will cut it, remove the interest and then they put it on fire, and that's what we call smoking it. It is curing it to make it last longer. Of course, it gives it that unique taste--

**Moderator:** Flavor.

**Male Participant:** Flavor.

**Moderator:** More flavor in the smoked version than in the boiled, or the grilled.

**Male Participant:** Yes. They have different tastes, yes. It's just like in this country we have what they call smoked salmon. In fact, they call it steamed here. If you taste, if they use different kinds of wood, to give you different tastes.

**Male Participant:** No, but it depends on when you get it. When you buy it the whole animal just--

**Female Participant:** Fresh from the person.

**Male Participant:** Then you eat it--

**Female Participant:** You don't need to put it on the fire, yes.

**Male Participant:** Then they sell them also along the road where they might have smoked it already. That's a good thing, and you can also buy them smoked or you buy them fresh, just [crosstalk]

**Female Participant:** Also the smoking, I think the storage is also part of it because they cannot keep it longer, so the moment they smoke it then it's--

**Female Participant:** For skinning, it's not very popular. The skinning it's to the best, it's not very--

**Male Participant:** No, who talked about skinning? We don't skin them.

**Female Participant:** No, [crosstalk]

**Male Participant:** No, if you use hot water--

**Male Participant:** [crosstalk] use hot water to skin. We use hot water to take off the hair, not skinning. Skinning is to remove the skin.

**Female Participant:** No, not the skin, only the hair.

**Male Participant:** No, just the hair. You just dip it in it, and then you go like this and then you--

**Male Participant:** Hold on there, so you have the same animal. One is smoked, and the other one they use hot water--

**Male Participant:** Hot water to peel off the--

**Male Participant:** No, you could--

**Male Participant:** Yes hold on, and you are selling these two on the market, which one will people go for?

**Participants:** The smoked one.

**Female Participant:** The one they use hot water only to take off the fur.

**Male Participant:** The hair.

**Female Participant:** Okay. Then afterward they cut it, and then they put it on the fire--

**Male Participant:** They smoke it again.

**Female Participant:** Okay, when they use hot water, it doesn't naturally take the skin out.

**Male Participant:** No. Not the skin, it's just the hair.

**Female Participant:** No, only the hair.

**Female Participant:** It must be a very good technique to hot--

**Male Participant:** No, but the bigger ones, they sometimes take their skin off because they use the skin for something else. Am I right?

**Female Participant:** Yes.

**Male Participant:** You mean bigger animals?

**Male Participant:** Yes.

**Male Participant:** Yes, but we're talking about grasscutter--

**Male Participant:** We're talking about grasscutter [crosstalk]

**Moderator:** We can talk about anything because I don't know all the different species, and that's actually a very-- maybe talk about--

**Male Participant:** Yes, so let's try and exhaust the grasscutter before we go to antelope because we have antelopes this size or [foreign language] or we have this

size [foreign language] big ones, and this is the type that they say [foreign language] This one, we have an antelope this big, and they don't normally kill it.

**Male Participant:** Let's finish the--

**Male Participant:** Okay, we'll come back to that.

**Male Participant:** Yes. No, but for the research, technically these are not necessarily relevant. The question was about whether when you smoke the meat it affects the taste. Also, some of you are also saying that if-- you have twos different types. One is smoked and the other one they just use hot water to get the fur off, the one that they have smoked will get a higher price because people prefer the taste. Then the next discussion was about there are some bigger animals that they normally take the skin off.

**Male Participant:** They peel the skin off.

**Male Participant:** Yes, because they use the skin for something else.

[crosstalk]

**Male Participant:** Apart from the taste and then people’s preference for either smoked-

**Female Participant:** The fresh one.

**Male Participant:** - one or the fresh one, some people react to fresh meat and therefore will not take the fresh one **[unintelligible 00:50:57]**.

**Male Participant:** They are allergic to the fresh meat?

**Male Participant:** To an extent. Even when it comes to fish, they will prefer to take- How you call it? [crosstalk] Again, I think people are becoming a little health conscious in Ghana. Aside the bushmeat, any other meat including the fish they will want to smoke it before eating to reduce the level of fat. However, let me indicate quickly that with the bushmeat it is healthy or it is seen and appreciated as a healthier meat because of the running that the animals do. They are more skinny. They are not as fat--

[crosstalk]

**Moderator:** It’s lean?

**Male Participant:** Yes, it’s thin. The quality of the meat is lean as compared to what is grown at home. Even with the grasscutter that she was talking about, these days it is reared at home.

**Moderator:** How is that compared to the domestic meat that can be found in the Netherlands?

**Male Participant:** Well, are you talking of the fat content?

**Moderator:** Yes.

**Male Participant:** The fat content is higher here in Holland, yes.

**Moderator:** Here in Holland?

**Male Participant:** Yes.

**Female Participant:** Not just here even in Europe in general.

**Male Participant:** Some people prefer drier meat.

**Moderator:** That’s what they said in the last meeting. They said that they trust African wild meat better than the domestic meat, because the domestic meat-- Is that how you feel?

**Male Participant:** Exactly. It is horrible in Ghana. I go to Ghana and I see chicken and parts of chicken imported from Holland in the sun on the market for so long. They cook it and they sell it. I will prefer akrantie if I could eat it than to eat chicken that has been imported, been in the fridge for so many-- freezer for so many months and then on the market. Then you go eat. No. Traditionally, bushmeat set a very healthy-- give a healthy choice. The animal is sick it will die, then nobody will see it. Well, that’s debatable, [laughs] but then if you get a healthy one on the market, very fresh--

**Female Participant:** There’s also one scenario, because the meat is smoked, even if it’s diseased--

**Female Participant:** It's dead.

**Female Participant:** It’s dead-

**Female Participant:** The smell is--

[crosstalk]

**Female Participant:** - smoking it kills the germ. Unlike you go to the market here and buy the fresh meat, the blood contains whatever disease that the animal has got. You come home and if it’s not well cooked or if it’s not well preserved, you will transport the same germ into your body. Once it’s smoked-

**Male Participant:** Cooked.

**Female Participant:** - and cooked and everything to a certain degree, because most germs died at a certain temperature. Once it’s smoked at a certain temperature, one assume that the germ is killed. It’s more healthier for us than eating just the slaughter house--

**Moderator:** Yes, that's what your colleague said last time, that his impression of the meat here has hormones or it’s in these big factories where there is more of a chance of diseases within a population, very close population. I understand that. That makes sense.

**Male Participant:** I have an anecdote. I went with a girlfriend about 30 years ago to go and have dinner in a restaurant. They asked me how I wanted my beef. It was my first time-

**Male Participant:** Here or--

**Male Participant:** - here.

**Male Participant:** Okay.

**Male Participant:** Then they brought beef which was half cooked.

**Female Participant:** Half cooked.

**Male Participant:** The blood was dripping from it. I called them and I said, "This thing is not ready." I said, "No, I cannot take it. This is bloody. It is raw." They said, "Yes. How do you like it?" I said, "I want it cooked. Please, cook it for me."

**Male Participant:** [laughs]

**Male Participant:** It came back and it was still not very cooked.

**Moderator:** In France, nobody in my family-

**Female Participant:** Will eat.

**Moderator:** - will eat a steak or anything that isn’t bleeding.

**Female Participant:** Can you imagine?

[crosstalk]

**Moderator:** Also, it is steak tartare.

[crosstalk]

**Female Participant:** If there’s a germ in there, obviously, the germ hasn’t been killed. It’s still there.

**Moderator:** They eat steak tartare. My mother eats it almost once a week. It’s raw hamburger meat. It’s raw with an egg on top.

**Female Participant:** In the olden days, many people didn’t have refrigerators and fridges and things like that to store our meat and things like that, but once it’s smoked, you can preserve it for months and months.

**Male Participant:** I don’t think that we cook it at high temperature because we want to kill germs.

**Male Participant:** No.

**Female Participant:** No, subconsciously. No.

**Male Participant:** Those are afterthought. You can read that into it.

**Moderator:** Okay.

**Male Participant:** It wasn’t because you want to. It’s just what people were used to. Maybe--

**Female Participant:** For preservation.

**Male Participant:** Yes. Not to kill germs, because when people didn’t have refrigerator, they would cook the soup and then sometimes put the soup-- You cook it well and then you put it outside. What you eat, the leftover, you put it outside, and then the next day you will torch charcoal, so that you cannot **[unintelligible 00:56:22]**.

**Female Participant:** Don’t we also say that it’s [foreign language]

[crosstalk]

**Male Participant:** There are hygienic things you do- [crosstalk] It’s not to kill germs. It was more to preserve it, because you would get some-- Not really that you will get disease but because you cannot keep it for a long time. You acquired that taste. Then you have this dimension where the point you made about raw fish. There are some people who will not take it, because, not necessarily because of allergy but because-

[crosstalk]

**Male Participant:** [foreign language]

**Male Participant:** No, it’s not really an allergy. [crosstalk] It’s like they don’t like it, because it’s raw. It’s the same that I don’t like herring because it's raw. I won’t take it. I will just vomit with the taste of it.

**Male Participant:** It’s nice.

[laughter]

[crosstalk]

**Male Participant:** I think people wouldn't--

**Female Participant:** No, amane is raw.

**Male Participant:** They salt it. They kill it with salt when it’s raw. They just peel it.

**Male Participant:** I think what he’s saying is true. People wouldn’t like to take the raw fish soup. I think it’s not because of the herring. I think it’s because probably there’s no pepper-- enough pepper. When there’s pepper in it--

[crosstalk]

**Male Participant:** That’s another issue. I want to know something about the smoke you said. Sometimes, we roast. After it has been roasted, we smoke it. We smoke it as a way of preserving it. It becomes a little bit dry.

**Female Participant:** Drier.

**Male Participant:** Smoking, there are two things. We have smoking as preserving it. Very often, it’s not just even-- The fire is not very hot. It’s just the smoke coming from it. If it is coming, it’s put on there. You really blast it with the smoke so that animals and weevils cannot enter it. They also do the same thing with maize seeds in the north. They hang it there in the kitchen so that the smoke will go into it so that the insects cannot go into it.

**Male Participant:** You are confusing me. Roasting and smoking, that’s two different things.

**Male Participant:** That’s what I’m saying.

**Male Participant:** When you roast, you are eating it directly.

**Male Participant:** No, it’s not true.

**Male Participant:** You don’t roast. That’s what I understand. When you roast it, then you are eating it direct. Nobody roast and preserve it. We smoke it to preserve.

**Female Participant:** Yes.

[crosstalk]

**Male Participant:** There are two things. You roast and eat. There’s some left, you smoke it. That’s what I’m saying.

**Female Participant:** Yes, that’s all--

[crosstalk]

**Male Participant:** Okay. Now, we are getting into some technical things where I agree with **[unintelligible 00:59:30]** where I really don’t see the difference. Let’s drop it, because I don’t see the difference. In both case--

**Male Participant:** You don’t see it, but I see it.

[crosstalk]

**Male Participant:** I’m trying to be- [crosstalk] Did you get what I was trying talking about?

**Female Participant:** We will just say that we smoke it.

**Male Participant:** That’s what we call it, yes. Roasting and smoking, I see no difference, because in both cases you just put the meat-

**Female Participant:** On the fire.

**Male Participant:** You just put it on the fire. [crosstalk] There's another way, there's a difference, it's smoking the idea is to smoke it. For example I will move and give you an example. When we put our coolers on the **[unintelligible 01:00:16]** and put fire in it, we're smoking it, so that it will get into it so that the water will taste in a certain way and blah, blah, blah. It's the same way you can smoke meat to taste in a certain way but you can also smoke meat purely as a way of preserving it so that after it has been grilled you just boil because of you keep the same fire that you use-

[crosstalk]

[background noise]

**Male Participant:** Let's move on to the other.

**Moderator:** It’s all very interesting, it's all interesting to me.

**Male Participant:** Why are we talking about the health issues here?

**Moderator:** I was wondering if there was some bush meat that's more expensive than other meat and if like also how you see as a socio-economic status symbol, because last time we got together there was a discussion, I think I cant remember.

**Male Participant:** I understand the question.

**Moderator:** If there's a kind of meat that has a higher socio-economic status.

**Male Participant:** Example would be rat and grasscutter, who would feel more pride?

**Female Participant:** The person eating the grasscutter.

**Male Participant:** Why?

**Female Participant:** Because the rat is easily to get or catch or whatever whereas the process of the **[unintelligible 01:01:45]** is a bit more difficult so therefore the person that is serving it serves it for a bit more money.

**Male Participant:** I think monkey is also **[unintelligible 01:01:57]**

**Moderator:** Monkey?

**Male Participant:** Don’t say anything which is not true here.

[laughter]

**Male Participant:** Mpunam is monkey. It's a mixture of different bush meat. [crosstalk]

**Female Participant:** Bush meat is mpunam. [crosstalk]

**Male Participant:** No, mpunam is a mixture of various- [crosstalk]

**Male Participant:** No that is called, that is bobona.

**Female Participant:** No, it depends where you come from.

**Male Participant:** Maybe, hold on. [background noise]

**Male Participant:** Please don’t point your fingers at me I will get aggressive.

**Female Participant:** That's where you come from that's where they would call it bobona but it's all-[crosstalk]

**Male Participant:** Mpunam, so they're using some local terms here.

**Moderator:** I like it, I like it. I’m learning. If it's going very fast do you think I talk fast?

**Male Participant:** They're using some local terms.

**Female Participant:** It's not mpunam.

**Male Participant:** When you go to the market to buy, usually you don’t go there and say I'm going to to- Some people do but most people would go there and say, "Give me bush meat."

**Moderator:** It can be anything.

**Male Participant:** It can be anything. That bush meat is what some people refer to as mpunam. M-P-U [background noise] Some call it as bobonam if you hear those, so mpunam would be M-P-U-N-A-M and I’m seeing the other **[unintelligible 01:03:32]** is monkey area **[inaudible 01:03:34]**

[background noise]

**Male Participant:** If you live in Ghana you can tell.

**Female Participant:** Mpunam is mixture of- [crosstalk]

**Male Participant:** Bobonam **[unintelligible 01:03:44]**

**Male Participant:** He's not heard it as mpunam.

**Female Participant:** Mpunam is the mixture of bush meat. [crosstalk] Mpunam is a mixture of bush meat.

**Moderator:** Okay what is the name?

**Female Participant:** It can be antelope, it can be monkey, elephant.

**Male Participant:** No elephant.

**Female Participant:** Any bush meat.

**Male Participant:** You told me there was elephant in it.

**Moderator:** Elephant trunks maybe, that's the best part right? That’s what I read.

**Male Participant:** Do we eat elephant in Ghana?

**Female Participant:** We don't eat elephant in- [crosstalk]

**Male Participant:** In DA, they they eat elephant.

**Moderator:** The trunk.

**Male Participant:** In DA?

**Male Participant:** Yes.

**Male Participant:** Even in north **[unintelligible 01:04:24]** they eat anything.

**Male Participant:** Where will they get that elephant?

**Male Participant:** Are you sure? [background noise]

**Male Participant:** For forestry, in fact I am not talking about my experience with bush meat was actually from the 60s and the early 70s, in those days they even shot an elephant- [crosstalk] [background noise]

**Male Participant:** Sorry can we centralize, because otherwise it becomes a bit- [crosstalk]

**Female Participant:** This one bring me a monkey today, that one brings me an antelope today, this one brings me this today, after selling the chunks bit of it, the remains is what they chop them up the remains.

**Male Participant:** They mix it.

**Female Participant:** They mix it together then it becomes mpunam that's why it's a bit of.

**Moderator:** That is that a fancy dish, is that a fancy dish?

**Female Participant:** It's not, that again a rich person probably will not go and buy it.

**Moderator:** Okay, so that's what I was asking.

**Female Participant:** Because a rich person will go specifically and ask for those- [crosstalk] A rich person will go specifically to ask for what they want, "I want an antelope or I want a **[unintelligible 01:05:34]"**

**Female Participant:** I disagree because a rich man will say I want mpunam. Will tell the wife, "When you go buy [crosstalk] because he's selling mpunam the man doesn’t know the difference as far as his wife- [crosstalk]

**Male Participant:** Who tells you? [background noise]

**Female Participant:** Most men don’t know what they are talking about.

**Male Participant:** Not us.

**Female Participant:** You want you wife to put on the table so long as it tastes good.

[background noise]

**Male Participant:** Hold on, you will have your chance, the point here is and let's make it a bit orderly, so the point here is that a rich man will not tell the wife or the child or whoever is going to the market, "Go and buy me bush meat." The rich person will be specific and say, "Buy me grasscutter or antelope." That's what you're saying. Do you agree or disagree? Okay you disagree.

**Male Participant:** I totally disagree.

**Male Participant:** Why do you disagree?

**Male Participant:** Because the mpunam he is talking about is in my opinion a monkey.

**Male Participant:** How many agree with **[unintelligible 01:06:50]**?

[crosstalk]

**Male Participant:** I don’t agree.

**Male Participant:** Okay, hold on you have your point, the rich person will be more specific, or not? Would they say mpunam or mention the name of the meat?

**Female Participant:** I think the rich guy will say, "I want mpunam," because you have different- [crosstalk]

**Male Participant:** Variety.

**Female Participant:** -different varieties or different tastes whilst eating it.

**Male Participant:** No I don't agree.

**Female Participant:** Yes, a rich person will specify, "I want bush meat like a grasscutter or **[unintelligible 01:07:27]**

**Male Participant:** Who wouldn't want to specify, that's what they would want. It depends, let me say that something like a rabbit, we have the bush rabbit, I don't know what's their name. It doesn’t taste as good as a grasscutter for example, so if I had an opportunity to make a choice, I obviously, my rationale- but I would not go for rabbit, but would prefer- yes exactly.

**Male Participant:** You'll have your chance hold on.

**Male Participant:** Aside the, how do we call it? What you would want it may also depend on the time of the year. Some animals are more available at some times of the year than others are so it becomes some kind of demand and supply that determines which one goes up and so on and so forth, whereas some animals that are more available when the rains have just started so the the grass comes out, it's sprouting and they would want to eat it so they're easier to catch. When they're more available their price will usually come down but having said that, the taste also would also determine what somebody would what to go for, and they will come in and- [crosstalk]

**Male Participant:** Specify.

**Male Participant:** How would you say the dry season in relation to?

**Male Participant:** In the dry season, what I do know in the north is that they organize what they call hunting expeditions and then they go deep into the forest as groups with their dogs to go in and hunt. At that point, more rodents are available to catch and therefore the choice is not much because you don’t have one too many to choose from, so usually they would eat whatever that comes, but when they're many then you have a choice, then you can now make the choice. Later on, much later when we are concluding I will share some information that I have that has to do with my research work that has to do with bush animals and then infertility in men but that will be much later much later when we have.

**Male Participant:** Where do you stand? Rich man will be specific or will not order.

**Male Participant:** I cannot speak for many rich men, but I lived with a man who was relatively very rich, so I don’t know what other people do and I’m talking about the late '60s and the early '70s. I went to the market with my auntie and at the moment in fact every week I went to the market at least five times during the mid-day break to go and do the shopping, then bring it home put the **[unintelligible 01:10:13]** on the fire before I go to school again and I bought bobonam they used to call it also mpunam and what they did is they have some bones and they would put this meat on it, they will cut for you.

Looking back I think I have eaten some monkeys **[unintelligible 01:10:29]** and then they make a selection and they give it to you and my auntie and that's how she bought it and her husband was rich so saying that rich people will not go for the mpunam but they will go for specific, I do not know about that and I used to buy for many years for my auntie I used to do the shopping.

**Male Participant:** Here we are taking a general view.

**Male Participant:** I don’t know what other rich men will do but I think it's just a question of taste. There're rich men who will slaughter sheep for somebody, but they will only eat the entrails, so I don’t see the issue of richness with specific.

**Male Participant:** Here you are crossing the line.

**Male Participant:** No, I sided with you first part not because I agree with you, but I understood it, but the second part where you were eating certain parts, I think that’s a bit different because you're talking about the same animal. The point that you made is that because **Male Participant:** No, mpunam I would prefer mpunam.

**Female Participant:** Than?

**Male Participant:** Than a grasscutter.

there is status so someone will feel proud eating grasscutter than eating rat and I think that's true that many people will say, "I went to the **[unintelligible 01:11:51]**" when they ate rat, they wont go- [crosstalk]

**Female Participant:** If indeed they are eating rat they will say it's a **[unintelligible 01:11:55]**

**Male Participant:** Sometimes they will eat the wrong one. That's actually where you came from by saying people will be more specific because of the status attached but of course in certain cases you would have people just say, "Go and buy me mpunam." I know for a fact and of course I'm not speaking for the just speaking to the context that I know, that when people have money on them, they become more specific. Whey they don’t have money then- [crosstalk]

**Female Participant:** Anything goes.

**Male Participant:** Okay, can I ask the question we are two hours **[unintelligible 01:12:34]** at what time are we having this discussion because we're at two hours. I wanted to ask so that we can--

**Moderator:** What is convenient for everybody? Kwazi and everybody, at what time did we start the discussion?

**Male Participant:** We were here at one.

**Moderator:** Right what time did we start the discussion?

**Male Participant:** I’d say two o'clock.

**Moderator:** Okay so maybe another--

**Male Participant:** One hour should do.

**Moderator:** Yes, perfect.

**Male Participant:** Also, I think it's also a question that you cannot answer it on your own. If we also make the discussion more focused we can get more out of it because sometimes it goes everywhere and I understand- no, no, no I think it's a point that I want to make because I can understand your sensitivity to interrupt which some of us can better do so let's also keep it a bit focused and discuss the items.

**Male Participant:** I want to make a statement. I'm having the feeling that there's amount of shyness when I talk about monkey.

[laughter]

**Male Participant:** No we will talk about it and I will explain something to you which you don’t know.

**Male Participant:** Allow me and that feeling I think and there was a statement which you made that when animal is sick he dies, no when I go to the farm and I see a dead bush meat- [crosstalk]

**Female Participant:** They bring it home.

**Male Participant:** -bring it home.

**Male Participant:** The first point is that shyness, no hold on there's shyness about asserting that you eat monkey.

**Female Participant:** That's what I said I've eaten bush monkey before, but I've never eaten the one in the house before. Again, the bush monkey they call it okokwo.

**Male Participant:** Or shrobwa, that's the kept one.

**Female Participant:** The one that comes home is abe, so it's different names. [laughs]

**Male Participant:** Do you feel shy, and maybe I can go first because I think that I agree with the point that you made. There're different species they may be the same animal species, but they will have different rates in the same way that sakraman is the counterpart of dog in the bush.

**Male Participant:** Bush dog.

**Male Participant:** What is sakraman in English?

**Male Participant:** Fox.

**Male Participant:** No, but it is not even fox because, yes bush dog, I think wild dog.

**Male Participant:** Africa wild dog, there are different types.

**Male Participant:** Yes they're the same from the same species, but they're different, so I won't go and say that I've eaten dog when someone brings that bush dog to me, so when it comes to- [crosstalk]

**Male Participant:** You say bush dog.

**Male Participant:** No, no, no hold on. I won’t say I have eaten dog and that's the mistake **[unintelligible 01:15:36]** is making by saying that monkey the one in the bush we don’t call it monkey, no we call it- [crosstalk]

**Male Participant:** In English.

**Male Participant:** -adow so A-D-O-W is the one we have at home.

**Moderator:** A-D.

**Male Participant:** It is sort of domestic and the one we have in the bush has a different name it’s okokwo.

**Male Participant:** Ashrobwa.

**Male Participant:** Yes, so there're two different species so it's wrong to say that I've eaten adow maybe let me use the Ghanaian terminology then it becomes a bit more clear.

**Moderator:** That's clear, that's very clear.

**Male Participant:** Nobody in Ghana almost everyone in Ghana will deny eating adow, but if you say okokwo most people will say yes because they're talking about different animals and I think that's what we have to get right.

**Moderator:** Okokwo is the monkey? This is very helpful.

**Male Participant:** You want to ask a question first?

**Moderator:** Yes, this is so helpful to me because it is confusing, I don’t know anything so I’m trying to get the terminology it’s helpful, so with the okokwo that is what kind of species? [background noise]

**Male Participant:** That's exactly the issues here. There are a lot of species even when you comes to this okokwo and in English, the baboons. The big ones.

**Male Participant:** To the smaller ones.

**Male Participant:** It’s a very big--

**Moderator:** Range.

**Male Participant:** Exactly but as far as the Ghanaian is concerned, anything there is okokwo despite the fact that there is a variety there because just as you're saying I haven't eaten monkey before because if the person says I've been eating monkey, he or she is talking about the domestic ones, the small, small ones in the house.

**Male Participant:** Like the pet- [crosstalk]

**Male Participant:** I say okokwo **[unintelligible 01:17:57]** so they appreciate that so that is the difference. It's exactly the difference between a mouse and a rat. I can bet you somebody will catch a mouse and eat it but then you would say no **[unintelligible 01:18:18]** When it comes to status, then somebody will say, "I don’t eat **[unintelligible 01:18:26]** I eat **[unintelligible 01:18:26]**" It's a sort of hierarchy thing going up and down- [crosstalk]

**Moderator:** Up the food chain.

**Male Participant:** Up food chain, it's like in Nigeria I will say that speak for senior officers. The rich people in Nigeria eat snakes, it's their delicacy, yes. It is their delicacy and the rich people buy it.

**Male Participant:** It's also bush meat. You see the difference if you want to contest the relationship and the status.

**Moderator:** This might be a silly question, but you'll have to excuse me because I don’t know. The bigger the monkey, the more money you have to spend?

**Male Participant:** I won’t say the bigger the monkey, I think the species.

**Female Participant:** That’s what goes house one is a no-no but the bush one it doesn’t matter whether it's small one or big one or whatever so long as it comes from the bush.

**Moderator:** All of the monkeys from the bush have the same price?

**Female Participant:** Same price, they're more expensive than the one - [crosstalk]

**Male Participant:** Okokwo is okokwo full stop.

**Male Participant:** Let me make a statement. When it comes to domestication of animals, what I have learnt, at least my perspective is that, when the animals try to come to the vicinity of our homes, they become more difficult for the Ghanaian to eat. I can give an example, you can have even a sheep, a sheep is an eating thing but when sheep becomes attached to human beings, like my mother had a sheep, it will be just walking everywhere, people find it very difficult, because it's become near.

That thing us why we explain what we call okokwo, I've heard people saying it. When okokwo comes home, when a monkey comes home, they call it adweny. I know people who have been able to capture a monkey from the bush, is able to bring home, and then when it comes to that stage, people say, "Hey, this is-" I want to make it categorical that in my opinion, it's the issue of domestication which makes people think that we don't eat this, and we don't eat that.

**Moderator:** I understand that very well because in Singapore, we had a dog, and our neighbor had a dog, a pet dog, he was Malaysian and he ate dogs, but he didn't eat his own dog. In Malaysia. I think I understand, it's just that I don't understand the pricing. If you want to go and buy a baboon, is that going to be the same as a small monkey? Is the status the same if it comes from the bush when you make a dish with meat with baboon, or you make a dish with the meat of a smaller monkey? This is just one example.

**Male Participant:** How do they determine the price? I think it's the energy which is put in getting the animal from the forest. The danger, the availability, the season, and the number of people who eat it.

**Moderator:** Depending on how endangered it is they build--

**Male Participant:** Not endangered it is.

**Moderator:** How much danger it takes to get it.

**Male Participant:** The skill needed to catch it, the availability, because maybe in a certain season you can kill two a week, but in other seasons, only one a month.

[crosstalk]

**Moderator:** Like you were saying, it's about supply and demand.

**Male Participant:** I think it's a very good question which I haven't really thought a lot about. What determines the price of bush meat? If we make it more, let's take three species. Grasscutter of course--

[crosstalk]

**Male Participant:** Grasscutter from Accra to Amsterdam?

**Male Participant:** No, just talk about Ghana.

**Male Participant:** Locally.

**Male Participant:** Yes, so we have grasscutter, then we have squirrel, and let's say typical rats.

**Male Participant:** Add snails.

**Male Participant:** Is snail a bush meat?

**Male Participant:** Snail is a mollusc.

**Male Participant:** Take antelope.

**Male Participant:** Yes antelope, because I think they are quite expensive. When we talk about rat, everybody agrees it's cheap because people feel when you eat it, your status is low, and it is available. The availability is okay. Then you go to grasscutter and squirrel, which will be more expensive per kilo?

**Male Participant:** The grasscutter.

**Male Participant:** Squirrel is more expensive. Squirrel is very difficult to catch.

**Male Participant:** People don't like it.

**Male Participant:** Who and who?

[laughter]

**Male Participant:** You and me.

**Male Participant:** People like squirrels. There's also another thing, squirrels, there's a myth about them. Their tails are used in certain rituals to disappear. They say when you're coming, and the squirrel taps it, it's gone. They use it for people who want medicine to be able to **[unintelligible 01:24:41]** It makes the price of squirrels--

**Female Participant:** I think they don't even sell it. [crosstalk] I don't think they sell it.

**Male Participant:** If they are selling, which will be more expensive? [crosstalk] That is my point, the squirrel or grasscutter?

**Female Participant:** I think maybe the squirrel because it's very, very difficult to get it.

**Male Participant:** What do you think, which will be more expensive?

**Female Participant:** I don't know much about the selling price.

**Male Participant:** You don't have an opinion about grasscutter?

**Male Participant:** Squirrels are not expensive in the Northern part of Ghana, just the other parts. They are burnt, they burn squirrel.

**Female Participant:** We have bush cat.

**Male Participant:** Let's just talk about these two because we're trying to determine the price.

**Female Participant:** Okay.

**Male Participant:** They will have a tie, because I think, this is my perspective and you can disagree. I think if you take it kilo, the squirrel will be more expensive, because it's difficult to get. Some people, at least where I come from, there may be regional differences are also important, because in the North you can get them everywhere, so maybe in the north--

**Male Participant:** Squirrel is cheaper than grasscutter.

**Male Participant:** Yes, whereas where I come from, it would be the other way round, because some people have also created this myth about meat of the squirrel and what it would do.

**Moderator:** You're from up in the mountains?

**Male Participant:** Yes, I'm from the mountains.

**Male Participant:** Can I make a statement? I think I do still not agree with any of you guys. Squirrels meat on the market, probably going to buy them to make medicines, for the dry ones, but to eat, to go and really buy meat on the market, looking for squirrel meat--

[crosstalk]

**Male Participant:** I think that's important.

**Male Participant:** Allow me. Something like grasscutter is a norm, it's something people go and buy. The comparison for me--

**Male Participant:** The point that Hamsin made about medicines is true, because grasscutter has no medicinal value. One squirrel, people believe that you can use it to tame a beast.

[crosstalk]

**Male Participant:** I agree, but to come back to what Jake was saying, supply and demand. Now, in the market, people want their money quick so they'll go in for Accra ntiers. If during the hunting for the accra ntier, he or she get the chance to get oprom be somewhere, it is a bonus. They don't necessarily go there to be hunting for the squirrel, it's just an extra which by chance by going to hunt for grasscutter, you get it. That is for your soup at home, not for the market.

**Male Participant:** I think for the sake of time, you can also explore this question further with the next group.

**Moderator:** I have a question. The price of one kilo of punam, hope I am saying that right, how much more expensive would it be if you have to buy it here in another land?

**Male Participant:** That would be very difficult to calculate because you have to think about how much it costs in Ghana.

**Male Participant:** No, make it simpler. [crosstalk] You go to the market, and then you buy a kilo grasscutter for a certain amount of money. When you go to the shop here for the same kilo?

**Male Participant:** You'll pay more.

**Male Participant:** How much more do you pay?

**Female Participant:** It's like €25 or something.

**Moderator:** €25 for one kilo?

**Female Participant:** Yes.

**Moderator:** How much in the market in Ghana for one kilo?

[crosstalk]

**Female Participant:** It's about ₵12.

**Male Participant:** ₵12 will be about €2.

[crosstalk]

**Female Participant:** I think it will be about 60-65% more here.

**Male Participant:** No, more than that. More than 60%, because one [foreign language] by the roadside could be let's say ₵30, ₵40, a whole akrantie. The whole akrantie itself, it wouldn't be two kilos. If two kilos is ₵30 in Ghana. Some of them are heavy.

**Female Participant:** Some are heavy.

**Male Participant:** Let’s just average is two and a half kilos and you’ve got ₵30, ₵30 is about €4.

**Male Participant:** Yes, for example. It will be more than ₵30.

**Female Participant:** It is more than ₵30. It’s hundreds.

**Male Participant:** Okay.

[crosstalk]

**Male Participant:** The last time I checked was very long time.

**Male Participant:** It’s in the hundreds.

**Female Participant:** It’s in the hundreds.

**Male Participant:** It will be 100, yes. 100 will make it €20. 100 will make the whole akrantie which is two and a half kilos about-- Averagely, that will be €20.

**Female Participant:** Which you cannot afford the whole one here.

**Male Participant:** Yes. It is about--

**Female Participant:** That’s why I’m saying it’s about--

**Male Participant:** 200%.

**Female Participant:** Yes. 60% more-

**Male Participant:** 200%, because--

**Female Participant:** - because you cannot afford it.

[crosstalk]

**Male Participant:** Here, there they will divide it into four and then give you part for that--

**Male Participant:** For €25.

**Female Participant:** Don’t you buy it in kilo?

**Female Participant:** Here, you will buy it in grams. You cannot afford to buy--

[crosstalk]

**Male Participant:** What I sometimes buy is fish.

**Female Participant:** The dry fish.

**Male Participant:** The dry fish in Ghana maybe you will buy a bunch for ₵10. ₵10 will be €2. When you come here, the whole one you will buy-- When you go to the Suriname shop, it will be--

**Female Participant:** He went to a Ghanaian shop.

**Male Participant:** Yes, you can buy it around ₵10.

**Male Participant:** Two of those small ones?

**Male Participant:** Yes, €10.

**Male Participant:** €10? The woman said how much?

**Female Participant:** 15.

**Male Participant:** I’m talking about the one **[unintelligible 01:31:42]**.

**Male Participant:** Okay.

**Male Participant:** Then you will have a difference like 200%. So then the question will be, "Are the people here making all the profit?" I don’t think so.

**Male Participant:** No. I don’t think so.

**Female Participant:** It is how to get it here.

**Male Participant:** Yes. It is more about what you get and the cost that you make in Ghana when you bring it here and the storage and the fact that sometimes some will go bad and all those things.

**Male Participant:** I will add a point. This is a part of your research that you can easily do. You go and buy, ask how much they sell it here and then compare it with the price in Ghana. That’s very easy.

**Female Participant:** The reason why it’s more expensive--

**Male Participant:** If it goes to the shop, they won’t sell it €2.

**Male Participant:** No.

[laughter]

[crosstalk]

**Female Participant:** It is more expensive. It’s the transportation, the duty. It’s everything that involve in bringing the thing here.

**Male Participant:** I will hardly see people who really make grasscutter soup here.

**Female Participant:** We don’t get it here.

**Male Participant:** You can’t this small just to make your--

**Female Participant:** That’s what it is.

**Male Participant:** You know. You buy a whole like you did in Ghana.

[crosstalk]

**Female Participant:** We buy it in grams.

**Male Participant:** You got to change the place.

**Female Participant:** Now, we don’t get it because it’s banned now.

**Male Participant:** Can we--

[crosstalk]

**Moderator:** How do trust it? Back home, you go to your hunter. You go to--

**Male Participant:** The market.

**Moderator:** How do you trust--

**Female Participant:** When it gets to Europe, it’s because you are earning for it. Then you don’t care where it comes from.

**Male Participant:** You yearn for it.

**Female Participant:** Yes, because as long as you can get your hands on it.

**Moderator:** You want it because it reminds you of home. You want it because it tastes better.

**Female Participant:** It reminds you of home.

**Male Participant:** It makes the soup smell better [laughs].

**Female Participant:** It has nothing to being home.

**Moderator:** The connection back home and it tastes better. It tastes also better?

**Male Participant:** It makes the soup smell great.

**Female Participant:** Smell better.

**Male Participant:** If anybody will buy akrantie here being 10 grams or a kilo, it is pure the prestige.

**Female Participant:** It’s just to reminding you--

**Male Participant:** It’s just a prestige of I buy akrantie here and Adansi comes, "Kwame, is that akrantie that you’ve bought?" Yes, it’s just prestige. That is all. It is nice sharing it. I don’t think Koo Boy would like to--

[crosstalk]

**Female Participant:** I can afford steak today so that you eat it every day. After that, you will eat it every day. All the days when you’ve got a little bit extra. It’s just like you want to shoot yourself, because compared to other meat it is expensive. Compared to other meat, it is expensive. The process that they go through to bring everything, it makes it a little bit expensive. Whereas in the villages, nothing. In the city, it becomes expensive.

**Moderator:** You know where it’s coming from when you’re back in Ghana and you’re a little less--

**Female Participant:** No.

**Male Participant:** Here, I won’t eat it. For me--

**Female Participant:** Even in Ghana you won’t know where your-- Adansi says when he goes to his village he knows who he is buying it from. If you buy it in Accra or the marketplace, it is a little bit expensive.

**Male Participant:** The smoke ones, some of them are so old.

**Female Participant:** You will get some of them. They will say that, "Nice one."

[crosstalk]

**Male Participant:** Then that’s why they smoke it, and then they sell it here.

[crosstalk]

**Female Participant:** He only shoots. He doesn’t put medicine. He doesn’t do **[unintelligible 01:35:42]**. The salesperson wants their money. They will tell you anything to get their money.

**Male Participant:** That one it doesn’t matter if you buy it here. You don’t know how they got it.

**Male Participant:** Also, we used to import them ourselves when we go and buy them and bring the meat.

[crosstalk]

**Female Participant:** You don’t know where they are buying it from.

**Moderator:** Now, my understanding is that you will only get it and take it away. You don’t get a fine when--

**Female Participant:** Is it?

**Male Participant:** They will fine you. You are not supposed to bring it.

**Male Participant:** They will confiscate it and destroy it.

**Moderator: [unintelligible 01:36:18]**

**Female Participant:** Then in that case one will try and take your chance.

**Male Participant:** I think they will give you a fine.

**Female Participant:** I think they will give you a fine.

**Male Participant:** They will give you a fine, because in Holland they used to just warn. They will give you a fine, because you are endangering public health. They will tell you in Holland you are bringing Ebola here.

**Female Participant:** Only fish that you can bring.

**Male Participant:** They will give you a big fine.

**Male Participant:** Here, they will give you a fine.

**Female Participant:** I think the only way that maybe you will get away with just-- taking off you is if you’ve got a health certificate from Ghana with it. Even in Ghana when you are coming and you went for the health certificate, they would ask you which country you’re taking it to. They will tell you whether you’re allowed or you’re not allowed, because I myself

every time I’m coming from Ghana, anything that I will bring from Ghana, any foodstuff, I will make sure that I’ve got a certificate with it even if it’s just gari which is--

**Male Participant:** Have you ever gotten a certificate for bushmeat in Ghana?

**Female Participant:** Never.

**Male Participant:** Okay. They won’t give it to you.

[crosstalk]

**Female Participant:** Because any time I do bring it, I don’t tell them.

**Male Participant:** In Ghana, they won’t give it to you.

**Male Participant:** I know that bushmeat finds its way out into this country. [crosstalk] My neighbors have a party almost every summer.

**Male Participant:** With bushmeat?

**Male Participant:** I tell you.

**Male Participant:** Next year, they will arrest them because of--

**Male Participant:** No, they will not arrest them. They are from Ivory Coast. They know how to [laughs]. I didn’t know that their eating culture is different from us. I don’t know. The Ivoirians, they eat a lot of meat.

**Male Participant:** Yes, the French speaking countries.

**Male Participant:** How did you know? [crosstalk] It’s all these bushmeat, a lot of them when they are chewing it and they are drinking.

**Moderator:** You were, saying if there’s a demand there will always be a supply. I know that in France we eat things that people in America do not like to have like foie gras or something like that.

**Male Participant:** Yes, foie gras. [crosstalk]

**Moderator:** I grew up eating this, and I love eating this. My friends in America really could not understand it, and then it’s not sold.

**Male Participant:** Do you eat frogs?

**Moderator:** No.

**Male Participant:** It is duck and--

**Moderator:** It’s not very nice.

**Male Participant:** Is it liver? They squeeze it. They force them to eat a lot. They have swollen liver, and then they get--

**Male Participant:** **[unintelligible 01:38:44]** ducks?

[crosstalk]

**Male Participant:** Then they prepare them. They love it.

[crosstalk]

**Male Participant:** Even frogs. They eat frogs.

**Male Participant:** Foie gras, yes.

**Moderator:** Horses?

**Male Participant:** I think we should start eating horses in Ghana- [crosstalk] - so that we don’t import the chicken from Europe.

**Moderator:** I guess my point is that your point and the last two session that we spoke, also, we spoke about the fact that any time people want the meat they will find a way to get the meat.

**Male Participant:** Absolutely.

**Moderator:** If the meat is here. It’s just me trying to understand. Does it taste the same when you eat bushmeat here as it taste when you’re home? Or does it taste different?

**Male Participant:** It’s the same.

**Male Participant:** I wouldn’t say it taste different.

**Male Participant:** I think it’s a good question.

[crosstalk]

**Kwasi:** The bushmeat that we have in Ghana which is sold on the market are the same bushmeat you see alongside the road. They buy it and they just go and market it. It is the same Bush meat that they buy from the market and bring it here. Taste wise, there’s no difference. Just that it's expensive.

**Moderator:** Okay, the people that are selling it and the butchers, they are getting it from the same source?

**Kwasi:** Kwame, Bush meat without soup is-- The soup determines the taste of the bush meat.

**Kwame:** The bush meat determine the taste of the soup.

**Kwasi:** If they just give you the bush meat raw, I will agree with what you’re saying. Normally, they make it with soup. I think in Ghana the women can make or the men-- Some of us, we can make-- These guys they cannot prepare soup. That’s why they are saying that it isn’t the same.

[laughter]

**Kwame:** No.

**Kwasi:** There are some of us who can prepare soup.

**Kwame:** Some of who?

[laughter]

**Kwasi:** They know that it is the soup that makes the difference.

[crosstalk]

It goes even further. It’s a woman’s hand which makes the fingers.

[crosstalk]

**Kwame:** No. Kwasi, I will challenge you to this and tell you that if all of us are given the same ingredients to prepare food, there will always be a difference, whether you like it or not.

**Male Participant:** No, let’s say soup.

**Kwasi:** Don’t talk about soup.

**Kwame:** Even soup--

**Kwasi:** Akrantie soup.

**Kwame:** Even akrantie soup, there’s always going to be difference. When I say that the taste is the same, I’m talking about the general taste. That it is no different from how you prepare it in Ghana, because if you prepare bush meat in Ghana with your soup and you eat it, it tastes good. You this same person if you are giving the same ingredients here, it’s going to taste exactly the same thing to you. You see, it doesn’t mean that I like your soup.

**Male Participant:** Your soup, probably, the taste will be quite--

**Kwasi:** So then the point is that bush meat soup in Ghana tastes better than bush meat soup in Holland?

**Kwame:** No. That’s not what I’m saying.

**Kwasi:** He said, “You get a quarter bush meat, akrantie, you make soup there, and you eat it. And then you come to Holland, you find the ingredients. They bring you a quarter bush meat and you cook it again, the taste will be different-

**Kwame:** Why?

**Kwasi:** - here.”

**Kwame:** Why?

**Kwasi:** Koo Bro, everything is different. You cannot step into the same river twice.

**Male Participant:** No. Even it's the same person the taste will be different, because you are using vegetables which in most cases are different.

**Kwame:** Okay, let us assume you are using exactly the same vegetables.

**Moderator:** Maybe this is what you were talking about before which is, “Is it the soup that determines the taste, or the meat that determines--

**Female Participant:** The meat.

**Moderator:** Okay, you think it’s the meat that determines?

**Female Participant:** It’s the meat that determines the taste.

**Kwasi:** Heraclitus says that you cannot step in the same river twice.

**Male Participant:** Who is that?

**Kwasi:** He’s an ancient philosopher. A great philosopher. What he meant was that if you step into the water now and take it off your feet, the water is moving. If you step in it, you are not stepping in the same water.

If you cook today with all ingredients same thing here, and then you eat it. Two hours later you cook, it will never be the same. The performance will not be same.

**Kwame:** Exactly.

**Kwasi:** In principle, you cannot answer the question if you cannot generalize.

**Kwame:** Exactly. That’s my point.

**Kwasi:** In principle, it is the same.

**Male Participant:** I think you are making the answering of the question more complicated, because--

[crosstalk]

**Female Participant:** I think also the meat, the taste I think there’s difference in the taste. Some taste very nice. Some also doesn’t taste very good.

**Male Participant:** Because they have travelled to here?

**Female Participant:** No, it’s not like that. Let’s say chicken. Even chicken, you can buy chicken. If you buy two different kinds of chicken, and using it to prepare soup, even if you are tasting it, you will taste difference. Some will taste good.

**Kwame:** I agree.

**Female Participant:** The other one will taste--

[crosstalk]

**Kwame:** Because they don't come from the same parent.

**Female Participant:** Yes. That will determine how the soup will taste.

**Kwasi:** Chicken from the same parent.

**Kwame:** They are not from the same parent. The bush meat--

**Kwasi:** Kwame.

**Kwame:** The bush meat on the road, they are not from the same parent.

**Female Participant:** No. One will taste good.

[crosstalk]

**Kwame:** There are likelihood that will--

[crosstalk]

I agree.

**Male Participant:** Are we not agreeing that if you go to Ghana and you take the soup there, you feel it’s better than here?

**Kwame:** No. It’s exactly the same.

**Male Participant:** If you eat kenkey in Ghana and the kenkey you eat in Amsterdam, are they the same?

**Kwame:** They are different.

**Female Participant:** That one is--

**Kwasi:** The way of preparing it--

**Kwame:** With the first question you asked, it could be different.

**Male Participant:** Let’s say, for example, it takes a long time to travel here.

**Female Participant:** Yes, that’s the thing.

**Male Participant:** It could be different. Having said that, I think I agree with you. Generally, it is the same.

**Kwasi:** It’s the same.

**Male Participant:** When we say generally, it does not mean that there isn’t some level of variation.

**Kwame:** Exactly.

**Male Participant:** What I’m trying to say is that the level of variation is quite insignificant.

**Kwasi:** Exactly.

**Female Participant:** The processes they are the same, but the taste is different.

[crosstalk]

**Kwame:** The soup I eat in Ghana-

**Kwasi:** You prepare it yourself?

**Kwame:** They taste better. The tomatoes taste different. The onions are different.

**Kwasi:** I’m not just talking about the soup. I’m talking about the meat and all those things. If I go to a restaurant in Ghana, don’t talk about the one I prepared. I go to a restaurant and then I will ask for-- I don’t eat meat. I cannot use--

**Female Participant:** Fish?

**Kwasi:** Yes. I will take fish as an example, or light soup. What you have in Ghana if you try it 10 times it will be better than what you will try here 10 times. It’s just not coincidence.

**Kwame:** No. You see, this is exactly my point which I made in the first instance when we were talking about the kenkey. I said that maybe the flies play their role. When you go to the chop bar--

**Kwasi:** The fly is a representative of that honorable--

**Male Participant:** No. You are making an important question a bit trivial.

**Kwasi:** No.

[crosstalk]

**Kwame:** You see, it comes back to what I’m saying that--

**Male Participant:** The flies thing, **[unintelligible 01:47:07]**

**Kwasi:** No. Let’s see the fly as a personification of all those factors, which you cannot--

**Male Participant:** Kwasi, when you prepare food, if you go to a restaurant with all the ingredients- I'm coming back to what Dios is saying- you prepare it with akrantie or whatever, with fish, you even do it yourself. As you said, after a day you go and prepare it again, and there might be a difference, but that difference comes back to what you said. If we have to generalize it, it’s nothing. It’s the same.

**Kwasi:** Point well taken.

**Male Participant:** Unless we go into the specifics that is where the difference-- Because when you started the first meal or the dish you said, “Okay, let me take X amount of salt.” The next day you said, “It wasn’t much. Let me take-- It changes the taste.

**Kwasi:** Is there another question?

**Moderator:** I do, because Kwasi and this nice gentleman, Jacob, you mentioned how the meat can change possibly in taste when it travels. Is it your understanding that it’s frozen and then defrosted? Is it smoked and then you don’t have to worry about the change-- As much variation in the change and taste, because it’s smoked? The smoked flavor stays the same for a longer amount of time. Then there is fresh meat that is sold here at the butcher, and then that actually you think cannot travel raw. It will have to be frozen and defrosted. The smoked meat sounds like it’s the one that’s the most flavorful. That would be easier to transport, I would imagine. Then you could still have the same flavor. Flavor would not be as affected as dishes that it involve meat that the recipe requires raw meat.

**Kwasi:** How did they bring it here?

[crosstalk]

**Male Participant:** This is not a woman issue.

**Male Participant:** What I was going to say is, the question is a little difficult to answer, because you have to look at it from various forms. First of all, even if it is smoked, is it over smoked? Is it under smoked? Is it too dry? How long does it take to come here? These are some of the factors- Even in Ghana, if we were in Ghana, and one is over smoked, you could over smoke to the extent that parts are even burnt, such that when they eat meat, me for one, I can test the burnt part, it’s slightly bitter for me. I don't enjoy it. Let us say that if we had a machine, to calibrate the smoking and just smoke at a certain unit, same was transported here, I think that it’s to be the same taste, nothing burnt. However, let me also say that psychology, once mind also plays a role. Ones I get home to Ghana, I have this relaxed mind and I am really there-

**Male Participant:** For the real thing.

**Male Participant:** -for the real thing, and therefore is going to taste the way the oxen taste. Then when it comes here so well you know is gone through some process, some journies and so on and so forth. Psychology, mentally you have this thinking, believe that informs your taste.

**Moderator:** Yes, it does.

**Male Participant:** It does inform your taste that there’s a slight variation in taste, otherwise, I think that it’s not very easy to see.

**Male Participant:** I want to tell you something, because we're talking about-- She was asking in the transportation, how is it transported? Those are specific questions that’s why I said we should ask her because those of us or those-

**Male Participant:** But she doesn’t eat bush meat so-

**Male Participant:** You don’t eat bush meat?

**Male Participant:** Why should we ask her?

[laughter]

**Male Participant:** No, no the day that you do the shopping, or maybe you. [crosstalk]

**Female Participant:** Can I say something.

**Male Participant:** Just a moment. I asked because he has just come and he doesn’t buy those things. Those who are here, who buy akrantie will know whether is taken out of the freezer, because sometimes after they smoke, they store them in the freezer.

**Male Participant:** They don’t ask. They go there and they say that, "Do you have akrantie?" They said, "Yes or no or yes, how much? Let me weight it for you 20 kilos.

**Male Participant:** And they don’t take it from the freezer.

**Female Participant:** Can I say something-

**Male Moderator:** Yes, sure.

**Male Participant:** -that- let me tell you something.

**Male Moderator:** You can hold on, hold on.

**Male Participant:** Let me continue. We are talking about whether they take it from the freezer, yes or no. The selling or the marketing of akrantie here in Netherlands or here in Amsterdam actually is illegal, it’s not a public thing. If you have to ask whoever is selling is okay.

**Male Participant:** Under the table?

**Male Participant:** He say okay, come next week I'll get one for you, so you're not going to ask where it’s coming from.

**Female Participant:** Exactly what they say in there. People place orders. They know where to get them. They go and place orders before even the akrantie or the grass cutter arrives.

**Male Moderator:** You cannot buy-

**Female Participant:** No, whoever is bringing it from Ghana, they know where to take them off from the market. When you pick it they add it to certain food stuffs straight to them. These days come by air. They go straight to the airport, they fly them, in the next two days is out from the airport to wherever from the cargo section is out. They start calling people to come and pick it up. Trust me, it doesn’t take three days. Yes, it goes very fast for people place orders. "Do you have it? oh, okay, write down my telephone number, this is the quantity I need." Then they will be writing it down. The moment is, "Oh, yes, the grass cutter is in, my shipment is in so, whatever you need." It goes very fast. It doesn’t even [crosstalk]

**Moderator:** I didn’t know that.

**Female Participant:** Now you know how that goes.

**Moderator:** There’s only a few ways that it can get here, but that’s different then if it’s coming by boat, which takes so much longer.

**Male Participant:** No, its earlier than that.

**Male Participant:** I don’t think it will come by boat.

[crosstalk]

**Female Participant:** Before when it was allowed.

**Male Participant:** It was coming from **[unintelligible 01:53:53]** Antwerp is the main source of akrantie.

**Male Participant:** But you have to freeze it, right?

**Female Participant:** No, before when it was coming, when it was allowed but now it’s not so even in one of those days.

**Male Participant:** They smoke it very dry.

**Moderator:** So maybe it’s not coming through the ports anymore.

**Male Participant:** They smoke it very dry and then even then the guy who- the main importer of the name food stuffs, I’m not supposed to say his name.

**Male Moderator:** Okay, don’t mention the name.

**Moderator:** Yes, that’s okay. I don’t need to know any names of anything. Its really not the point. The point is to understand how the taste can change, how you feel how the taste changes, or it doesn’t change.

**Male Participant:** I think it’s a very complicated question when it comes to the issue of taste, because taste has something to do how much moisture is in it, how much fat is in it, what are the animals eating, all these things come into it, and even the smoking process influences. In Ghana now, people are rearing grass cuts. Those reared are different from those cuts in the bush.

[crosstalk]

**Male Participant:** That’s true but you can also simplify it and say under the same circumstances would that grass cutter, taking into consideration the fact that it was spend about three weeks here in the boat, and that’s the only thing that changes and the moisture. Would the taste be the same? If they were sisters and brothers and cut at the same time, and one is sold in Ghana and one is transported to Angola.

**Male Participant:** I think when you say that when is coming from- I would say when its coming from- if I bring it from **[unintelligible 01:55:46]** it won’t change.

**Male Participant:** So generally, it won’t change?

**Male Participant:** If I get it from Ghana it won’t change but I would take a difference when I come to **[unintelligible 01:55:57]** [crosstalk]

**Male Participant:** Let’s take it from your case.

[crosstalk]

**Male Moderator:** Let’s move on.

**Moderator:** If its smoked all the way through like that, then similarly to what madam was saying, the idea is that the impression that people have is that when you smoke all the way through, you've killed any possible-

**Male Participant:** Germs.

**Moderator:** -germs. That’s what you're saying.

**Male Participant:** The chemicals, that’s why I don’t take grass cutter now, because if it’s been killed-- I remember traveling along from monkey scene to Accra and buying the grass cutter. The last grass cutter I ate, and I believe it was killed with a poison. If it travels poison don’t change whether you eat them or not.

**Moderator:** That’s not germs, those are chemicals.

**Male Participant:** Some of them they use DDT, right?

**Male Participant:** I don’t know **[unintelligible 01:57:10]** there’s a chemical they use, the one-

**Male Participant:** There's one used to preserve bodies.

**Moderator:** What?

**Male Participant:** One used for preserving dead bodies.

**Male Participant:** Yes.

**Male Participant:** Formalin

**Moderator:** Formaldehyde?

[crosstalk]

**Male Participant:** They use it for preservation.

**Moderator:** They use formaldehyde, they use [crosstalk]

**Male Participant:** To preserve cold meat **[unintelligible 01:57:29]**

**Male Moderator:** Are you sure?

**Male Participant:** Yes sure.

**Male Participant:** It was in the papers. They caught some people who had used it. Not on akrantie but on **[unintelligible 01:57:36].**

**Male Participant:** Then we shouldn’t make a general statement. If a criminal uses it-

[crosstalk]

**Male Participant:** If a criminal uses it, we shouldn’t make a statement like they use it, because-

**Male Moderator:** It’s the criminal.

**Male Participant:** -yes, it’s a criminal thing, so that shouldn’t part of the discussion.

**Moderator:** Outside maybe of the norm.

**Male Participant:** Yes.

**Moderator:** Sort of an outlier?

**Male Participant:** Yes. The last time I had meat to akrantie **[unintelligible 01:58:04]** was in **[inaudible 01:58:05]** in 1986, and I had enteritis after that. I was hospitalized for five days at Lagon hospital. That was my last akrantie.

**Male Participant:** You ate?

**Male Participant:** I- yes.

**Male Participant:** You should stop eating meat.

**Male Participant:** I only eat chicken and fish.

**Moderator:** How can they poison the meat and expect that the customers will come back?

**Male Participant:** They don’t expect you to come back, they expect to have customers, others will come. If you don’t come others will come.

**Male Participant:** Is not a matter of poisoning it, and this is quite sensitive. I think we have to handle it with care. Some years ago, DDT was allowed in this country to spray vegetables and all those things, and they were not poisoning it. It’s something that you use to prevent something.

**Moderator:** So it’s not the dose. I mean it’s not the poison, it’s the dose?

**Male Participant:** Yes.

**Male Participant:** And awareness.

**Male Participant:** Even themselves they do not know that they were poisoning. They know they're killing it for us to eat. They don’t know it was **[unintelligible 01:59:13]**

**Male Participant:** All right, please lets go on, because we **[unintelligible 01:59:19]**

**Moderator:** Do you have any question?

**Male Moderator:** When was the last time that somebody ate bush meat?

**Moderator:** I have some questionnaires. Don’t put your name on it, doesn’t matter, that’s not important at all. My daughter put water on it. I was like **[unintelligible 01:59:44]** mom has to go now," and then she knocked over my glass, but it was much neater. Its questions and then you can just write it so that I can then organize it. in an organized manner in a spreadsheet. It's anonymous, so there's no name on it, and then it's 10 minutes and then we're done. Is that okay?

**Male speaker:** Yes.

**Moderator:** Yes, because it has a question like last time you ate bush meat, or how many times-

**Male Participant:** You just want that to also indicate that bush meat also has high quality medical value.

**Moderator:** Okay, I would love to hear this too.

**Male Participant:** Yes and then it ranges from the treatment of infertility-- [crosstalk]

**Male speaker:** Yes you said it the other time and I wanted to-- [crosstalk]

**Moderator:** Infertility?

**Male Participant:** Yes. Male infertility actually.

**Moderator:** It contributes to male infertility?

**Male Participant:** No. It treats male infertility.

**Moderator:** The treatment of male infertility?

**Male Participant:** **[unintelligible 02:00:54]**

**Male speaker:** This guy is presenting his [crosstalk]

**Male Participant:** No, I just want to [laughs] I have realized that most of the medicines that I use to treat male infertility have some level of-- how do you call it, protein in it.

**Male Participant:** Protein.

**Moderator:** Protein.

**Male Participant:** The preference for antelope, especially the roan antelope is very high, because it is said and believed that the level of protein in **[unintelligible 02:01:33]** is quite high and that was used in the treatment of infertility, in conjunction with **[unintelligible 02:01:41]**

**Moderator:** What kind of bush meat, any kind of bush meat?

**Male Participant:** No. The antelope to be more specific.

**Moderator:** Antelope?

**Male Participant:** Yes it's what is the roan antelope, exactly. Then again, when you look at some other medicine, for example, as a psychologist, anyone who has done developmental psychology will tell you that we have a period where the veins and muscles of a baby are strong enough for the baby to be able to crawl, or to be able to walk, or to be able to stand, and so on. That instance can be hastened with bones and some other parts of bush animals. Especially those that are very strong like the elephant, like the lion, and so on and so forth. They have a way of preparing them together. They prepare them. You may think that some of this may have some psychological underpinnings.

**Moderator:** Like a placebo effect you mean?

**Male Participant:** Those that they use them on, they walk faster and earlier than even their siblings who didn't have that.

**Moderator:** They walk faster?

**Male Participant:** Yes. Let's say for example in the family where you have children walking after nine months, a child who has been bathed with these things will walk after seven months.

**Moderator:** Is that a study out of the--

**Male Participant:** Yes. Just coming from the **[unintelligible 02:03:11]**

**Moderator:** From the?

**Male Participant:** The data, I am doing some research work too I have realized that some of this is actually contributing to the treatment of some diseases, and some sickness. Maybe it's an angle that you can look in that.

**Moderator:** Absolutely, because for me, I would imagine that that has academic merit. If you have your methodologies there, and this is the second year, third year, are you done?

**Male Participant:** This is my fourth year.

**Moderator:** Yes, and you've done work here in the Netherlands?

**Male Participant:** No, in Ghana.

**Moderator:** In Ghana, so you're at the university there, the medical center?

**Male Participant:** I am a student of the University of Leiden here in Holland as well as University of Ghana in Ghana.

**Moderator:** Wow, so that must be really exciting.

**Male Participant:** Yes it is exciting. When it comes to this bush animals as somebody indicated earlier on that **[unintelligible 02:04:12]** another person said that it didn't matter whether it was dead or not **[unintelligible 02:04:19]** If you listen to the hunters, you would come to the conclusion that most of the medicines that we have, what we call alternative medicine, or we have a medicine in Africa actually is as a result of observation of animals. What they eat when they are sick. **[unintelligible 02:04:38]** what kind of leaf or herb? Would it take the bark of a tree? They continue to watch this, and after a while, they gather some information to be able to tell that A+B+C will be able to treat Y.

**Moderator:** The animal reservoirs, the animals that have the reservoirs, I heard the University of Ghana is really cutting edge with understanding which animals, which species of bats have the reservoir for these certain diseases to then be passed on to people who would maybe pick up an animal on a forest floor and bring it home then butcher it. Is that also what you've found when you were in-

**Male Participant:** Not really, but the hunters are so experienced that they can tell what kind of animals that can be gotten from this reservoir. They have **[unintelligible 02:05:41]** Some of the information are transferred from generation to generation. Some of them are generational information. There is some who subject them to some kind of proof, evidence. They want to find out what was found, and if it's true and it's working.

**Moderator:** You have to research it. In America, there was a study done, and I think he was Nigerian, he did a study, and I can send it to you. Basically, apparently during final examinations, his mother would send him, it was primate meat to say, "You will be able to concentrate better, you'll be able to do better on your exams if you eat this kind of meat. It was from generation to generation, this was something that-- But if you do the research, then you can actually prove it, because it's things that you hear about like you said, but until you do the study-

**Male Participant:** To be able to prove that-

**Moderator:** -to prove it. People don't believe it, and so you can actually do the research.

**Male Participant:** Exactly.

**Male Participant:** I'm eager to find out, how do you consume the squirrels in the north?

**Male Participant:** At least in my area, we don't throw anything away. That is exactly the point I'm trying to make, we don't throw anything away. **[unintelligible 02:07:24]** or the grass cutter. The squirrel, the head **[unintelligible 02:07:33]**

[crosstalk] [Ghanaian language]

**Moderator:** You don't throw anything away.

**Male Participant:** Is the grass cutter I know they don't throw anything away. They know what they do with the feces?-- [crosstalk]

**Male Participant:** The grass cutter, we throw some amount of it away. We pull the feces out, and the we thread it **[unintelligible 02:08:23]** We rarely throw, in our case, we throw the--

**Moderator:** Even don't they add a spoonful of the green contents of the stomach inside for the soup?

**Male Participant:** You see when you take the intestines and squeeze everything out, some remain in there. At least the taste they want to get is also within-- Because when you wash it, you don't wash the inner part of the intestines.

**Moderator:** It gives it flavor?

**Male Participant:** That gives it flavor.

**Moderator:** That's really interesting of your study. You're going to be presenting it?

**Male Participant:** Yes. Around January. I think the first draft will go out in January.

**Moderator:** That's exciting. That's a lot of work.

**Male Participant:** A lot. Four years of work.

[laughter]

**Moderator:** Yes. Congratulations.

**Male Participant:** Thank you.

**Moderator:** I think they definitely have not seen any study like that, and it's going to possibly move the other researchers forward to look at that.

**Male Facilitator:** Which department is that?

**Male Participant:** In Leiden, the African Studies department **[unintelligible 02:09:37]** I have always been interested in the issues that have to do with medical anthropology. For my masters, I looked at mental children and how they are taken care of **[unintelligible 02:09:53]** Then the perception of people as far as mental disability is concerned. That's what I looked at.

**Male Facilitator:** So, who's supervising your-

**Male Participant:** I have a **Ryke van Dyke.**

**Male Facilitator:** Ryke.

**Male Participant:** You know Ryke? He's my main supervisor here. Then I have **Dr. Dotchmund** from **[unintelligible 02:10:16]**

**Moderator: [unintelligible 02:10:17].**

[laughter]

**Male Participant:** I always pronounce it from an English angle, but I've always been told that is wrong. I've always asked them to pronounce it for me.

[laughter]

**Moderator:** Yes, that sounds good.

**Male Participant:** Are all your questions answered?

**Moderator:** Yes, thank you.

[sound cut]

**[02:10:42] [END OF AUDIO]**
