# Supplementary material for: Exploring the characteristics of a local demand for African wild meat: A focus group study of long-term Ghanaian residents in the Netherlands
Source: PLoS One. 2021 Feb 16;16(2):e0246868. doi: 10.1371/journal.pone.0246868 (PMC7886224; doi:10.1371/journal.pone.0246868)
Supplement: S2 File — (DOCX) [file pone.0246868.s006.docx]

**Africa Focus Group: Session 2**

**Coding key**

| **Pink** | **Luxury Item/Status** |
| --- | --- |
| **D.Blue** | **Willingness to Pay (WTP)/ Bushmeat Price Comparison - Ghana vs. Netherlands** |
| **Yellow** | **Food Preparation/Taste** |
| **Green** | **How to avoid infection / Health considerations** |
| **Purple** | **Generation changes in bushmeat consumption** |
| **Red** | **Species** |
| **Orange** | **Local Demand & Trade/** **Methods of Acquiring meat locally** |
| **L.blue** | **Methods of hunting** |

******Notes on body language, gesturing, tone and volume have been made in blue italics transferred from notes taken by the interviewer during the discussions.******

**================================================================**

**Interviewer:** That’s a cassava

**Adante:** This is rice [crosstalk] that is soup, that is meat.

**Interviewer:** What kind of meat is this one?

**Female Participant:** Cow meat.

**Interviewer:** The stomach? This is Punao?

[Laughter]

**Interviewer:** You guys can easily trick me, that’s the thing, you can easily tell me things, I don’t know enough yet. How long does it take to prepare it?

**Female Participant:** Three four hours, because you have to prepare everything separately.

**Interviewer:** And can you find everything that you are looking for?

**Female Participant:** Yes.

**Interviewer:** Is it the same price as in Ghana or?

**Female Participant:** Actually for a lesser price in Ghana than here.

**Adante:** Sandella what is this?

**Interviewer:** What is this? This is my tea that I was drinking, sorry.

**Adante:** So that I don’t put any drug in it.

[Laughter]

**Interviewer:** This one is free, this one is open, if you want to put something, go ahead.

**Adante:** Sorry? Yes, I’m through. [Cross talk].

**Female Participant:** The soup, once you have taken it you have to beat the **[unintelligible 00:02:37]** base like this, otherwise you take only oil.

**Interviewer:** Okay, I see. That smells really good.

**Female Participant:** You can dip it in the water then it can be easy for you, dip this in the water then it is easier.

**Interviewer:** Okay, thank you. I'm going to do this the way you ask. That smells really good.

**Female Participant:** You can do the red beans, the red rage.

**Interviewer:** The red rage, with this oil because it is red, but what makes it red?

**Female Participant:** The palm soup, the palm, the palm nut.

**Adante:** The palm oil is red.

**Interviewer:** Palm nut sorry, all right thank you.

**Adante:** The palm nut, what makes it red? [Cross talk] the fruit and the soup, the oil is taken out of the-

**Female Participant:** Palm fruit.

**Adante:** Yes.

**Interviewer:** I hope I'm doing this right. Are you watching me to make sure I don’t make any big mistake? Embarrass myself?

Then you put the beans in there too?

[Foreign language]

**Female Participant:** Forceps, for cooking, but not for-- He thinks the forceps are for operation. He is a man, he doesn’t go to the kitchen.

**Interviewer:** Yes, forceps, is that what you were talking about Adante? Thank you.

**Adante:** No, I was still talking about the forceps which-

**Interviewer:** I was born with forceps.

**Female Participant:** You wouldn’t come out?

**Interviewer:** No, I would not come out.

**Adante:** How did you know? You were too small to know.

**Interviewer:** My mum told me, my mum was a midwife herself in Switzerland. She did her studies in Switzerland and she has the craziest stories, about delivering babies before all the technology existed, you know. She says, now in America when I had my babies she looked at the gynecologist and, my mum is small like me, but she has a very big mouth, and she said, "You don’t know anything," because I asked you if you would deliver at home, and the lady said- the gynecologist said, "I'm sorry I cannot do that." She said, "Legally, I understand but would you like to do it?" She said, "I can’t, if I don’t have my technology I wouldn’t know what to do." My mum said, "That’s serious that’s really serious," because in the old days they would look at your nails and look at your eyes, and my mum said she could tell when a woman came inside immediately, this one is going to give me a bad night. She could just with the diagnostic, personal diagnostic, see it from everything, but here now everybody want blood tests, and scans, and this and that. Then they become too dependent and they lose that touch like the old doctors still have it, they can look at you immediately and say, "Something is not right." and they are okay, "Let’s get some lab work let’s get this lets get that." I think its different. Are you eating?

**Female Participant:** No, not now.

**Interviewer:** No? Okay, and are you eating? Because I’m going to wait for you before I eat.

**Female Participant:** No.

**Interviewer:** No? [Cross talk] I shouldn’t start before everybody is served.

**Female Participant:** Some are almost finished.

[Foreign language]

**Interviewer:** Very tasty. See this is spicy. The last time remember we talked about spices, if there is more spice in Northern Ghana, or Eastern Ghana, or Southern Ghana and somebody told me it doesn’t change. The level of spices in food doesn't change, like in India or in Thailand, those like Northern Thai very spicy, in India also there is areas that are more spicy than others.

**Adante:** I disagree to that statement, the spicy depends on where you are, and what type of spice you are using.

**Female Participant:** Depends on the person eating it, or the person cooking it.

**Interviewer:** Personal taste okay, we did talk about that. If you grow up with parents that spice a lot then you grow up wanting spice, yes okay. My son adds Tobasco like we have to go through one bottle every week. I don't know where he is going, and then he needs more and more and more, just like salt. When you add salt at a young age, and then you add more and more salt older, so maybe it’s the same.

It's very good.

**Adante:** Very nice.

[Foreign language]

**Female Participant:** I don’t like the spoon.

**Adante:** People enjoy eating with their hands more than the spoon.

**Female Participant:** Even at home with a **[unintelligible 00:10:01]** I just want to sit on the ground and eat and open it. Yes. **[unintelligible 00:10:06].** We have to go back. There's still time.

[foreign language].

**Interviewer:** I went to Ethiopia, and they eat with their hands, too. I ate with my hands. Just did it like everybody else at the table. It's nice.

**Female Participant:** Yes. Only if you know how to pick it.

**Interviewer:** Yes. At first it was not so easy. [chuckles] Same thing when I learned how to use chopsticks. At first it's not easy. Maybe the spoon changes the taste, too.

**Female Participant:** Yes, the spoon [crosstalk].

**Adante:** With the spoon?

**Interviewer:** Yes.

[foreign language]

**Interviewer:** What's the best month to visit Ghana?

**Adante:** The only month I wouldn't advise is June and July.

**Interviewer:** Don't go in June and July?

**Adante:** It's too rainy.

**Interviewer:** It's the rainy season.

**Adante:** The rest of the time, you can go any time.

[foreign language]

**Female Participant:** Even in Paris, it was not very hot.

**Adante:** Was nice.

**Female Participant:** It was nice.

**Adante:** It was very nice. That was in. Actually, this year's it's all been very nice all over **[unintelligible 00:13:26]** the rains. June, July.

**Interviewer:** Yes. That's true. We got a lot of extreme heat in the south of France. Really almost horrible. Really, really hot for older people without air conditioning. This is difficult. I wonder about them, but we got extreme heat. Then, we went to the mountains just to get away. Apparently in Europe, more and more people are getting air conditionings. Whereas, before they never needed it. These air conditionings. They get so hot and uncomfortable.

**Adante:** With all of this, I've never used air conditioning in my room. Yes. It's strange. The water's so cool.

**Female Participant:** In the morning, I can put them on, very nice.

**Male Participant:** Especially at our place **[inaudible 00:14:28]** the mountains.

**Adante:** In my village. In my town, Kwame, very cool. Very cool.

[foreign language]

**Female Participant:** They have to come down.

**Adante:** You know how to build a house. It's now built in that area. **[unintelligible 00:15:26]** area.

**Interviewer:** Where is that area?

**Adante:** The mountain area. Where she comes from.

**Interviewer:** Okay.

**Female Participant:** I took a photo when they were standing on. "It's really nice. You have to come and live here**."** Yes, it's under works. [chuckles]

**Interviewer:** Is it expensive to buy land there?

**Male Participant:** Not really, no.

**Interviewer:** Are the prices going up?

**Male Participant:** The price is going up, but you can still afford it. It's nice. The temperature is very cool like here. It makes living there a little more better than in **[unintelligible 00:15:55].**

**Adante:** I went to Okubwa, it's somewhere to south. Very nice environment.

**Interviewer:** Delicious. Compliments to the chef. ou.

**Female Participant:** Thank you.

**Interviewer:** Can I put this is the trash maybe?

**Female Participant:** Yes.

**Interviewer:** It's good for the sinuses.

**Female Participant:** Yes. Helps with everything.

**Interviewer:** Yes. It really. [laughter] I don't know where Kwasi is, but he didn't write or anything. He didn't write anything.

**Adante:** You can start?

**Interviewer:** Yes.

[background conversation]

**Interviewer:** I'm okay. Thank you.

**Female Participant:** Okay.

**Interviewer:** Thank you very much.

[foreign language]

**Adante:** Amutu is ready. You can eat, Amutu, before we start. We are starting the program now. We have come to talk about bushmeat.

[foreign language]

**Kwasi:** Well it's now almost 2:00, so we have about one and a half hours to leave. Do you think it will be enough for you?

**Interviewer:** Yes.

**Kwasi:** To formally introduce Interviewer, she's doing a research, as she introduced herself already. She's doing a research about bushmeat, in fact, Cocochino invited us Kwasi and myself, to see if we can help with the community to support in her research part. The first time I spoke to her was on, what was it?

**Interviewer:** Yes, Skype.

**Kwasi:** Yes, Skype, she was in France at that time, and we met, if you remember even our party she came because we invited her to our party, so at least you've had some interaction as of now. The direct questions she wants to know if she can-- I'll keep quiet because I've participated in this program before, so I'm observing only. The rest, I'll leave it to her to start her questions.

**Interviewer:** I'm very new at focus groups but it's an opportunity to learn about a topic that I think there isn't very many opportunities to share information between communities. Coutinho and I are interested in culture and nutrition, and what people feel about this kind of food, and it's a big area. A bigger area than I thought, because I didn't realize many things during the pilot and the first meeting. There was a gentleman who said, "Yes, but it's healthier than domestic meat," because of things like, the animals that are in the wild are leaner meat and less fat and healthier, and you know where it's coming from, compared to lots of these manufactured produced meat that has hormones, or it's chicken that has chemicals and this kind of thing.

I didn't realize that it was a nutritional thing, I had thought in my mind that it was just a cultural attachment like I'm attached to my food in France. Some of that food is also, there's very little known about why people eat certain things in France like **[unintelligible 00:23:48]** or horse, and some of the other things that we eat there.

That is what we embarked on, and we are trying to also figure out what are the health risks that you perceive? Are there health risks? Yes/no. What could they be? How do you prepare the food to make sure that you kill anything that's in it, germs or anything like that? Some people believe smoked, some people believe boiled and things, and then just the different species. What are the different animals that people taste and like yes/no.

Then finally, the more money you have, what animals would you buy if you had all the money in the world, which ones? Socioeconomically, which ones go with the more money you make, this kind of meat you want and. Similar to I would say how here when you have more money, you buy better cuts of meat, or you buy lamb chops, or you buy duck breast, or something like that. Similar to that kind of thinking.

Then we talked about last week how much it cost per kilo in Ghana compared to how much it cost here. Then there's a lot of discussion about that, and then also where you get it. Do you get it from your butcher? Do you get it from friends who bring it over? Those were the things that we talked about in our last two meetings. That's a summary of where we are with it.

We also talked about the difference between, because it's funny all the words that I learned, so there's a word for the monkey that was in the bush, and a word for the monkey that was in the home, and you don't eat the monkey that it's in the home, but you eat the monkey that was in the forest. For me at first it was really confusing because how do you have two names for two different animals that are the same animals. Adante helped me understand it, for me it was enlightening, so I left with a lot more knowledge than I started the day with.

**Kwasi:** What is the name of the one in the bush and home?

**Interviewer:** Okay, I don't have that with me right now but what's the name? You are giving me an exam. I have it written down, I had a whole page of different terms. The ones I remember is eto, and punam and I remember, I don't know.

**Male Participant:** You need one more month to listen.

**Male Participant:** Do you remember adwi?

**Interviewer:** Pardon?

**Male Participant:** Adwi.

**Interviewer:** Yes. Was that what we talked about? Is that the word that was used for the--

**Kwasi:** The one at home?

**Male Participant:** No, I think we spoke about, I was saying that we eat everything but people had the opinion that **[unintelligible 00:27:17]** and then the one at home **[unintelligible 00:27:21]** you don't eat it. I had a different opinion but of course--

**Kwasi:** I think the forest, the monkeys have the largest species, so many species, they have the biggest families, several species more than any other animal.

**Interviewer:** Yes. There isn't one particular that's tastier than another one? They all have the same flavor?

**Male Participant:** It depends on the perception of the person eating it, people may assume that once the animals are at home, they are not tasty as good as the ones in the forest.

**Interviewer:** They are getting exercise?

**Male Participant:** That is why they are bringing at home, they don't want to eat it but then they are doing the bush, they would like to eat it but for me I think that adwi is adwi. The meat will be the same depending on how you want to prepare it or you want to eat it.

**Interviewer:** Yes, how you prepare it.

**Female Participant:** If you have adwi at home, you wouldn't like to eat it.

**Male Participant:** That is why I'm saying it depends on the ideas people have.

**Female Participant:** Because it becomes your pet.

**Interviewer:** That's what they said.

**Male Participant:** Then you don't feel like eating adwi at home.

**Male Participant:** The person who is really looking for adwi meat, when he gets the opportunity--

**Male Participant:** The idea is that once you know you have the adwi at home, the adwi becomes your friend, then you don't feel happy eating it. When the same adwi is somewhere and you don't know where the adwi is coming from, you'll never know it's the adwi from home you still enjoy it as the ones from the bush.

**Male Participant:** They are all eatable. If somebody steals it, he will kill it in the house and eat it.

**Interviewer:** How do they hunt them? Is that changing overtime, or is it staying the same? Last week, I'll just give you some context, they explained, and this is the best part. This was so funny, but they were saying, "Have you ever tried to catch a cane rat?" It's hard to catch a cane rat, but the cane rat has a name. He was saying then sometimes they use smoke, and sometimes they do traps around the cornfield, and then he said, but the problem in the pilot is, the one gentleman and it's just one gentleman who talked about this. He was concerned about poison although you also talked about poison.

**Male Participant:** That is the grass cutters.

**Interviewer:** A grass cutter yes.

**Male Participant:** Using the **[unintelligible 00:30:04]**

**Interviewer:** Yes, so how do you choose where you get the meat so that you are confident? How do you choose the meat so that you know that it's safe, and that people aren’t using those methods?

**Male Participant:** Personally I don’t think -- You see what we're saying here there are two different things here. Is it meat that I’m going to catch it and come and eat it, or meat that I should eat it? If it is somewhere I have to go and catch it then maybe I have to go and catch it from the forest, but in most of these cases we have people who go there to hunt them, and we buy them from the market or you buy them on the ways side. When you're driving on the ways side you see people have these smoked meat animals there. We going to buy them actually we don’t know where they come from, all that you know is that there's an animal there which has been killed somewhere and it has been dried so then you buy it from them. How they manage to get it there and get it dried you can't basically know.

**Interviewer:** Yes, so you don’t go back to the same person the same butcher each time?

**Male Participant:** No butcher sells **[unintelligible 00:31:29]**

**Male Participant:** No, we don’t have one person selling akaranti. For example, it depends on if I’m in the village, somebody goes to the village and maybe kills akaranti. There's a farmer who works there and they do this trap setting, they know how to fix this trap along their control to trap those animals. They trap and kill them then bring them home. Then when I see that I like it or when I go to the market, I buy from them.

If you're in the big city those animals are killed somewhere in the village, and they have to transport them to the big cities in the market. You go to the market and then somebody in the market who sells those things, so you go where they sell those things and then you buy it from them. You may not have idea of how they were killed, or how they were smoked, the only thing you may know is you have a dried bush meat there and then you buy them from there .

**Male Participant:** Basically people do go out there and hunt, hunting for those specific animals. They are sometimes way deep in the forest and then people have to go in there with their guns sometimes, or spear or whatever or setting some traps all over the place. Some specific people go out hunting, they go hunting and then they can get a smaller animal or a bigger animal. When they get a bigger animal they can decide to sell the whole piece or work on it. When they work on it that is when it becomes in the olden days, you kill an elephant or you kill a bigger animal, or a deer whatever, you cannot eat it alone, so you bring it home you prepare it, there was no fridge. That is where the mpunam comes in. You smoke it and they hang them or sometimes they have a basket and they put them in, it's a way of preserving them, so mpunam actually is the smoked one preserved. They have preserved it for a longer time.

**Interviewer:** I thought it was a mixture.

**Male Participant:** I remember I was in Bulgaria or Albania, Montenegro and we went to the guy’s family house. We sat in the kitchen with a big fire and the meat where legs of the thighs they were all hanging there, and we were just cutting it and drinking, and cutting and drinking, and cutting and drinking. That for me it was also a way of looking at that is mpunam, and mpunam can be in different forms. When you go to the sea, for example, and you get a whale, you cannot eat it all. They smoke it for preservation, so we have fish that is also mpunam if you can get a fish also mpunam. They can get also the bush meat as mpunam. It's all about the preservation for a longer period.

**Interviewer:** For a longer period, and is that also the best way, in your opinion, to kill the germs or is there a better way?

**Male Participant:** You see killing a germ, the animal we are looking at it personally we're looking at it from the environment of the animal. If there is no chemical, nowadays they use chemical to weed and other things so those pesticides will go straight into the bloodstream of the animal, but deep in the forest where these animals are there they are eating natural everything that's there is natural. We eat even the intestine, the shit in the intestine we eat it. We call it Buffu. It is a nice piece, you eat it and **[unintelligible 00:35:20]** you would just be smelling [laughs]

**Interviewer:** Because it’s natural.

**Male Participant:** It’s natural.

**Interviewer:** It’s organic.

**Male Participant:** Basically it's more organic.

**Male Participant:** They chew organic grass.

**Male Participant:** They are chewing everything organic, we don’t even think that there is these germs in there, germs no. When it dies, it's dead down there and some people go and eat it, it's a nice one. You don’t know what killed it, but you just bring it home prepare it **[unintelligible 00:35:50]** but nowadays everything that you touch they say germs

**Female Participant:** We're not even thinking of the germs.

**Male Participant:** We're not even thinking of that. It’s more like the organic, it's more organic.

**Interviewer:** Yes, which is what everybody else is trying to find, and paying a lot of money for it by the way. They're are willing to pay, their willingness to pay is--

**Male Participant:** The last time of these latter days, people are rearing them and giving then artificial foods that is where sometimes it's bit tricky. We've chased them all out of the bush, we don’t give them enough time to-- The habitats are being taken over. Look at all the forests they're now messing the forest up, and if the forest is being messed up the animal, the ecology the **[unintelligible 00:36:46]** is also taxed.

**Interviewer:** Who’s messing up the forest, the logging companies?

**Male Participant:** The logging companies, gold mining companies and land degradation. You look at **[unintelligible 00:36:59]** for example, forest there's big forest nice place, but the river body is now turning yellow or brown. it's sad.

**Male Participant:** The other time I was saying that for example if I’m driving along **[unintelligible 00:37:15]** to cape coast, I won’t buy the akaranti along the road because I’ve done it once and I had--

**Male Participant:** Diarrhea.

**Male Participant:** I’ve heard that they use chemicals, so I never buy there. If I’m driving from let's say Accra to Kumasi in our area probably between in **[unintelligible 00:37:38]** with Sino between Sino and Unem you see them standing along the road, I might buy it. Because my experience is not like the experience I had. These are personal differences.

**Male Participant:** That is an illegal way of killing those animals. I also know that some people also use urine fermented one. If it really get it gets fermented it turns into ammonia it is buried in a container **[unintelligible 00:38:11]** that one and catching them.

**Interviewer:** Yes, they didn’t talk about ammonia, but they talked about formaldehyde, remember they did, you're right, this new techniques. This seems like, my impression from what people are saying is that this is new. This is different than the way that it usually was happening, and that before it didn’t sound like people had a feeling that they had to be careful, but now maybe a little bit more careful.

Then are you seeing that they're the same species or that the species are changing? People’s tastes remain the same, or do you feel like maybe the tastes for different kinds of meat are changing, or maybe you're seeing less species if they're not available, less available. Maybe there's some species of meat or some kinds of meat that growing up you saw them everywhere, and now you don’t see them so much anymore. Is there anything like that or has it remained the same?

**Male Participant:** When we were growing, we were growing in the bush.

**Participant:** Or do you mean in the villages? And now we've left the village and then come to the city?

**Interviewer:** Yes.

**Male Participant:** We see less of those animals but people in the villages, they see them all the time, but if you're in the city it doesn't become **[unintelligible 00:39:53]** but that's a different thing, but they are there. It can be that the species there a lot of species available but who is counting them?

**Female Participant:** Hello.

**All:** Hello

**Male Participant:** The number definitely might be reduced looking at the environment. Their habitats have been taking over, what is happening to them, and whenever they see someone right out, the people will start chasing it and then get in the way.

**Interviewer:** Does that make the price go up?

**Male Participant:** In a way there's scarcity, my economics and then that makes **[unintelligible 00:40:32]**

**Interviewer:** Supply and demand yes.

**Male Participant:** That is also an area where the government has also been having these reserved areas, those reserved areas are all sort of protecting- [crosstalk]

**Interviewer:** The food source, so is that a food security issue.

**Male Participant:** It is a food security issue.

**Interviewer:** The government helps to make sure that-- [crosstalk]

**Male Participant:** Many forest reserves, there are a lot of animals in there, but there you don’t have to go there ever. One there is a guard, or there is a security there, if you trespass and then you can be caught and arrested.-- [crosstalk]

**Female Participant:** And the government does that consciously because of the animals there, so that they can be hunted?

**Male Participant:** No, no, no not for hunting purposes, they are there, but of course, some animals can slip over and around somewhere and be the scape goat, but at the same time some hunters they go deep into that forest and kill.

**Male Participant:** Illegal killing..

**Male Participant:** I know they allow it people to hunt but you have to hunt specific species of animals.

**Interviewer:** Okay so controlled hunting.

**Male Participant:** Between August and November, all activities have **[unintelligible 00:42:01]**

**Kwasi:** Today it was in the news.

**Interviewer:** You will all learn together.

**Kwasi:** It was in the news that the forestry department was chasing people out of the forest.

**Male Participant:** People enter it illegally.

**Male Participant:** They were addressing timber.

**Male Participant:** Timber logging.

**Interviewer:** What's that? Timber?

**Kwasi:** Timber.

**Interviewer:** Illegal loggers, illegal logging, okay.

**Kwasi:** Yes.

**Male Participant:** It's just to protect the land and then protect the-- [crosstalk]

**Interviewer:** The bio diversity. Yes, I realize that if 80% of the protein in land locked countries comes from the bush, because they don’t have-

**Male Participant:** Fish.

**Interviewer:** Yes fish, then you can imagine that without that food source that there could be a disaster as far as malnutrition and stunting. I know that the World Bank works very hard in Africa, and throughout the world with nutrition between the age zero and eight years old, and if they don’t get enough nutrition at that point then they could be stunted. So yes, that kind of meat if it is not available could be a problem, I understand that.

Then last week we talked about also how bush meat tastes different here, in the Netherlands and Kwasi, actually was the one that said it tasted different and everybody else said, "That's ridiculous it doesn’t taste different," maybe it's because when it's transported it's frozen, thawed, re- frozen, thawed, but then that doesn’t make sense because some of it comes over very quickly. I know that when I buy Rosa wine in France I can drink it all summer long, and I can buy one case and bring it back to the Netherlands and I drink the Rosq and it tastes completely different, why? It's the same bottles of wine. I was trying to figure out is it just an impression, like psychologically does it taste different when you eat it here, or does it actually taste different when you eat it here?

**Male Participant:** I think it tastes differently when you eat it here because of the transportation. Normally at our place it is dried to-- When it is dried and fresh it has got certain amount of taste the quality is different.

**Interviewer:** Flavor.

**Male Participant:** Flavor yes, once the meat keeps longer then it is losing some sort of taste especially if it is not well dried.

**Interviewer:** How long typically does it need to be dried for it to be the right- [crosstalk]

**Male Participant:** We don’t know how long it takes. I can’t imagine, but all that we know is, the more dry it is the better quality it is. Because when you have two bush meat and one is really dried and the other one is something in between, the one which is not very dry doesn’t taste good as the one which is dried.

**Interviewer:** I thought-- Okay well then I’m learning something, I keep learning every time anybody says something it adds more layers, because last week, I have a good memory but you're going to correct me if I’m wrong, I thought that when it's too dry there's a taste in the back there's like a- [crosstalk]

**Female Participant:** Bitter.

**Interviewer:** -a bitter taste in the back when it's too dry, but some people like that taste is that what you are saying?

**Male Participant:** No, it depends on what you mean by dry. You know normally when we dry it, what the bush men has got is that the dryness always takes the water from the meat it is the pure water but not the taste, that is why the more it is dried the more how long you can keep it, preservation. The more it dries the more you can keep it longer, and once it is dried it means most of the water has gone out of the meat.

In that sense you can preserve the meat for longer. Even when you travel with then it wouldn’t affect the taste of the meat, because mostly the changes in the meat is caused by the water content in it, so when it is dry you can transport it for a longer period without affecting it because the thing is dry there is no water in it. There is no water to cause chemical reaction and thereby changing the taste of the meat.

**Interviewer:** That makes sense.

**Male Participant:** So when you have a dry meat transported from there to here, which of these days it comes within a week or two, then it shouldn’t affect the taste of the animal. When it is not dried and you bring it here, they know it when you go to buy it, it smells so there you know there is some reaction gone by then it changes the taste. So yes, if you have dried one, very dry from there to here, it shouldn’t change the taste. It should have exactly the same taste, unless it is not dried, and mostly they know when you bring it here and you un-box it it starts producing some smell.

**Interviewer:** Yes, so smoked dried it preserves the flavor?

**Male Participant:** The drier the fish becomes, one, the good taste it has, and also how long you can preserve it. Even when you bring it here for even two, three months even at our place with dried ones they can keep it for half a year without having effect on the taste. The little science about the bush meat is that the taste depends on the amount of water in the meat, so the more dry it is, it means the water has been taken out of it, and once the meat doesn’t contain any water there is no chemical reaction going on.

That’s why if you go to a place some of them keep them the whole year out there, at our villages we have the kitchen there and we have a net or maybe some sort of shelf on top of the kitchen. The meat are put there, and everyday once they are cooking the smoke goes over the roof and it keeps on drying it so it has no contamination in it and no animals, no insects.

**Interviewer:** It's pure.

**Male Participant:** It can stay there for the whole year, it is still there. With such thing they can keep it even for one year, because the simple reason is that it is losing always the water in it and once it is dried then you can keep it as long as you want, and it will maintain it's taste.

**Male Participant:** What about **[unintelligible 00:49:05]**

**Female Participant:** That is when it's not dry.

**Male Participant:** **[unintelligible 00:49:12]** means fat is rotting. [background noise]

**Male Participant:** For Fungus.

**Female Participant:** Sometimes you can see worms in it sometimes.

**Participant:** Any time you see any animal, be it bush meat or fish, with fungus it means there's water in it. It is not dry.

**Interviewer:** Right, obviously.

**Male Participant:** But then the more it smells the luckier it becomes. Or not.

**Interviewer:** That I can understand, because with the cheeses, I’m just going to tell you the cheeses in France some of my friends from America they come, and they open the refrigerator and they pass on the ground they pass out. Like it's the smell is just so bad to them, for me it's amazing. I love it.

**Male Participant:** But it's nice.

**Interviewer:** It's really nice, so I can understand strong, it smells, but it's what you're used to. That was my biggest, I think, question about trying to understand food and culture, is that what is it about African wild meat that when you eat it here, do you eat it because it's healthy, do you eat it because it reminds you of home, do you eat it because it's a community, it's a bonding of a community or is it all of those things, is it none of those things?

**Male Participant:** Well, apart from being, it's rich, which know from Ghana. It is organic rich, because of the animals, let's say they eat pure grass from there. To us, we consider it to be rich in vitamins. It's not simply because-- You see, those meats are not eaten simply because people are poor. Even the rich men and poor men, we are all competing for the same meat. What it is that why is it something we are used to. We've grown living with that idea of that social life the delicacy that's used that way.

We've been eating this from our infancy so it's part of our culture. We can't even do away with it everywhere we go. If not, we wouldn't be sitting here 30, 40 years and we sill like to eat that one. It is our culture, something we've eaten since **[unintelligible 00:51:25]** We love eating it, it's part of our food everywhere we go, so long as we are on this earth, we eat it everywhere we go.

**Interviewer:** It makes sense because that's what culture--

**Male Participant:** We eat it not because it tastes better from there or here, but we eat it because it's part of our food and we love to eat it. Unfortunately, here it's too expensive and you can't be eating it all the time.

**Interviewer:** That's what I heard. I heard that--- Well, I'm not going to influence your answers, but we asked the last group how much it costs here compared to there. Everybody had a different answer, but at the end, it did seem like there was a consensus about whether it's more expensive or less expensive or the same. I'm going to ask all of you, is it that much more expensive? Because the numbers that they gave me are very high, very expensive.

**Female Participant:** I think it's expensive. I don't even try buying it, because when you go to the shop, they tell you a small piece, 15 euro, 20 euro.

**Interviewer:** That's what they told me.

**Male Participant:** If it comes here, it's an imported commodity. we can't **[inaudible 00:52:41]**

**Interviewer:** That's what they said. They gave me a range, I'll give you the range that they gave me. They said 100% more, 200% more, 25 euros a kilo. Do you agree with that pricing?

**Female Participant:** I've never asked for the price.

**Male Participant:** It's just like you are in Ghana, you want to go and buy fridges, you'll buy it at a very high price.

**Male Participant:** Also, don't forget that even though we want eat those things, it's also affect our budget. Economically, even though we are here, it doesn't mean we can be eating, but it's expensive to our budgets. It's not something that I can say I'll be eating it all the time. People here, we eat it occasionally, because when it gets here, it's more expensive. You can buy it may be what they are saying 100% more the price of what it's there. You wouldn't like to eat all the time.

**Male Participant:** Which occasion would want to eat?

**Male Participant:** No, we don't have a specific occasion but it's just like-

**Interviewer:** It takes a while.

**Male Participant:** -when we stay for a while, a longer time, then you feel like eating your own food.

**Male Participant:** You see, because of the price, here, if you want to make very good soup, you don't have to put a lot, you just want small pieces to make same taste.

**Interviewer:** That's what you were saying the last time, a small piece can give the flavor. That it doesn't take very much.

**Male Participant:** I wonder if somebody would go and make real akaranti soup here?

**Male Participant:** No, I don't think so. It won't happen.

**Interviewer:** Why, because it's too expensive?

**Male Participant:** Yes, it's too expensive.

**Male Participant:** In Ghana, you can buy a whole piece and--

**Male Participant:** Then use it to prepare your meals.

**Female Participant:** Maybe if you can bring the akaranti here yourself, you can make it.

**Female Participant:** Even if you bring it here, will it be wise keeping it long?

**Male Participant:** If you're able to bring one here--

**Interviewer:** You will just take a piece of it.

**Male Participant:** A piece of it.

**Interviewer:** Then you would dry the-- How do you say it? You used the name.

**Male Participant:** A Kranti.

**Interviewer:** A Kranti, you just--

**Male Participant:** Put it in the deep freeze.

**Female Participant:** You put it in the deep freeze then-- [crosstalk]

**Female Participant:** It's dried already, so you take some, you just want the scent in it.

**Male Participant:** **[unintelligible 00:54:54]** to do that is an essential commodity, because it's not easy to pass it through the airport. It's not easy at all.

**Male Participant:** You don't need the airport to bring on this.

**Interviewer:** Yes, you don't. I would imagine--[crosstalk]

**Male Participant:** If they catch you, they will just take it and then throw them away.

**Interviewer:** That's what I heard.

**Male Participant:** If you are lucky and you are able to pass. It will help you **[unintelligible 00:55:16]**

[laghter]

**Male Participant:** Here, we don't eat them but we only just smell the taste. We only put like this into the whole soup, just to give you the flavors. Whereas at our place, we just eat it because we can buy it, we just eat it. Here, one akarantie can even stay for six months, because here, you just put a little to give you flavor in the soup.

**Interviewer:** At least you know where it's coming from. If you bring it, you know where it came from. Because here, at the butcher, what they said last time is that they don't really know where it's coming from. It's here at the butcher, but they can't trace it back to where came from.

**Female Participant:** At the butcher?

**Interviewer:** Yes.

**Female Participant:** We don't have it here in the butcher.

**Male Participant:** We don't have it here. Not the butcher but the person who sells it.

**Male Participant:** The seller of meat, is a butcher.

**Interviewer:** They said you can go see and then he said, and then Kwasi said, "You can't go by yourself. Nobody's going to sell you anything."

**Female Participant:** They're not going to show it to you. When you ask they will tell you, "We don't have meat."

**Interviewer:** l should taste it. I would like to taste it but it's hard. I'll find a way. Kwasi will help me.

**Male Participant:** Their **[unintelligible 00:56:31]** Even here or in Ghana, you don't know where it's coming from. You only just meet it in the market, the person selling it. To ask of the source I don't think you **[unintelligible 00:56:42]**

**Male Participant:** Access me if you want me to buy you then I know where it's coming from. Because I know a hunter who--

**Male Participant:** Who can sell it to you.

**Male Participant:** Just sharing the deer.

**Male Participant:** Yes, because here nobody dries akaranti here. We can't say we have a source here. Even the dry fish here. The dry fish they are selling here. There are some sources which say that most of those dry fish are not really dry. They use something to paint them.

**Female Participant:** What?

**Male Participant:** Yes, there is a color. Those dry fish they have here. From **[unintelligible 00:57:17]** they have combination **[unintelligible 00:57:18]** now where they put some color or something on it. They put for some way a little bit of heat or something, they become brown as if it has been dried.

**Male Participant:** The fish?

**Male Participant:** The fish.

**Female Participant:** It doesn't taste good as our own fish.

**Male Participant:** Even the dry fish--

**Interviewer:** You can tell, can't you? At that level, nobody can trick you because you know.

**Male Participant:** We know the taste.

**Interviewer:** You're going to say, I'm not going to buy this one."

**Male Participant:** This is the real one. The sources, we can't get the sources. We only buy them when we have them.

**Interviewer:** Is it socioeconomic, is it a status symbol to be able to buy a certain kind? I always thought that-so bushmeat is anything from -- Now you're going to educate me. Now, you have to educate me, because in the literature, that bush meat is anything from like the lizards all the way up to the gorillas. It's that wide a range. Then as you have more money, you can afford to eat higher up the food chain. Also culturally, in three papers it said that also some people believe that they can get the energy from the animal as you go up the food chain.

**Male Participant:** I don't think there's anything like that in bush meat, no. We don't have stages a hierarchy in species. It's just from the rodent to the gorillas, all the same. It depends on who can buy it. If you can afford.

**Female Participant:** Do Ghanaians eat gorilla too?

**Male Participant:** We have some of them dried, even elephants.

**Interviewer:** The trunk, right? The trunks in the elephant-- [crosstalk]

**Male Participant:** I don't **[unintelligible 00:58:57]** trunk, the elephant. Even the meat itself, they can judge **[unintelligible 00:59:03]**

**Interviewer:** I heard that the trunk is the best.

**Male Participant:** I've not seen that.

**Interviewer:** I heard that the trunk of the elephant was the tastiest part.

**Male Participant:** I've not tasted that before, but I can't find anywhere. I don't know. Mostly, the bushmeat becomes every meat dried. That's the general name for it, we don't have specific--

**Kwasi:** Shall we make it central.

**Male Participant:** Yes, so that she really understands. The bushmeat, when we say bushmeat, it is everything from the [foreign language] to the elephant dried.

**Female Participant:** What is [foreign language]?

**Female Participant:** She doesn't know.

**Male Participant:** The lizard.

**Interviewer:** The lizards.

**Female Participant:** We don't eat lizards.

**Male Participant:** You don't eat but some people eat lizard.

**Female Participant:** Recently, I saw it on WhatsApp. Somebody was **[unintelligible 00:59:56]**

**Kwasi:** In Ghana?

**Male Participant:** Not in Ghana but I think in Asia.

**Kwasi:** We're talking about Ghana.

**Male Participant:** **[unintelligible 01:00:01]** eating from.

**Male Participant:** From the **[unintelligible 01:00:06],** that is the rats up to the elephants. Everything dry becomes the bushmeat.

**Interviewer:** Okay, I understand. If it's dried it's bushmeat?

**Male Participant:** Yes.

**Interviewer:** Okay.

**Male Participant:** All the time. [crossatalk]

**Male Participant:** **[unintelligible 01:00:21]** dried?

**Male Participant:** Let's look at this, this way. We have anything coming from the sea that's fish. We have got whales as well but whale can be Empunam because it is big. You cant eat it all at once. So you preserve it. That becomes impunam. Anything in the bush is meat. It's all meat. Be it a lizard, be it whatever, they are all from the bush. So it's bushmeat.

**Male Participant:** What is the difference between impunam and bushmeat?

**Male Participant:** Empunam is when the bushmeat has been roasted and preserved for so long a time. It becomes Empunam. The same thing is for fish. When the smaller fish that we eat straight away or you go get these crab meat, **[unintelligible 01:01:16]** the brown ones, sometimes they only used to be good meat. The **[unintelligible 01:01:22]** the big ones. The only thing was the preserved it for a longer time. It becomes more seasonal. [crosstalk].

**Female Participant:** The last time someone said, I think in the first group, someone said impunam was different types of bushmeat dried together.

**Male Participant:** That's what I know.

**Female Participant:** Nana is also saying different things.

**Male Participant:** As far as I'm concerned, that's what is the difference between impunam and bushmeat.

**Female Participant:** That's what you said.

**Male Participant:** So long as--[crosstalk]

**Male Participant:** I said it's all with respect to bushmeat is the one from the forest and not-- [crosstalk]

**Male Participant:** From the sea.Yes. [crosstalk]

**Female Participant:** Let me add something. Leave the guns. If my mother says go and buy punam or people come with the dried fish and they're saying it's impunam. That's how I know about impunam. I don't know about the bushmeat. I don't know anything. [crosstalk]. I thought the fish was impunam. Every dried fish is impunam. That's what they know. [crosstalk].

**Male Participant:** 'Impu' is smoked. [crosstalk].

**Male Participant:** Impunam means anything which has 'impu', which has been smoked. [crosstalk]. Impu means smoked.

**Female Participant:** So smoked fish. Impunam.

**Male Participant:** Yes, impunam means--

**Female Participant:** **[unintelligible 01:03:00].** What's the meaning of that. [crosstalk].

**Male Participant:** Impunam means to smoke a fish.

**Male Participant:** **[unintelligible 01:03:16].**

**Male Participant:** Here we know that impunam is every smoked meat. [crosstalk].

**Interviewer:** That makes perfect sense. It's obviously different species in different countries like Cameroon and DRC, in the Congo where there's maybe more forest. I mean a snake. This would make sense.

**Male Participant:** There also, they do it the same as we are doing here because they take, they preserve it. There's no fridge. They have to find a way of preserving it. Some people even dig a whole and they put it there after smoking it. They put it there for a longer time. Once a while they just go in and dig-- It will just work.

**Male Participant:** My initial question that I was asking from the-- Look at the matter of different types of [crosstalk] meat. Would you see them as the same? I would say no because I will only eat **[unintelligible 01:04:31].** I used to, but I won't. I prefer to eat probably **[unintelligible 01:04:37].** I can't take **[unintelligible 01:04:38]** because of the chemical.[crosstalk]. There're differences.

**Male Participant:** It all depends. There are smaller ones that could be tricked into these chemicals. Can use that to track them and catch them. The one deer that you're talking about. Deer is this very smart and all deep into the bush. That one, you need a hunter to go and get it for you. That one is more organic than those closer to the house. Those are the things that we have observed. It's that range there. Somebody will **[unintelligible 01:05:15].** Somebody is watching. Oh wow, this is something [crosstalk]. Something that is more organic than **[unintelligible 01:05:24].** That is the tricky part of it. **[unintelligible 01:05:36]** or whatever. You see them live. Live and you're standing there, you get it, you put it in your car, you go home and then do the whole thing.

**Female Participant:** That is the same thing you find with **[unintelligible 01:05:50].** You can also find **[unintelligible 01:05:53]** fresh on the roadside. You buy and you smoke it. I like it. [crosstalk]. I don't like the dried one.

**Male Participant:** A fresh one also tastes good.

**Male Participant:** I'm always suspicious. If it is along the road and they are holding it. It's going to bite. I'll be taking 50 pictures for the US and sending it to people on the road holding it like this.

**Male Participant:** Also, what you said is right. You have to know where to buy because I can't buy it in a crowd. Someone will, I can say in a crowd.

**Female Participant:** Maybe, you just bought it from the-- [crosstalk].

**Male Participant:** You think you--

**Male Participant:** I can't think far. [laughs].

**Male Participant:** You can't think far.

**Male Participant:** It's like the **[unintelligible 01:06:50]** something-- [crosstalk].

**Male Participant:** In a crowd.

**Male Participant:** From the source. [crosstalk].

**Male Participant:** Along the road in the **[unintelligible 01:06:59]** road. [crosstalk] you see people holding--

**Male Participant:** I'm from my hometown up to **[unintelligible 01:07:07].** [crosstalk]. Like if I want to buy from-- If I was not a **[unintelligible 01:07:16]** woman, I won't buy it from-- [crosstalk]. That was me. I to buy it from **[unintelligible 01:07:22]**

**Male Participant:** You can buy it from the shopping **[unintelligible 01:07:27].**

**Male Participant:** No, no, no. I was buying it from the Muslims and I **[unintelligible 01:07:30].** I'll have to go to them.

**Male Participant:** Yes, go to **[unintelligible 01:07:32]** and do the thing.

**Male Participant:** Yes. Then I'll know it's from the source. So you are right.

**Interviewer:** I think that makes--

**Male Participant:** Yes, that is me.

**Interviewer:** Yes. There was a man I can't remember his name now but it'll come to me, who was saying that he was eating and then he almost broke a tooth on the--

**Male Participant:** Yes. **[unintelligible 01:07:53]** and showed me the gun. The bullets are in it. That one it happens a lot. [crosstalk]. You know that it has been shot.

**Interviewer:** That's wild. That's a wild animal. That's the same as I went to a nice restaurant called the Black Line in Amsterdam. We ate **[unintelligible 01:08:14],** we ate duck. I'm eating and it's it was my husband's birthday. We had a really nice wine. Really expensive restaurant. I went [breaking sound]. I was convinced I broke my tooth. I was like, "I have no tooth. It's got to be gone." It was like Khh and I went ting into the plate. I said, "Excuse me." Then he brought me the menu and at the top, it said a message. I didn't read it. It said, "Sometimes on rare occasions, you can have the **[unintelligible 01:08:51]** because we are killing wilds. It's the best quality." It was interesting. I think I--

**Female Participant:** If you had broken a tooth, what would have happened?

**Interviewer:** I don't know. I don't have any idea. It would have been my fault because I wasn't careful and I didn't read the menu and it was right there.

**Male Participant:** I lived in Romania. I know that the gangs, will go and kill them and they don't hang them for a long time. They won't do anything. They wait for it to freeze, to probably rot and then finally becomes a base meat. They do it. Meat is different from-- Like Nana was saying when he went to Yugoslavia where they hang the **[unintelligible 01:09:57],** a deer. Most of the time there is pork and for under it dries very, very well and even in Nigeria. Let me tell you a story of Nigeria, the best meat you can get in Nigeria is the-

**Female Participant:** Snake.

**Male Participant:** No, the cow, the cow. They'll slice it and they dry it in the sun.

**Interviewer:** That must be very good that's like beef turkey.

**Male Participant:** That is so nice. It's just beef pepper, very dry, when you chew it, just like make chips.

**Interviewer:** See that's really interesting.

**Female Participant:** Where do they get the snakes from in France.

**Interviewer:** I don't know but they like to eat it. They like to eat snakes.

**Female Participant:** The Nigerians.

**Interviewer:** No the French. The French. The French eat things that-

**Male Participant:** The frogs. Even frogs. They eat frogs.

**Interviewer:** They eat-- Yes, we eat snails, we eat frogs.

[background conversation]

**Male Participant:** It might be awkward.

**Male Participant:** No they wouldn't mix it, they put from the menu.

**Interviewer:** When I was in Singapore. When I was in Singapore, we went to Malaysia almost every other weekend and so quick to go to the bridge to Malaysia and yes, on the menu there was dog and yes.

**Male Participant:** Hey, dog is a delicacy?

**Male Participant:** If you have it you have to bring it to the-

**Interviewer:** Yes. Our dog was still fat, our dog was fat. Our dog was really a fat dog and everytime in Singapore we got a Chinese friend they said, "Watch out for your dog, keep an eye on your dog because if you lose the dog, the dog is going to end up on a plate." I said, "Okay, though come here you steal to me but this is it." It's fascinating to me. It's fascinating because especially like the cultural attachment to the different food like we were saying the elephant trunk, the trunk of the elephant, they love, there are cultures that really love that part of the animal. Why that part instead of what you described here? I don't know. It's a taste. It's an acquired taste. It's what you're used to. If you grew up eating this thing you want it, but if you didn't grow up then at age 43 and some instance you want elephant and maybe not. Whereas if you grow up eating elephant then you actually miss it if you don't get it, because it has memories attached to it, it has a whole history. At least that's what it sounds like but I could be wrong since what an impression that I got.

**Male Participant:** I think it's true because, even our children when you start cooking the bush meat and the snails, they-

**Male Participant:** You see their faces they.

**Interviewer:** Then that's a good question. I'm part of that but then the children, the younger generation.

**Male Participant:** Our children?

**Interviewer:** Yes, your children and their children is the attachment to bush meat dying? Or is it dying here? Yes?

**Male Participant:** They don't know. It's dying because they wouldn't like to eat it.

**Interviewer:** When they get older does it come back?

**Male Participant:** No. No.

**Male Participant:** I don't think it is going to come back because they will never have it.

**Male Participant:** They are not old so.

**Interviewer:** Yes, when they get older sometimes you know how my daughter.

**Male Participant:** I hear, they come back.

**Interviewer:** It comes back?

**Male Participant:** Yes.

**Male Participant:** No, but there is a difference. Children who were born here and then children who grew a little bit in Ghana and came here. That's a big difference. Because my son was brought up there for when he was about 14, yes. It's the best meat. My daughter was born here now probably may not want to see of course, in her case I've brought her up to eat those things. If you have a home where a child has been born, is never been in to those bush meat and that children, they will-

**Interviewer:** Then he'll die. Yes then he'll die.

**Male Participant:** When they're going to remember the food, because they like Ghanaian food, they like jollof, they like rice and beans they like the red red. That is what they're going to take with them but not dried and bush meat.

**Male Participant:** Bush meat.

**Interviewer:** No. That make it interesting.

**Male Participant:** Any question which you think, the question is?

**Interviewer:** I'm just going with the flow. I'm just going with the flow. I've got questions but I'm not, it's not stupor

**Male Participant:** It's rigid.

**Interviewer:** Yes, I'm rigid and I have some questions asked that would only take 10 minutes to fill out and it's-- they're anonymous by the way like so. I don't need to know anything about your name but I also think that there is a lot that is valuable because like I said at the very beginning I personally don't, I didn't know very much but I don't even think that anybody outside of the African community knows very much about this food, this culture and everything that is fascinating especially when we talk about the organic side of it then it makes perfect sense because this is happening here at **[unintelligible 01:15:44]** where there is rose and rose if everybody trying to find organic to be healthier and it makes sense, right?

**Male Participant:** That's why again in this place where at our place, you see even when we eat lets say the food in the intestines, if you go they mix soup with the especially when there are grande and those enormous this thing in their stomach, the intestines

**Male Participant:** The shit

**Male Participant:** The shit in the intestines, we use them as food. People don't understand simply.

**Interviewer:** The green, what is the green and on the stomach that they put?

**Male Participant:** Yes, they eat only grasses. They eat only pure grass from the forest which is natural organic. Those grass it's easy for you to use them to make soup.

**Interviewer:** Yes, soup yes.

**Male Participant:** It's more like you're eating vegetables. That's how people they see it.

**Male Participant:** What about is a buffet stable food?

**Male Participant:** That is the, that is the.

**Male Participant:** The shit?

**Male Participant:** The shit in the intestine.

**Male Participant:** When it comes to the lower part, it's coming down there, the shit. Is the process of digesting.

**Interviewer:** It's the intestinal content.

**Male Participant:** It's intestinal content.

**Interviewer:** Yes, it's the same as an excrete, yes.

**Male Participant:** It' s not getting down the-

**Interviewer:** It still has all the nutrients. That's true. That nutrients and vitamins has not been separated from it. Okay.

**Male Participant:** They will eat those up to that level. When it reverses they then becomes-

**Male Participant:** Do you know that, Godiata was talking about the squirrel, I know that the squirrel don't take, you don't throw any part away.

**Interviewer:** The squirrel, yes.

**Male Participant:** When you catch it, you put on the fire nicely, you leave it then you just take it and put it on [foreign word] this and then you cut it the whole, everything you put it there, you tie it and then you put it in the soup and when it is cooked you take it then you grind it then you add it back. You don't throw any.

**Male Participant:** I didn't know that.

**Male Participant:** You didn't know that? Yes, you don't throw anything away.

**Male Participant:** Okay. You know it is one of the strongest animal, is very small but very strong, it can fight maybe because it is immune to snake bites.

**Interviewer:** What is immune to snake bites?

**Male Participant:** Squirrels.

**Interviewer:** The squirrels are immune to snake bites?

**Male Participant:** Yes. They are immune to snake bites. I watch wildlife at **[unintelligible 01:18:26]** a lot.

**Interviewer:** Even the really poisonous ones and?

**Male Participant:** Yes. They can withstand there they fight them.

**Interviewer:** They are so small.

**Male Participant:** Yes, they will never die.

**Male Participant:** I've never known.

**Male Participant:** You've never known? Maybe they've done is-

**Male Participant:** The wildlife you'll see all systems.

**Male Participant:** You just put the whole intestines in to the container and then you put onions and grind the bone.

**Male Participant:** You blend everything.

**Male Participant:** You blend everything.

**Male Participant:** I just put it back in the soup.

**Male Participant:** Back in to the soup.

**Male Participant:** Then in Americans, squirrels they come straight in to your house.

**Interviewer:** Yes, I had pet squirrels when I was a kid. Yes, I had Tom and Jerry. I had two squirrels and I named them Tom and Jerry.

**Male Participant:** Invite me to your place and I'll just.

[laughter]

**Interviewer:** One day they're there, the next day they're gone.

**Male Participant:** I was also thinking about, you see there is village somewhere in B. I don't know whether of you've been there, they have this black and white monkeys which live with the people. You've not been there before? Oh, the very popular village.

**Male Participant:** I've heard it.

**Male Participant:** I intend going there once to really look at it. Its been on TV, it lives with the people, the people live with them, apart from it, there is a history behind it.

**Male Participant:** Its because they don't eat them.

**Male Participant:** They dont eat them

**Male Participant:** They don't eat them.

**Interviewer:** What is your impression of then because obvious there's a lot in the media and the media does what the media does best with sensationalizing topics. So when they talk about Ebola and they talk about bush meat, and they talk about that connection and deep in the forest, when the logging companies they make their roads, then hunters can go deep, deep into the forest where usually hunters don't go because the there are populations.

Apparently, there are populations, I'm not a hematologist nor a conservationists but I have read that deep into some of the forest and obviously not gone probably but in other like maybe Cameroon or for Liberia wherever that with the roads, there are populations of primates that have been dying from Ebola for decades. 40 years but nobody knows because the forest is everybody's going deeper and deeper into the forest and that when you take it out and you butcher it, I'm telling you what I hear and then you tell me what's true and what's not true. Then that's where you can get the contamination. Is that is that your understanding of where this issue is coming from and how?

**Make Participant:** You see in some of these forests some of them are tsetse fly-infested.

**Interviewer:** Tsetse flies yes. They're the carriers because I read that there are two carriers, there's the bats, the fruit bats which apparently is very tasty, very, very tasty.

**Male Participant:** Apparently. [crosstalk] bat yes.

**Interviewer:** Apparently it's very tasty and but they are also one of the disease carriers and the other one is non-human primates. Any monkeys, gorillas and this kind because we share so much DNA. So then the diseases can be transmitted more easily. What's your understanding of that? Because very little is known. Very little is known, almost nothing is known about this connection and it's an interesting one and I think the University of Ghana is also studying it because they have their vaccine that they're now trying to use, you know the vaccine.

**Male Participant:** Well, I really don't know much about it, but I don't believe it because you see if it happens that those bush meat we are talking about if you consider how we kill or how we eat them. Even just as Nana said, we have cases where you go to the bush and you meet a dead animal over there, please don't throw them away, even sometimes you meet animals which is half dead, half rotten and you are not going to say that it has rotten so it's poisonous.

They will just bring that because the rotten is what is going to be the sweetest. You see the way we prepare our food, even as somebody said, my old grandmother, will see germs that is the germs. The germs in the animal. Once you have brought home [crosstalk] or well cooked the way we cook the food, there's no way you can get. If had that been the situation, our people would have died long time ago.

They go to farms, sometimes those animals those trapped from a good trap **[unintelligible 01:23:40]** mouse. You go there and maybe the trap has caught some animal. It has stayed there for a while and half is rotten but then they will bring them home and if you see the way they will prepare the food and how much time we use to cook our food. So the meat from our places are cooked in such a way that you--

**Interviewer:** Germs are killed.

**Male Participant:** Germs are killed.

**Interviewer:** They can't survive.

**Male Participant:** Our communities don't have half boiled-cooked food. We don't eat them, half-boiled meat, half-cooked.

**Interviewer:** Even smoked is all the way smoked through.

**Male Participant:** If you look at our pattern of food chain, you've never seen any food from us which is half-meat especially meat which is half-cooked.

**Male Participant:** What you are saying is true because the government can also have influence depending on information that Ebola issue it collapse a lot of these businesses in Ghana because people thought if you eat bush meat you might get Ebola. So they stopped totally so businesses collapsed.

**Interviewer:** So they mismanaged the message.

**Male Participant:** I don't know whether the mismanaged the--

**Male Participant:** Information sometimes we look at all these. Somebody has written something about you, said something about you but the reality is that people are using some excuses to sell their products. I mean when we talk of this Ebola, HIV/AIDS personally I don't have any interest in that because I see that there is an answer to that. We have answer to those things. Somebody this bushmeat living there years. Monkeys have been there, we've been chopping our monkeys and we now see ourselves being dying of HIV or Ebola but now all over sudden a year or two ago comes, Ebola ooh, Ebola ooh. It's a game thing to a certain extent.[crosstalk]

**Male Participant:** Ebola was a laboratory [crosstalk]

**Male Participant:** These are theories that are coming from other angles.

**Male Participant:** On the other hand, I've gone to Cameroonians and Gambians. The way they eat meat is not as us. Those communities apparently this things can have effect because if you go to like my neighbor is from Ivory Coast. If you go and they have this bushmeat on fire, if BA you will be you'll be surprised. In our case we organize barbecues but we don't have that meat these days. Its quite different and they eat a lot of meat, yes fresh ones and they are able to get in their country, if they don't have it they will other that [crosstalk]

**Interviewer:** There's always a way. If there's if a demand there's a supply. That's always going to be.

**Male Participant:** I think in our case, educating the public will have been, boil it well. They will say, Which meat?" [crosstalk]. Most of the shops will**[unintelligible 01:27:08]**

**Interviewer:** Did they come back though? Their businesses coming back? [crosstalk]

**Female Participant:** They have forgotten about Ebola.

**Male Participant:** Their problem is the way they describe such sickness, it shouldn't be a sickness which comes only one year and goes forever. There's no high pitched in a year and it comes in all of a sudden you don't hear within a year or two, if you have such a catastrophe sickness like Ebola and HIV it is not something you can even kill it in a year or two. That it goes completely and nobody cares about it, silently. Look at Malaria, Malaria has been there and everybody has been for years have been fighting it and it is still there. HIV and those sickness will just come in within a year or two **[unintelligible 01:28:02]** what happened? What miraculous medicine did you use to cure them?

**Interviewer:** Really then it sounds like it's in the butchering phase that you can get contaminated but nobody else can because you're boiling it near your sterilizing the meat. So it's really only the dangerous part in the butchering part where there's blood?

**Male Participant:** Maybe.

**Interviewer:** Maybe, but even then, with all the people that are been training there would be more cases. So then why some people get it and some people don't? Everybody I mean, the scale is so large that why only--

**Male Participant:** few people are just dying from them?

**Interviewer:** Is it one area? My understanding is that it's spread out so something must be happening where just it's a unique out of the big population. This is--

**Male Participant:** There only a few places that are just suffering.

**Interviewer:** It doesn't make sense if it is just beneath itself because not everybody would be the numbers will be larger.

**Male Participant:** Liberia for instance, I don't know the situation as at now, they were not eating bushmeat for all those years. So the Ghanaians who were there they were smoking and then transporting them to Ghana. They were enjoying it and they are still alive. All the countries, half of the Saharan depend on food from these animals. From Chad, Niger they have wildlife, lions and all those things. They eat horse. Their lands are not arab but they depend on these animals, that is why they are still very small.

**Interviewer:** That's their only source.

**Male Participant:** These tigers and all these, they have very powerful energy.

**Interviewer:** Yes. Okay, so that is an power energy that they're getting from the animals there.

**Male Participant:** Yes.

**Interviewer:** Yes. Nutrition.

**Male Participant:** Of course. If your organic protein come in from there, of course, definitely you'll see those **[unintelligible 01:30:23]** to be stronger.

**Male Participant:** If you eat a lion you would be strong--[laugh] [crosstalk]

**Male Participant:** If you go and buy a horse meat, for instance. It's very very expensive because it's -[crosstalk]

**Interviewer:** My mother believes that. Do you know that my mother believes this? This is not new. She said that when the mothers are in delivery, would lose a lot of blood and they became anaemic, she would give them horse blood. Horse blood in 48 hours, their iron was-

**Male Participant:** So high.

**Interviewer:** - so high. Like this, boom. Whereas.

**Male Participant:** Horse blood?

**Male Participant:** When you say horse blood, what do you mean?

**Interviewer:** The blood from the horse.

**Male Participant:** Intravenous or?

**Interviewer:** I dont know. In a little cup and the yes, she would warm it-

**Male Participant:** They drink it?

**Interviewer:** - and they would drink it but it's not that-

**Male Participant:** Horse blood.

**Interviewer:** - horse blood and they would make sausages out of it too.

**Male Participant:** You see?

**Interviewer:** The black sausages. It's blood sausages. Yes blood sausages and this is not only in France. This is all over the world, in Asia too they'll have different blood.

**Male Participant:** Okay for iron?

**Interviewer:** For iron nutrition.

**Male Participant:** I understand that the horse pee is also used in producing insulin.

**Interviewer:** Yes. This is why in global health, it is so important to understand nutrition. Different cultures are the key because everybody has their own opinion about-

**Male Participant:** What they eat.

**Interviewer:** - what they're eating.

**Male Participant:** What they are drinking.

**Interviewer:** They don't have all of the information. So they make their own story in their head about whether it's a good idea or bad idea with no information or not the complete picture at least. I think it's important because a lot of medicines to fight diseases and malnutrition and many things. Bonuses comes from traditional knowledge. Traditionally, you have to go back to what was the purest and what was the most organic.

I believe that but then again I'm just learning; I mean I'm a student. I'm not really allowed to have an opinion. [laughter] I'm not supposed to have an opinion. It's supposed to be neutral but I understand is what I'm trying to say.

**Male Participant:** Get your thing and that is it and then it's incident to the blocking of allowing yourself to find things yourself. I don't know, somebody was saying he went to Legon, University of Ghana and what the lecturer was producing in manuscripts is what people were buying it and this guy he bought one-- he didn't buy it, he went to the library & started researching. Did a good research for his work.

**Male Participant:** He will fail you.[crosstalk]

**Male Participant:** He failed two times and the father was angry. He said, "I've done everything" Then he was told, "My friend, go and buy it". He went and bought it and then he passed. You're not giving people the opportunity to search and research and do things themselves.

**Interviewer:** In America, there was a study, there was many studies and there was a study in the Liberian community and it was an amazing opportunity because, at the time it was just at the beginning of the Ebola crises. The Liberian children in Minnesota that were in school, when they had a temperature they sent the kids home immediately but then they contacted the entire school saying these Liberian children might have Ebola. It was a disaster.

This epidemiologist, she said, "Okay, we're going to do a study" and it was an opportunity to say exactly what is happening, why everything is happening and information because there was so much misinformation. I think, the same thing in Washington, DC, there was a focus group there but that was already like 10 years ago. There were student that said, "My grandmother when she comes, she brings me meat and I do much better on my exams". [laughS] It was just--

**Male Participant:** Weird.

**Interviewer:** Yes. It's just like taking vitamins for some people which is probably less healthy. Okay, well I think then the only other questions that I have are; and that's in the questionnaires. What's your favorite recipes? do you prefer your meat boiled? Or smoked? Or grilled? I brought pens this time. Remember last time I had only one pen and everybody had to wait.

**Male Participant:** You brought pen here.

**Interviewer:** Yes. I also think that it's very interesting that the younger generation here seems to not want to eat this meat because I know that growing up in the United States, I wanted to eat my mum's food and even though I never lived in France.

**Male Participant:** We didn't say that. Those younger ones who were brought up in Ghana, and living here a little bit. They would want to do it. That's the difference. So don't meet any young people like the group you saw, even our children, our party. Not all of them would hate bushmeat.

**Male Participant:** Yes, some would take it.

**Interviewer:** Yes because I never lived in France when I was growing up but my mum gave me the love of food. It was transmitted from my mother, that passion for food. My father has this whole history with the South, with their food colored greens and these things. I never felt any connection because he never was in the kitchen. It really is the mother that's passing it down and if the mother puts it on your plate and one, two, three times you say you don't like it. Well, four years later all of a sudden, if it's not on your plate, where is it? [laughs]

Now you want it. [laughter] I stopped eating some of those things but then it came back when I became a mother. When I became a mother, boom. All these things that I said I didn't like anymore, I didn't want to eat it anymore, it's not very nice to the animal, they'd all came back. It was like, "I'm French. I have a French passport. You're going to be French and it's through food and through the -- you're going to feel French".

**Male Participant:** This is part of being French.

**Interviewer:** As being a mother. I think also because they knew your-- Yes, you have to give them some roots. How are you supposed to give them roots if you're outside of-

**Male Participant:** History.

**Interviewer:** I think that food is probably a good way to stay connected.

**Male Participant:** If you were for example, this bushmeat we see that as a delicacy and it's the mother who is in the kitchen, who is preparing the food. When she prepares the food and you come and there's food there, you're eating [crosstalk]. As a matter of fact, when they see it lying down there straight away, they wouldn't touch it but when it's in the soup--

**Male Participant:** Even when it's in the soup and they ask what is this? [crosstalk]

**Male Participant:** You then tell them something else.

**Male Participant:** They enjoy it. [crosstalk]

**Male Participant:** It depends. At least, they would know what it is, but it takes-- Well, just like last time, last four years, we went to Ghana with our boys.

**Male Participant:** Yes. The goat.

**Male Participant:** They were young and somebody brought me goat. I kept the goat at some corner there-

**Male Participant:** They were playing with the goat.

**Male Participant:** - and they were playing with the goat and they were cutting grasses. They would go and cut grass from outside and come and feed the-

**Male Participant:** The goat.

**Male Participant:** -goat. One Sunday, we had to slaughter it. I was not there, I was outside.

**Male Participant:** They were in the room [crosstalk].

**Male Participant:** They were in the room and we sent somebody to go and slaughter them. When they were standing inside the house like this, you can see the yard of the house.

**Male Participant:** The fire.

**Male Participant:** The fire. No, the goats. When the guy woke up, he was just going to look if there's--

**Male Participant:** A small one there.

**Male Participant:** - the goat was there. When he looked there he saw, the guy tied the goat's leg and then cutting the neck. When he rush, he comes, "There's a murderer there" [laughter].

There's a murderer in the house. What's happening, he say, "No, he's killing the goat" Later, you realize that you took him and say, "Okay let's go for a walk before we come" So that they'll leave the room.So anytime they'll see the man comes to the house, they'll say," The murderer is coming"

[laughter]

Then he later when-- [crosstalk] we thought them here it's normal, that's how we do it. Here we slaughter it and now they understand it now. We don't let them see it, they're doing the slaughter but then when it's in the food they like it.

**Interviewer:** Yes, but that's another thing.

**Male Participant:** It could be a trauma?

**Male Participant:** Yes. Yes.

**Male Participant:** I've experienced that too.

**Male Participant:** Yes. With your children?

**Male Participant:** Yes, with my daughter.

**Male Participant:** Here is plenty. That's how it changed normally in society in Zaire. Then they understand, okay.

[crosstalk]

**Male Participant:** Hello.

[crosstalk]

**Interviewer:** I think it's true that we are very far removed from the animals. You go to the store, it's already prepackaged, and then you put it on your plate and for children, they don't see the connection between the wild-- [crosstalk] When they see that, it's shocking. They're not hunters.

**Male Participant:** Okay. No more questions?

**Interviewer:** No more questions. There were no questions, it was just a discussion. I'm so grateful for this opportunity. I have my surveys because I have to wait and put it all into the computer. This time my daughter didn't spill coffee all over them like last time, remember? My daughter, she spilt it. You already took it? Then I give you a pen, here you go.

**Male Participant:** That's a nice pen.

**Interviewer:** Yes. I got it from the hotel. You can keep it if you want it. I have like four of them. [chuckles] Thank you. Do you have a pen? I have many.

[background noise]

**Male Participant:** [Foreign language]

[laughter]

**Interviewer:** Do you want to fill one out? No?

[Foreign language]

**Male Participant:** [Foreign language] What is it? I don't understand it.

**Male Participant:** [Foreign language]

**Male Participant:** [Foreign language] is it far from the courts?

[Foreign language]

**Interviewer:** [Foreign language]

**Male Participant:** Okay. This is a different ball game altogether.

[Foreign language]

**[01:46:55] [END OF AUDIO]**
