# Supplementary material for: Exploring the characteristics of a local demand for African wild meat: A focus group study of long-term Ghanaian residents in the Netherlands
Source: PLoS One. 2021 Feb 16;16(2):e0246868. doi: 10.1371/journal.pone.0246868 (PMC7886224; doi:10.1371/journal.pone.0246868)
Supplement: S3 File — (DOCX) [file pone.0246868.s007.docx]

**Africa Focus Group 3**

**Coding key**

| **Pink** | **Luxury Item/Status** |
| --- | --- |
| **D.Blue** | **Willingness to Pay (WTP)/ Bushmeat Price Comparison - Ghana vs. Netherlands** |
| **Yellow** | **Food Preparation/Taste** |
| **Green** | **How to avoid infection / Health considerations** |
| **Purple** | **Generation changes in bushmeat consumption** |
| **Red** | **Species** |
| **Orange** | **Local Demand & Trade**/ **Methods of Acquiring meat locally** |
| **L.blue** | **Methods of hunting** |

******Notes on body language, gesturing, tone and volume have been made in blue italics transferred from notes taken by the interviewer during the discussions.******

**================================================================**

**Participant:** I can’t cook it for you. I don’t eat meat [laughs].

**Participant:** My work is very close to the--

**Moderator:** The forest.

**Participant:** They have an area in **[unintelligible 00:00:16]**. During the winter, they have all these wild horses and wild deer and wild-- Because they are not able to get food, they really kill them, shoot them and destroy them. I was looking for a way to get into the people so that I can export them to Ghana especially the deers and this.

**Participant:** That’s a good business.

**Participant:** Yes. I got to a point--

**Moderator:** Very good, because they eat meat.

**Participant:** I got to a point I couldn’t go further.

**Participant:** I ask myself sometimes. Look at the house. They’ve got a big one.

**Participant:** Family, yes.

**Moderator:** All these animals that are just walking there, grazing, enjoying the ecology of the place, they are rich in all the--

**Participant:** The vitamins and minerals.

**Moderator:** Do you know micro?

**Participant:** I know micro.

**Participant:** Yes, micro.

**Participant:** Getting to Christmas, go to their department Vilt.

**Participant:** Vilt.

**Participant:** If you go to Vilt, you will see the type of bushmeat they sell there getting to Christmas.

**Participant:** Christmas?

**Participant:** Yes, this coming Christmas, wild pig--

**Participant:** Wild boar.

**Participant:** Yes.

**Participant:** We eat that in France too. Sanglier we call it.

**Moderator:** That’s a bushmeat. They have got the **[unintelligible 00:01:44]** and rabbits in England?

**Participant:** Yes.

**Participant:** Not the ones that are walking around here?

**Participant:** What we have in Africa, do they bring it there?

**Participant:** No.

[crosstalk]

**Participant:** They have their own.

**Participant:** Okay.

[crosstalk]

**Participant:** For their own consumption.

**Participant:** The popular one increase when they are pheasant. They kill them a lot.

**Participant:** Do they have the same taste as the--

**Participant:** No.

**Participant:** Well, you see--

**Moderator:** No, they don’t have the same taste.

**Participant:** It all depends on how they preserve it.

**Participant:** Preserve it.

**Participant:** They are able to preserve the meat themselves. The wild rabbit, if you send the meat, they think that this is not from the regular, because the way they eat from the bush they see that it’s--

**Participant:** I think we talked about--

**Participant:** He is an expert in cooking.

[laughter]

He likes cooking a lot.

**Participant:** We touched on that a little bit last week.

**Participant:** No, I’m serious. He likes cooking a lot.

**Participant:** You do?

**Participant:** Yes.

**Participant:** Last week, they said that men weren’t going to the market as often as the women, because the women were the ones that were cooking. Then there was a joke. My husband cooks.

**Participant:** He cooks.

[crosstalk]

**Participant:** I’ve got a term in Ghana. It’s man’s soup. If you go there, it’s only the men who are cooking.

[laughter]

**Participant:** Hello.

**Moderator:** Maybe, in Amsterdam, probably it’s not popular. When you go places like **[unintelligible 00:03:23]** and all those places, it’s normal in the shops. They have it in their shops. Only here, you see, we don’t have this-- [crosstalk]

**Participant:** My dear, how are you?

**Participant:** I’m fine.

**Participant:** I heard your mother died. Sorry.

**Participant:** Thank you very much. You’ve remembered me.

[laughter]

[background conversation]

**Participant:** Hello. How are you?

**Participant:** Good to see you.m[crosstalk] What are you saying?

**Participant:** Chairman, why don’t you agree?

**Participant:** Why should I agree?

**Participant:** It belongs to all of us.

**Participant:** Have a seat. [crosstalk]

**Participant:** It will be 20.

**Moderator:** Kwaku, sit down. Unless the Female Participant, the coordinator--

**Participant:** I think we’re looking at it. Also, that lady will be coming.

**Participant:** That’s what she was saying. We can begin.

**Moderator:** The message she sent to me it was not concrete. I don’t want us to waste more time.

**Participant:** Then I'll propose proposal that we eat just as we did the other time?

**Participant:** Yes.

**Participant:** I wrote our proposal we eat. And then--

**Participant:** Count it. [crosstalk] Ella, it is expensive. [crosstalk]

**Participant:** I’m listening to the conversation [laughs]. [crosstalk] Can you help me?

**Participant:** Please, let’s centralize. Opoku, you guys are--

**Participant:** I told you to come and stop them. [crosstalk]

**Participant:** Your second what? You’re eating.

**Participant:** Yes, because I was--

**Participant:** Reinforcing the--

**Moderator:** No. Maybe your mind is going high.

**Participant:** We will call you.

**Participant:** I went--

**Participant:** Can I go and repeat?

[crosstalk]

[laughter]

**Participant:** Last time, I think the group was saying that the way of hunting is changing sometimes and many times in three groups. Somebody has mentioned that it’s surprising to see poison, surprising to see different ways of hunting changed. The way of hunting might be changing. That’s why I was wondering, I know that Adansi has had an experience where he says, “I’m not going to go back to that area to eat that because he didn’t feel well.” That’s not something that you’ve experienced.

You know where it’s coming from, the area generally. Just to repeat so that I’m clear. You just feel it’s safe because that’s an area that you haven’t had any experience with the poisoning.

**Participant:** It’s a mountainous area.

**Participant:** With the poison being on the ground, the poison wouldn’t be necessarily in the trees.

**Participant:** Exactly.

**Participant:** Well, is that what you were saying? Maybe, there are other animals that can get poisoned. Maybe bats are hiding in the trees. They wouldn’t-- [crosstalk]

**Participant:** Mind you, bats have a very poisonous bacteria infection. Sometimes, they will be wild. If they scratch you, if you don’t take care you will go. All these things are known.

**Participant:** I want to ask doc a question.

**Participant:** All these things are known by the local people. Our fathers and mothers did they know this?

**Participant:** Yes.

**Participant:** They will only eat it when they know that it’s well--

[crosstalk]

**Moderator:** Do you know that our traditional medicine according to **[unintelligible 00:07:54]** fighting and go to that place and give it to the animals. That is why they’ve grown to know the leaves that are conducive for our health. You can eat any leaves just like that. Old people at that time in their tradition, they go in the night and watch how the animals were living and what they were eating.

**Participant:** Yes, they studied them.

**Participant:** They studied and they will go to pluck that leaves and also to use as medicine on their patient.

**Participant:** I want to ask doc a question here. Doc, you remember the days of Ebola? Even till today, research is still going on. Okay, it was definitely put to the world scene that it is bat which is a 100% carrier of the disease. All bats around the world--

**Moderator:** No, it was not the bat.

**Participant:** That is what we heard after this. I want to ask you, how were they able to quarantine these animals because in my area they had it. We didn’t have even one casualty from the research till to date. I can show you our restaurants. I went to Ghana. Quite recently I came. In my hometown, we have a special chop bar. Before you enter my hometown, it is the only chop bar there. You see the building, nobody is standing there. People are eating and there is nothing. I want to ask, “Were they able to quarantine the animals that we are eating? Why are we still not getting it?”

**Moderator:** Getting what?

**Participant:** Ebola.

**Moderator:** Who tells you Ghana has never had Ebola.

**Participant:** No.

**Moderator:** The cases were very few. If you were in Ghana at the time of Ebola, how many, what do you call it? Drop bars where we're closing down.

**Participant:** Can you record the cases? We want to see the records.

[background noise]

**Participant:** In Congo **[unintelligible 00:10:38]**

**Participant:** In Congo? You finished?[crosstalk]

**Participant:** Because of the comfort I think that they discovered there then they're producing there.

**Participant:** What have they found in Ghana because there's gold?

[background noise]

**Participant:** But what Dan was saying indeed they had-- After the report, there was a research on that bat that you're talking about. Indeed some of the bats were infected by Ebola. The research says that they migrated from those affected countries to Ghana. That is why they closed most of the shops. Then those bats before the **[inaudible 00:11:30]** two had already got the disease. We were consuming them. How many Ebola cases did we record if you can find out?

**Moderator:** For Ghana, I can tell you none. Why? Because our style of cooking is different from Europeans. [crosstalk]

**Participant:** I was telling a story. My neighbors here in The Netherlands are from Ivory Coast. If there's summer and you go there and they're eating their beer and their barbecuing is different from the way we do. The meat they eat imported bush meat, you'll be surprised.

**Participant:** One thing about Accra is when I was going to school here **[inaudible 00:12:26]** he came to school and told us that **[unintelligible 00:12:28]**. He told us that he discovered with homosexuals. **[unintelligible 00:12:36]** tradition came from Africa.

[laughter]

**Participant:** There is the whole issue of sex. I've worked in this HIV/AIDS for more than 19 years. I worked continuous. The issue was that the first research which was done in the Netherlands. [crosstalk]

**Participant:** It didn't change. It didn't scare anybody. Is that what you're saying because people just kept eating the bats? Or they stopped eating the bats?

**Participant:** Its beliefs. Well, they're still having the believe what we have is confined in our area where we are living in. From our belief thought we know they are eating from their right source. Under no circumstance will they contract any disease from anywhere. That is why the market still booms because at a given time the girls were watching whoever would shoot any bat. It raises when the man is prime.

**Moderator:** To be honest with you during the Ebola era in West Africa you wouldn't offer bush meat to anybody to who would accept it. It was a totally undone. [crosstalk] I was in Ghana. If you come to my area there is what we call-- There is a restaurant. They call it "Dirty Soup." Dirty soup is all types of bush meat.

**Participant:** We call it remix soup.

[laughter]

**Moderator:** No. The name of the restaurant is called Dirty Soup. Every type of bushmeat. It collapsed. Nobody was going there.

**Participant:** Did it come back?

**Participant:** Now they've come back. [crosstalk] No. It's the truth.

**Participant:** There is only one place.

**Moderator:** Government has assured that Ebola is no more spreading. Ebola has been addressed. Ebola in Liberia and other countries has been addressed.

**Participant:** How did they do it?

**Participant:** Let me give Doc one place. [laughs] Let me give Doc only one place.

**Participant:** [crosstalk] From the government. They own the media, they have an agency, EPA, FDA.

**Participant:** She's going to Ghana. I'm giving you only one place in Accra. It's only bush meat and it's very popular. Twelve O'clock you won't get a place to park your car.

**Moderator:** Which one? What's the name?

**Participant:** From **[unintelligible 00:15:54]** waterways. Where we used to buy water. Where the pond water is. You see the tankers. From there you go to the atomic dancing. The runabout.

[background noise]

I know the woman. That is where I eat.

**Participant:** You will prepare me this **[unintelligible 00:16:07]** soup.

**Participant:** I met the woman. I will call the woman. You'll be there they'll be bringing the **[unintelligible 00:16:16]** because I live in the area. I live in West Lagos.

**Participant:** **[unintelligible 00:16:23]**.

**Participant:** Before and after we were eating. The woman was preparing for us. Everybody is happy.

[background noise]

Not too much people were coming. We have the bush pig.

**Moderator:** Whiskered.

**Participant:** Whiskered. They have a special soup for. You see they couldn't make it light. You lived right. They go all these **[unintelligible 00:16:54]** on Instagram. We were eating meat.

**Participant:** It never stopped?

**Participant:** No. It stopped.

**Moderator:** No, it stopped. It just stopped. You said it.

**Participant:** They were making **[unintelligible 00:17:05]**. I was there myself. **[unintelligible 00:17:08]** I'm telling you Doc. [crosstalk] That place. I was eating there **[unintelligible 00:17:20]** that time. The woman is always special for bush meat. She was selling. She was telling the people, "Leave those people who are making their research." There was no sickness.

**Participant:** I have a different experience.

**Participant:** Doc, please give them the address.

**Moderator:** Since then I've never been-- In fact the last time I'm getting some I don't know because I was going there very often when I went to **[unintelligible 00:17:51]**.

[crosstalk]

I don't go there because immediately you come in there is a sense.

[laughter]

I go to the other one.

**Participant:** Help me do. You stopped going there.

**Moderator:** No **[unintelligible 00:18:23]** has changed hands.

[crosstalk]

**Participant:** Which ones? Where is it?

[crosstalk]

**Participant:** **[unintelligible 00:18:53]**.

**Moderator:** Is my area home.

**Participant:**  We'll give you title.

[laughter]

The place there is a very big place. Like Saturday and Sunday.

**Moderator:** You see I'm going to give you an example of the bats. We had around 37 hospitals. That was the area where let's say the bats in Accra [crosstalk].

**Participant:** Near the hospital?

**Moderator:** Near the hospital. If for example, they're talking about Ebola spreading or being contaminated or being infested with Ebola, I would have said that because of the locality and the population's concentration in that environment this is the one in the farm and mountainous area. Of courses they do travel way back there, just look at the eagle. When the eagle they will go to the place where they can go and shed their wats. They re-energize, so the bird from Accra can go all the way to the north and decide, "Oh I have come to my area and recuperate myself and go back." So, those ones who cannot be able to travel back there can even be infected with Ebola but even that people were enjoying [crosstalk].

**Participant:** I think we can start now, the formal, this was introductory too but we will be very happy if Doc will show you all those places where he has been to but is also [cross talk]

[laughter]

Okay, let's start the program.

**Participant:** I have to get some soap, I am just going to get some soap.

**Participant:** Very interesting [crosstalk]

**Participant:** I think this stains so I don't want it to stain red on the white. This is very interesting for me. Many of you know me already except for those of you who don't, I'm studying nutrition and global health and culture. So I'm interested in bushmeat, why people eat it, what they like about it, what their concerns are, how they prepare it and we had really nice conversations over the past several months and I work at Utrecht University Medical Center and I'm a first year Ph.D. student.

This is just the beginning for me and some of the highlights that I thought were the most interesting is that not very many people understand bush meat and outside of people who eat bushmeat. So they have their own ideas about why people eat bush meat, what is bushmeat, what species do people like the most, the least and how much money does it cost?

Is it expensive and does it taste the same here as it does when you eat it back in Ghana? We laughed because I don't think that my wine when I drink wine in France tastes the same when I drink French wine here. So the last thing I think was that I think I covered everything actually, I can't think of anything. Those are the topics, the general topics and the bats we hadn't talked about. We talked about a lot of species but not bats but I have read that people love eating bats and I didn't know anybody that ate them so you are my first and I appreciate that. I understand that there are concerns about bats them being reservoirs of disease but at the same time, you also told me that they are prepared by smoking but not by boiling.

**Participant:** No, smoking.

**Participant:** It has a strong flavor.

**Participant:** Yes a strong flavor.

**Participant:** So a little bit can change the whole soup?

**Participant:** Exactly.

**Participant:** Okay because that what I learned last time is that you just need a little bit of the meat and it changes the whole soup.

**Participant:** You know it's in the flavor, just to stimulate your saliva.

[laughter]

**Participant:** We've also had a gentleman who is here, who taught me a lot who is doing his Ph.D. in Leiden and he is doing a study on bushmeat and infertility. How it can help with infertility which I'm fascinated about seeing his research when he is finished because it is probably going to be amazing. I have not seen anything like that so he will be pushing forward knowledge for people that can't make that connection. I think that that's what this is about, it's about making connections between nutrition, health, and culture. So we talked about--So it's funny because obviously, I go to Albert Hein and more and more are used to be marked but now in Albert Hein, I see biologic, biologic, organic.

Everybody seems to understand that that is probably a healthier way to go. I don't know if I trust all the packaging that says biologic but when you talk to me about meat from the wild, that does sound biologic and organic to me and it does make sense. Like you were saying that is leaner and there's less fat, the animals get more exercise, they're eating things that are in the wild so their bodies are healthier and so when we ingest it that bioaccumulation of good minerals and vitamins makes sense to me too.

I think that am learning about the different kinds of meat, stale Punam was a big conversation. That was a big conversation, it took me hours when I left and I went home and I listened to the tape again and I wrote down Punam, I thought everybody had the same definition of Punam but not everybody has the same definition of Putnam. So now I understand it better I thank you very much.

**Participant:** It was part of the vetting process.

**Participant:** Yes because if I think am saying Punam is this and other people think that's not Putnam, this is Punam, do you remember that conversation?

**Participant:** Yes.

**Participant:** I was so confused. I had to listen to the tape so many times to try to understand.

**Participant:** If I may break in, sorry to break in, in my place-

**Moderator:** No, allow her to-

**Participant:** That's it, am done.

**Moderator:** I want you to chronologically go to your questions and then we can answer them.

**Participant:** Sure, I guess the first thing is would you say some of the other groups have different answers but would you say that the younger generation of Ghanaians in The Netherlands are as interested in bushmeat as the older generations?

**Participant:** She says no and why?

**Participant:** The reason is when we cook our local food the children they don't like it. They will say, "Mm IT smells, it stinks and so forth." So they don't even know what it is. What the Punam and the bushmeat or whatever, they don't even know what it is. So I don't think they like it. We were born there, we were grown there. So they don't even know what it is.

**Participant:** Is it possible that when you start eating it at a very young age, I know Adante had a very good example of his son who has already spent the earlier years. So then by the time he got here, he already had a taste for it but then maybe you don't eat bushmeat enough that it's becoming a habit at a young age. It's once in a while, right?

**Participant:** Yes, one in a while.

**Participant:** So then for them, they can easily say no while as if it was every day they wouldn't say no? Is that right?

**Participant:** Yes.

**Moderator:** I wanted to push something by what she is saying, here in Holland because it depends on the parents. Not all parents are fanatic for bushmeat and financially bushmeat is a little bit expensive. So if you make your budget like most my people they say they are preparing, you know what preparing is? They budget what they have to pay or their rent and everything and if they make their budget, they have to keep this money, the money has to be kept irrespective of your list of items you have to buy.

So going to the extent of buying the bushmeat you are trying to bring extra cost to your monthly day to day administration. So most parents choose even not to go there but when you go back home, they take you on holidays, they introduce those things to you. So coming to the children if you have been introduced to patat and all those things you will stick to patat and it is easy to put in the oil and pack it and eat it.

Coming to the topic we are talking about, bushmeat. When they are in school, they show them, it's only in Africa where we don't have videos of the animals and all those things but here when a researcher comes and you want to prove to you this and that like the mouse and the rat they are in the same family. So here they hate to see those animals. They prevent it, they prove to them these animals are dangerous. Bringing this rat from Ghana to your house, your child can call the authorities to come and pick you away, because papa and mama are going to eat something poisonous in the house.

**Participant:** That's the impression they get.

**Moderator:** Let me put that impression.

**Participant:** The impression. I see what you're saying. Okay.

**Moderator:** Who are you to bring all those animals?

**Participant:** I agree with you, but what I want you to know is, upbringing of children here is different from upbringing of children there [crosstalk].

**Participant:** Yes. I agree with both of you.

**Participant:** We're not the only people who are bringing our children off the schools, because they tell them they don't **[unintelligible 00:30:45]** they don't do that. When you come here, they question you because you train them to be assertive. Children here, they always ask questions.

**Participant:** Yes, that's true. I have four, and I can tell you I'm challenged every day.

**Participant:** They always ask question. You can explain bush meat, they say, "But, Ma, did you say that, and that, and that?"

**Moderator:** [laughs] Challenges you.

**Participant:** I think you reference Ghana a lot. Along the street, you see a grass cutter, "What is that?"

[laughter]

Most of them are also teachers. Can you imagine the information they bring back to Holland? Because these children, when they come back from holidays, they say, "Where did you go, what did you see?"

**Participant:** It's just a lot different. Yes.

**Participant:** What do you think they will tell? We saw bush meat along the road. What would they tell them?

**Participant:** They will tell them it's not good. They say it's rat.

**Participant:** That is true.

**Participant:** For me, I think when they tell me it's rat, I say it's not rat.

**Moderator:** My children have that problem, so they will always do that to me.

[crosstalk]

**Participant:** They don't want you to kill animal.

**Moderator:** Let's just centralize it. He says they will say it's rat. Of course, it is rat. It is rat.

**Participant:** No, it's not rat.

**Participant:** Grasscutter is not **[unintelligible 00:32:15].**

**Participant:** Then you have to explain it to them.

**Participant:** That is what I'm saying, but anything [crosstalk] they say no they're the same thing.

**Participant:** They don't take it.

**Participant:** I'm confronted with it. I've been confronted [crosstalk].

**Participant:** They say grass cutter is rat.

**Participant:** It's true though.

**Participant:** I've been confronted **[unintelligible 00:32:26].**

**Participant:** No, I [crosstalk].

**Participant:** **[unintelligible 00:32:29]** they're always there lined up. I will take [crosstalk].

**Participant:** You know it's the best place. You know it is.

**Participant:** At least it's my place.

**Participant:** Then if they say, it's true because it's a category. Species.

**Participant:** No, it's not rat. No, no rat. I mean, when you go to school, you will learn, this is rat. I don't think they have it in their syllables here, where they can show to you.

**Participant:** [crosstalk] in our country, this is an animal they don't see there. Although, these European they can do anything.

**Participant:** That is why I'm saying that it's not in their syllables.

**Participant:** They give name to everything.

**Participant:** They gave one name to everything.

**Participant:** To everything. They generalize it.

**Participant:** The grass cutters that we are talking about is in the family of rat. When they see it, they call it rat.

[crosstalk]

**Participant:** Buy meat or chicken here, is different from what we have in Ghana. This is the difference. In Ghana, you may have it in the fridge in the market. Even chicken out of the freezer on that plate in the market. You won't buy it. You will prefer going to the go slow and get them, because you might think that, "Okay, the one in the cooler is better than the one in the market".

**Participant:** Of course, I won't buy it like that.

**Participant:** When you take those children from here to visit, they still [crosstalk].

[laughter]

**Moderator:** I will go to the market and by my food.

**Participant:** We grow up there, **[unintelligible 00:34:08]** before coming here, so we know how to prepare it.

**Participant:** It sounds like the children are just very far removed from the process of the animal to their plate.

**Participant:** If you take them from here to Ghana, you take them to a shopping mall, where they sell meat. Then they see that rat they're talking about. For example, chicken. They had chicken brass or freeze. No hands, no head. They have the feet, they have the shoulders, they have here, grouped. If we have the same there, back in Ghana, say grass cutter, and we have the breast, we have the parts from the grass cutter nicely presented in the cooler, you will love to taste it.

**Participant:** I want to ask you some questions. This country is surreal. This country, everything is a **[unintelligible 00:35:18].** You know **[unintelligible 00:35:19]?**

**Moderator:** Yes.

**Participant:** Everything is **[unintelligible 00:35:21],** everything they give them antibiotic injection. Injection that will protect them from everything. I know that they easily get sick when they go to where things are rough. They get sick easily. Our children, they get antibodies. They go to all rough system, so their body itself can challenge any disease because you're body has to challenge disease before you put external body.

**Participant:** What you're saying is very related to a big topic in global health right now, with the antibiotic resistance, antimicrobial resistance, and where you see it, you see **[unintelligible 00:36:04],** in hospitals. Why? Because it's still and it's perfect. It does sound like I understand your point there.

**Participant:** They love those places.

**Participant:** I wanted to bring [crosstalk].

**Moderator:** [crosstalk] The question goes in like, our children, why are they not enjoying our bush meat while we enjoy it? Is it because [crosstalk].

**Participant:** Children in Ghana enjoy it, children in [crosstalk].

**Participant:** He answered the question straight away.

**Participant:** They have so many foods here.

**Moderator:** Yes, but then, we don't also have them in large quantities here.

**Participant:** You were talking about cost. That I think that--

**Moderator:** Yes, the cost, but if the cost were removed, and if you bring them right **[unintelligible 00:36:52],** you think they will take them?

**Participant:** Yes, they will take them.

**Participant:** That's actually no. They won't.

**Participant:** Let me explain why. I say they will take it, so why? Let me explain.

**Participant:** No, they know [crosstalk].

**Participant:** [crosstalk] you're coming from Ghana. Take your meat, put it in your bag.

**Participant:** Let's assume that--

**Participant:** If it were in quantity, even Trump said they shouldn't take something from European country. The only thing they like from Ghana is cocoa.

**Participant:** Your point and your point are very interesting because you said it was cost prohibitive.

**Participant:** Cost prohibitive. That is one factor.

**Participant:** If it was less expensive, and you could eat it more often, then would your kids be more used to it?

**Participant:** I will say they will eat, because, let's think about this way. If the authorities allows that if they found that even it's very good for Holland people, or when they eat, they will be very stronger than the Africans, let me put it that way, or the black people. Will you think they will just put a ban on it? They won't put any ban. We have to have a way of marketing it. They are animals which are not always standing there for you to shoot, and kill them.

They are rare to get. How do you promote this type of meat? It's not easy, but if all things being equal, the authorities allow this. Like what he is saying, to take their head off. Take it from me, if a child of Holland, together with his friend, go together, and they see this hares like a monkey head, and they sell it as meat, which we also eat in Punam. You will never even talk to that your friend anymore because he is a devil. Seriously speaking. The way we will present it, and the way we will bring it to the market, like what he said, you don't see that heads and Punam isn't Punam. We just ask.

**Participant:** Yes, that's what we talked about. The monkey meat has yes.

**Participant:** Except whereby, the monkey is also common. Monkey meat is like a fiber.

**Participant:** It's the best.

**Participant:** It's the best. It's like fiber.

**Participant:** Yes, that is what I've heard.

**Participant:** When you dry it, on fire, you get the black soaking everything together, that is very strong. It makes you strong.

[crosstalk]

[laughter]

**Participant:** I eat monkey. That was the [crosstalk].

[laughter]

**Participant:** K-W-A-K-U-O. Kwakuo.

[crosstalk]

**Participant:** K-W-A-K-U-O.

**Participant:** My father's sister was the head of selling of [crosstalk] in my place.

[conversation in foreign language] [crosstalk]

**Participant:** They saying that we borrow but it's not true.

**Participant:** You don't eat gorilla?

[laughter]

**Participant:** Wait, wait, wait.

**Moderator:** It's enough.

**Participant:** Gorilla?

**Participant:** Is it expensive?

**Participant:** My father's sister was the **[unintelligible 00:40:29]** mother of this whole of this meat and everything.

**Participant:** Gorilla is expensive?

[crosstalk]

**Participant:** I want to bring in a conspiracy theory because I want to support that. Do you know, these European, they want to protect the animal because their reserves have been dead. So for their zoos, they don't want us to eat our bushmeat.

[crosstalk]

[laughter]

They keep more money to protect the animals. They are willing even to **[unintelligible 00:41:03]** it. We don't want to eat food from Europe. We want to do our own thing. We can get it easily, you see?

[laughter]

**Participant:** Badong, have you ever, in the research. Have you ever made a research of why do we patronize bushmeat than the one that we have been reared with?

**Participant:** I will tell you one thing, from my own viewing.

**Participant:** From research or you are talking general.

**Participant:** No, from research, I'm talking about first my experience. In Carribean for example, they hardly eat meat because the bushmeat, one way or the other they've all vanished from the forest. all that they eat is dried fish.

**Participant:** Not because they prefer that because there is no more bush meat?

**Participant:** No. It is not that they prefer. it is not that. When I was young---

**Participant:** What caused the animals to run away from the area?

**Participant:** When I was young, my father could go to the bush and he would hunt let's say, a monkey, he would hunt a**[unintelligible 00:42:11]** but these days if you want to get real bush meat,there are very few hunters. Previously we had alot of many hunters. They would go to the ---our place is very close to the mountains. They would go there in the night and if you were able to get the money to buy,then they would bring you.

[crosstalk]

**Participant:** Do you know why?

**Participant:** Why? it's because the farms have become more or less a visiting area everyday and animals don't want to see human beings.

[crosstalk]

**Participant:** Do you know what. At this rate that they're cutting our timbers. Animals, their food they are cutting our timber and bringing them to Europe. l've stayed there before, pathetic. All the timbers have been cut.

**Participant:** The logging companies?

**Participant:** Yes together with the government.

**Participant:** The hunters work with the logging companies. That's the logic.

**Participant:** All the logging, all the woods are coming to Europe.

**Participant:** Yes, I heard.

**Participant:** All the wood are coming to Europe. They have cut everything and the animals will run away.

**Participant:** Who is logging them?

**Participant:** We don't know. [crosstalk]

[laughter]

**Participant:** The pressure is also coming from the Europeans.

[crosstalk]

**Participant:** Do you know what? What is the name?

[crosstalk]

**Participant:** They are all being led by the Ghananians themselves.

**Participant:** What is their name? I went to Pagga early this year. I was walking around and I heard the taxi guy say, ''Do you know what,we had this donkeys. They were using them for their jobs."

**Participant:** Travelling purpose.

**Participant:** Then the Chinese came there,they are killing the aerials beacuse,there is a certain part of the ariels they use to make their women beautiful in China.??????

**Participant:** That is what I am asking. Doc is unable to tell me.

[crosstalk]

**Participant:** Why are we eating refurbrished meat [crosstalk] ???????

**Participant:** Of course it is nice,it is appetizer. [crosstalk]

**Participant:** You are forgetting something.

**Participant:** What?

**Participant:** The nutritional part of it.

**Moderator:** This is what I am saying, it is more nutritious.

**Participant:** We need to tackle that.

**Moderator:** You are right, I would prefer to eat bush meat,killed by somebody I know than to buy it along the road. I have got a lot of experience. I've bought a acrante and for two or three days I was running. I nearly died. Probably,they use poison. I know, there is a woman who buys the residue from the hunter. It is the best acrante and they make kebab . Next time you come just tell me. I will order [crosstalk]

**Moderator:** From which age please did you start eating bush meat?

**Participant:** I think, at a very very young age.

**Participant:** You didn't have any problem?

**Moderator:** It is because my father is a hunter.

**Participant:** His father was going to hunt them.

**Participant:** The father hunts animals which were cresont what is cresont in Dutch? Cultic. Please let me because I want to clarify what you are saying. All the bushmeat that we are buying like achem eginem and all those places. If I reach there, the animal attracts me. So I drop my car and buy it irrespective of whether it was being killed with DDT. You know DDT? Whether it is being killed or this or that. Before anything remove the intestines, the rest is what they're selling. So no idea of whoever went to kill,whether the hunter who killed animals which are healthy. No it is the feeling.

**Moderator:** That is for you.

**Participant:** That is old times.

**Participant:** No,till today my sister they are standing there. That is why I say,for the research,please when you reach Anthem just go there and see how people are buying. Whether they even ask the question of, ''How was this animal dead?'' or ''How did you kill?''

**Moderator:** Experience it.

**Participant:** They don't ask.

**Participant:** So you agree with what I am saying.

**Moderator:** No. They don't ask but my personal experience. I am talking about my personal experience.

**Participant:** Okay I know. When we are putting things in general terms,let us put it in general terms. If you make it personal it will be like,whoever buys bushmeat has this and--Even when we go to the restaurant-

**Moderator:** I am not saying that---

**Participant:** --we watch the soup and it attracts us and we are happy to see the soup you want to eat in the pan. So you don't go to wrestle them off. [crosstalk] Don't forget,I have made a trap to catch this animal before. [crosstalk]

**Participant:** Its because you have experience.

**Participant:** You have also done it before.

**Moderator:** I have made traps in the sky to catch,what do they call it?

[crosstalk]

**Participant:** So we are talking of experiences. A sick animal can be,an animal can be sick like we can also be sick. If the animal **[unintelligible 00:48:10]** the drugs which will heal it, that animal will be walking alone,come into my trap and when I trap that animal,I will use it for food. If I run,that does not mean somebody has put a poison.

**Participant:** Bushmeat.

[laughter]

**Participant:** Not until I had the money to buy along the road,I never bought along the road. Every meat that I ate was either coming from the neighbourhood at my aunts or it come from a bush,that's all. So I drove along Cape Coast to Accra and I bought one. We went and prepared it. Since then I said, "Look here" [crosstalk]

**Participant:** I did say that you see they will remove all the intestines and put [crosstalk]

**Moderator:** When you say fresh what do you mean? because probably we are [crosstalk] I bought the dried one.

**Participant:** Do you know what, we are talking about the grassland on the coast. That is why we call them grass cutters, that is their food.**[unintelligible 00:49:42]** .What we know these days is that some people use poison.

**Participant:** Yes, they do.

**Participant:** It is commercializing these meats that we cherish. People want money instead of doing the traditional way, they kill them, they gun and they sell. Now, even the fish.

You put a DDT they will come in front.

**Participant:** they will come in front and you see the mindset in my country now I think it’s very dangerous we have become egoistic, we're becoming materialistic, everybody want money.

**Moderator:**That's a little bit everywhere.

**Participant:** Everybody want to buy car everybody want to build a house so those who are in the traditional doing the farming instead of doing the right thing they use poison to kill these animals.

**Participant:**  There's a new trend.

**Moderator:**That's what I heard so now I’ve learned many things because I had not learned about the bats I had not heard about gorillas.

**Participant:** The dogs you've got it?

**Moderator:**The what?

**Participant:** Dogs.

**Moderator:**Dogs I already had the literature but we didn’t talk about it, but I know I lived in Singapore next to Malaysia and I think we laughed about that one.

[laughter]

**Participant:** What about the donkey.

**Moderator:**We've heard about the donkey and I think but--

**Participant:** **[unintelligible 00:51:03]**

**Moderator:**I think that back to your point really quickly just so that I can close it out in the last three groups the way that I organized the questionnaires it looks like people are saying the range per kilo for bushmeat is sometimes up to 200% more expensive here than in Ghana. Up to 200%

**Participant:** 100%

**Moderator:**Everybody agrees?

**Participant:** We all agree.

**Participant:** I don’t think when you buy the bushmeat here--

**Participant:** That's why **[unintelligible 00:51:36]** is saying that there is a new trend. Ghanaians now want an international market just like we have the poultry farmers and all that, of course, we also do poultry farm but it's not the right scale.

**Moderator:**Do what there?

**Participant:** Poultry and farming.

**Moderator:**Poultry yes.

**Participant:** Not in the large-scale like the way Europeans. We come down to what we were discussing about the bushmeat and that of grass cutter. He has an experience where he says he's not going to answer he knows the source in which it's coming from. A lot of farmers we have a lot of farmers who are really in this grassland now--

**Participant:** The place is different.

**Participant:** I like that one.

[laughter] [crosstalk]

**Participant:** The tilapia as well.

**Participant:** Why because you want to penetrate in the international market otherwise our children will never have a taste of it and they will always disagree with us until we leave.

**Moderator:**Why is it so expensive if it was cheaper you could buy it more often and the children--

**Participant:** Because it is not here it will be expensive.

**Participant:** No, it's also-- [crosstalk]

**Moderator:**Why 200% in your opinion?

**Participant:** It's imported.

**Participant:** No, not only importing we smuggled it in, that is why.

**Moderator:**Yes, that's what they told me already but just like I take-- [crosstalk]

**Participant:** No, no really we smuggled it.

**Participant:** Let's listen to Eliza

**Moderator:**I take stuff into America that I’m not allowed to take from France all the time and it's easy, easier than I think I can totally understand that but at least you last group at least you know where it is coming from but when you buy it here you don’t know where it's coming from is what they said. Is that what they said?

**Participant:** Yes.

**Moderator:**That’s a difference and people are comfortable with that? Maybe they prefer to bring it themselves because then it’s something that they know where it came from is that what you agree with?

**Participant:** Exactly.

**Moderator:**200% though is a lot. That’s a lot of money.

**Participant:** That's why we also use our filling to buy not everybody virtualizes the price.

**Participant:** I think that the price indication is a wrong indication. If you go to Ghana it's cheap, I can spend 150 Ghana cedis a week 100, 150 Ghana cedis which is about £40. Here I spend over 100 so that comparison I was thinking about it is not all that correct because you have to take the economy of the two places to compare it once and then probably look at the real cost there in relation to the economy and all that.

**Participant:** It depends on how easy you can get.

**Moderator:**Yes, it makes perfect sense to me because the two gentlemen to the left of Quasi, I can’t remember their names right now because everybody is pretty much anonymous anyways so I don’t have names it's just participant one, two, three but they said that the taste was different and that's why I thought, "Oh, maybe the 200% comes from the transportation cost because it's frozen and then de-thawed and then--" but then we were talking about if it was smoked then it retains its flavor to your point because you are the chef.

**Participant:** Saying that when we house them like back here we have pens for and we feed them, the taste is different from the one where you get it from the forest. It's the same as the chicken that we buy them here as compared to the free range so we can’t do anything about it. When you want everybody to taste it here we have to house them.

Now, free range then you have to house then you have to use other stuff to get them and that is where sometimes if you are not lucky you're going to run you don’t want that to happen and that is why Europeans of course when you are bringing them you only be happy when they don’t stop you from the security otherwise they'll be sick and then maybe-- [crosstalk] Only?

**Participant:** Only small fish.

**Participant:**  It's not true--

**Moderator:** They took your--

**Participant:**  They took my own, I came last August.

**Participant:** The smoke fish?

**Participant:**  Not the smoke fish but you know the-- [crosstalk]

**Participant:** They don’t know where.

[laughter]

**Participant:** They didn’t tell me really, but I asked me do you know that you are not allowed to bring this stuff. I told them I knew, and I told them that I’ve been eating this since my childhood and I am now 55 so look at me.

**Moderator:**Do they check every time? They're always--

**Participant:** It's randomly.

**Moderator:**Random.

**Participant:** It's not the first time.

**Participant:** They took some from me before.

**Participant:** That one was grasscutter. I got it and I freezed it. I thought six hours the freezing will still stay so when I came out and is saw this securities [foreign language] he was going through these things.

**Moderator:**He went through everybody’s things?

**Participant:** No, at a random.

**Moderator:**I thought you meant on random days, but they're random passengers.

**Participant:** No, they can call you.

**Participant:** Two three they pass and they--

**Participant:** Even being in France, they can call you or they fail. No, my mind tells me this lady can bring anything.

**Participant:** I think they saw it before they call you.

**Participant:** It is before the budget comes up.

**Participant:** Yes, before.

**Participant:** They target. They target but you don’t know.

**Participant:** It's true.

**Moderator:**Why would they scan it?

**Participant:** That's how they do it. It's a process.

**Participant:** The hand luggage? [crosstalk]

**Participant:** My bag before they put it on the stroller it’s already where they put it it's a small scanner it is bigger.

**Moderator:**Do they do it to like every day?

**Participant:** Every day you too they-- [crosstalk]

**Participant:** Holland they have it--

[background conversation]

**Moderator:**In Ghana?

**Participant:** When you are bringing your bag they ask you, do you have anything edible in it? Okay, so when they go to the machine before they put in the plane, they have to scan everything.

**Participant:** Are you sure?

[background conversation]

**Participant:** Every information here is important.

**Moderator:**I don’t want to miss it because I’m surprised.

**Participant:** When you put a weapon in your bag. When you get in something different any bag going to the plane, they want to see what is in there because--

**Moderator:**How can they see the meat? I can understand a gun.

[background conversation]

**Participant:** They scan.

**Participant:** All the bags they scan.

**Moderator:**How can they see--

**Participant:** Let me tell you about the scanning **[unintelligible 00:59:31]** It’s a new machine. Before they put the-

**Participant:** They scan it.

**Participant:** -straight away they have a big box that they put the things in and when they remove them from the plane when the plane arrives. Freighter the first thing they do before even we come out, they put everything on belt and putting it on belt the thing goes one after the other so there are people who are checking, it’s like a big hall.

**Participant:** They see it on the screen.

**Participant:** No, my one bag is being checked by two people so under no circumstance you will tell me we two didn’t see bushmeat in your bag. They will clarify so they will call. We have seen this bag this and that check it once again you will become a suspect, but it is in a fast beat so you never know.

[background conversation]

**Participant:** I have worked.

**Participant:** Same thing I went to Ghana and I worked for my car. I put in my lugguage so the time I went to Ghana they called me they put a sticker I didn’t know that they put sticker. They put sticker you know sticker?

**Participant:** Yes.

**Participant:** They called me and said what is in your bag? I said there is nothing they said, "No, you have something." I tell her, "Only air conditioning **[unintelligible 01:01:00]** They asked me, "So you don’t have nothing?" I say, "No." The air condition only one motor not true they told me to make a declaration.

**Participant:** Declaration.

**Participant:** I say no, I don’t pay nothing because it’s only one and my car I have already.

**Participant:** Removed it from airport.

**Participant:** I pay everything, so the motor is spoiled that's why I bought a new one.

**Moderator:**That's metal.

**Participant:** I've made a research also at the airport. I went the detail with the security. I know everything which comes there is being looked. Whether it's a suspicious metal--

**Participant:** Bushmeat.

**Participant:** Anything.

[laughter]

**Moderator:**I can imagine bushmeat?

**Participant:** In the Netherlands there is a time that we brought a machine. A machine which will look into your body and look whether because when there was a drug it is there but not specialty.

**Participant:** Not in Holland. In **[unintelligible 01:02:15]**

[background conversation]

**Participant:** They put me because I’m green.

[background conversation]

**Participant:** I'm not saying who are there watching--

**Participant:** I will tell you may experience of bushmeat. When you are carrying hand luggage, when you put it on the belt it goes through and they see that it's scanned. Equal when they go through your baggage they go through the same scan and they see a skeleton in there.

**Moderator:**What if it's not a skeleton what if it's just pieces of meat?

**Participant:** They see it.

**Participant:** They see.

**Participant:** You would not bring a whole in your bag.

[background conversation]

**Participant:** They have been doing it regularly, so they'll know.

**Participant:** Do you know what? I travel a lot. One time I was travelling from Amsterdam.

**Moderator:**This is amazing because a gun, I can understand if they are looking for guns, I can understand or a metal but if you're talking about just flesh--

**Participant:** With bones.

**Moderator:**Yes, what about if you don’t have bones?

**Participant:** I want to give you an experience. I was travelling from Amsterdam to London and I know Ghanaians who like dry fish, so I went to the market here I bought this fish **[unintelligible 01:04:01]** dry fish what they call it I bought a lot of and put it--

**Participant:** A lot of **[unintelligible 01:04:07]**

**Participant:** I have a lot of friends so I’m going to distribute so we put it on **[unintelligible 01:04:16]** I go to London Gatwick they called me. I told them, I said, "look I’m not coming from Africa. I’m coming from a European country, so it's allowed to buy **[unintelligible 01:04:32]** go through even my bag.

**Moderator:**It's intra not inter.

[background conversation]

**Participant:** I don’t know what they are doing.

**Participant:** Why do you think? I didn’t bring it from Africa my ticket says I’m coming from Amsterdam.

**Participant**I live in Amsterdam.

**Participant:** I live in Amsterdam.

**Participant:** I am a Dutch.

[laughter]

**Participant:** Why are you telling me about fish? All that I’m saying is that they do random checks. Me for the last time since I've been there.

**Participant:** Until they stop you and randomly search your bag. I've been stopped several times when I get out, Okay, this is my bag." but sometimes I forget my keys, the keys I forget them and that is where I have a problem but if you open your bag like all these drinks that we bring it’s there no big deal but the meat aspect that one they will because the law says don’t bring it in but they wouldn’t see it if your luggage-- [crosstalk]

**Participant:** Hold your meat can it go bad when I do I spread them in all the bags so when I come, my sister you know the lie that is what we do. You have to spread it. If they take this one they leave you with the other one. They ask you if you have more, "No, no it was only this one." and they are oblivious. Before you realize if you don’t take you suit to you bring it here next time you go you bring grasscutter.

**Participant:** I told you one country I believe in Australia. You don't go even for a visa.

**Moderator:**Yes, I hear I heard Australia yes, I did.

**Participant:** All your visa is done on the computer. They don't see your passport they don't do anything but the minute you land in that land there is the part that the dogs will be coming for you.

**Moderator:**No, Australia does not play. Australia is the real deal. They scare me because they are so organized, they're-

**Participant:** Super organized.

**Moderator:**-super organized and they have an army of dogs and--

**Participant:** Army of dogs to follow you?

**Participant:** With your bag then they where you go the dogs will follow you. When the dogs say, bye bye then you can go.

[laughter]

[background conversation]

**Moderator:**Did you watch on TV? Me too, I saw it on TV. Yes.

**Participant:** Very scary.

**Participant:** You were asking about the kids. Why are the kids not eating too much bush meat so if you are okay with that I don’t know if I have got it wrong

**Moderator:**Because we talked about a lot of the reasons why maybe kids aren’t eating it regularly because in Ghana, they are eating it every day and here they can’t eat it every day it's too expensive that's why we were talking about why is it expensive and how expensive it is and then we talk about other things.

**Participant:** They will never eat it even if they see it, they never eat it.

**Participant:** They take them from school.

**Participant:** Yes, because it's bad.

[background conversation]

**Moderator:**Yes so, many different choices whereas back in Ghana. Okay.

**Participant:** I say every 40 day I slaughter fresh chicken. When my children see that I am slaughtering they don’t eat it [foreign language] because I have killed an animal.

**Moderator:**What you have said makes sense to me because this is all people we are very far removed from nature. This is only one example there are many examples of how far removed we are from nature and I think that when we go and we see packages like this already just you go home and you cook it in a frying pan it doesn’t look like anything it doesn’t have a head it doesn’t have feet but then you eat it. Then I think that my children just your children will feel probably different.

**Participant:** Have you killed a cow before?

**Moderator:**I have not.

**Participant:** Killing a cow with a gun in the cow head. Chain it. system will come. When you go to the slaughterhouse--

**Participant:** No, it's not how they kill them.

[crosstalk]

**Moderator:** Maybe they maybe--

**Male Participant:**  No, no.

[overlapping conversations]

**Male Participant:** What I know is that they've got the how.

**Moderator:** Okay.

**Male Participant:**  We've got the Jewish people. We've got the Nubians.

**Male Participant:** The Muslims also, that is the halal.

**Male Participant:**  You see, they-- What is it that you cut it--

**Male Participant:** The blood.

**Moderator:** And you drain the blood?

**Male Participant:**  Yes, it's same with the Jewish but it's only the scientific Europeans, they do the killing- **[unintelligible 01:10:42].**

**Male Participant:** All of you guys, it's my profession I work in this country with them, the halal and all those mind different because at the end of the day it is strained out, the neck is cut, the blood is oozing--

**Male Participant:**  Don't forget I believe.

**Male Participant:** I understand, you should be able to cut all the other--

[crosstalk]

**Male Participant:** What happens to the other parts **[unintelligible 01:11:11]**

**Male Participant:** They remove, it's all mechanized, the head, what do you call it? The head is, goes another line.

**Male Participant:** What happens to that? The skin.

**Male Participant:** The skin it goes to the other one, the intestines go to the other one and we go and inspect. Normally they have to be inspected, when you inspect you see that there are signs of diseases, it goes to another line.

**Male Participant:**  Yes, it's true.

**Male Participant:** We'll pick only those which are--

[crosstalk]

**Male Participant:** How many factors have you got for the reason why here the bushmeat is not being patronized. You have only got the financial aspect of it.

**Moderator:** Why it's not being patronized? Many indicators at home--

**Male Participant:** What have you-- I want to know from you.

**Moderator:** Yes, okay Suna. I have to get all the information first and then I have to organize it myself because I have three groups and a pilot which I didn't record.

**Male Participant:** I want to know why do you think that the price is very exorbitant?

**Moderator:** I'll tell you what I think. Why bushmeat has not been patronized here and if that's the word that you choose. That wouldn't be my word but that's your word. The study coming out of the United States and the Liberian community, they did an extensive study like this but they got much bigger-- The reason why they did it out of Minnesota is because there was ebola explosion in the media in America and Liberian children were being sent home from school if they had a temperature and then the school would say, "You have Ebola. We think you have Ebola, go home." It was a very bad situation for the Liberian community.

The media was a big problem and it caused that community to be stigmatized. You can then not talk about bushmeat and be a conversation that people understand at that point. This team came and they did a focus group and they had the results published and it changed the whole-- It demystified and I think the very first pilot we had, do you remember what I said? I said, "If you don't tell people the truth, and they don't know the truth from the media, they will make up their own story."

So, you have a choice, you can either not tell your story unless somebody else do it for you, or you can say, "If you want to know, this is the truth. This is my truth," and have it be like that. I do think that the media has a big just ignorance in general in the media. If you take ignorance and the media together, that's dangerous because if you're ignorant you can't challenge, you don't have the critical thinking skills to say, "That doesn't sound right, or maybe I should just do my own research on the topic." You just let people tell you what they think and it's journalists.

**Male Participant:** You become a consumer terrorist.

**Moderator:** Journalists are not academics, journalists will write about bushmeat in one way, they are journalists. They want their name--

**Male Participant:** Without any research being conducted.

**Moderator:** In academics it's different, academics, your name can only be recognized if you do thorough research that's scientific and that is unbiased because if it's biased it's not valid.

**Male Participant:** I don't argue because generally, we've got levels, we've got scientific journalists in the medical world, they do analytical work.

**Moderator:** They do.

**Male Participant:** There are also generalized, the **[unintelligible 01:15:27]** and then there's a superficial aspect of society-- You have to differentiate.

**Moderator:** Certainly. When you're talking about Ebola, I would say that it sounds like to me that it's more the journalists that are-- Maybe not the scientific journalists.

**Male Participant:** I do have other questions.

**Moderator:** Yes, I do. I want to make sure that you--

**Moderator:** Because we've talked about some of the other things in the past.

**Male Participant:** That is why I draw you back to the your paper, what is on the paper.

**Moderator:** Culturally, I think that locally it's hard to educate people on why there's the connection but the fact that it's organic meat, and the health, these are very first pilots, you remember you said, "Why don't you study the meat?" You told me. "Why don't you study the meat and tell me what's healthy and what's not healthy about the meat?" I think my final question today would be, in your opinion what is the best way to kill germs and diseases in the meat? If there are diseases present, just like any meat.

We talked about domestic meat, and being too clean, and then more bacteria comes perhaps. What about diseases that actually do exist in the forest, deep in the forest? Whatever they are, how do you kill those germs and those pathogens in your opinion?

**Male Participant:** Let me start. In the first place by talking about, that is where I went to and arguing with the doc. In the first place, number one, it's your belief, if I'm going to kill an animal to eat, it is my belief because I believe this animal has got certain nutrients that will help me. All this animal if I prepare it with soup versus how to get the animal as-- From my generational thinking, this animal possesses certain things which is good for human nutrition.

With that in my mind, I will just kill the animal and we have our own traditional way of cleansing meat. By applying that traditional way, I know I'm 100% convinced, whether the animal has got measles from head to toe, any bacteria or those things. From my generational thinking, we have been eating by preserving our meat through this means, and there's nothing coming out of it except exceptional cases.

Who am I to let's say, go and buy meat from Cape Coast to Accra and then eat, after eating it my mindset will tell me, "That meat was the meat which was meant to be runny." Runny is a disease which can occur every time anywhere. After you have taken anything, you may drink any water in Cape Coast and when you come back to Accra to eat something else, the water that you drink can start you the runny. In your mindset, you'll think, "The meat I ate, no no I'm not in that."[crosstalk]

**Moderator:** What I mean is it--

**Male Participant:** Alive.

**Male Participant:** When we eat generational and the ladies will also bear with me, when we eat by this rules too--

**Male Participant:** To tell your story.

**Male Participant:** Yes. When we buy this bushmeat, we have ginger, we have other things, pepper, an average of ways. We stir it, we cook it thoroughly to kill all bacterias, that is a belief. That is why I started with belief and after doing that, you know it's being preserved very good to eat, it's tasty.

**Moderator:** Okay, so boiling.

**Male Participant:** Boiling, but not really boiling but--

**Moderator:** In the questionnaires I have, how many do I have? Like 15 I think. In the questionnaires, I have a question you'll have it, and many respondents say it's not boiling, it's smoking.

**Moderator:** That kills but--

**Participant:** Both, but after smoking it you put in the pan, chop it in pieces, put in a pan and then put your sauces around before you make your soup, whatever you want to make.

**Moderator:** You put the smoked in the soup?

**Participant:** After smoking. The reason for the smoking is for preservation because we didn't have fridges and all those things.

**Moderator:** Yes, we talked about that. I don't know that you put the smoked in the soup, I thought-- I need to go back to the question because it said smoking or boiling but actually, it's smoking and then boiling.

**Participant:** She is talking about roasting, we have it also. Sometimes the--

**Participant:** When they've got to do some wine, they roast it and they eat.

**Participant:** It's three issues, what you were discussing and what you are telling me now is three issues. Animals when we kill them fresh, we don't have deep freezers, fridges, and all those things. We smoke them to be able to stay for long period.

**Moderator:** Then you put it in the soup?

**Participant:** Before we prepare it into the soup, we don't put the whole, let's say the whole meat, the whole **[unintelligible 01:21:24]** into one soup, we divide them and we put which one passes or which one you want to use for the meal.

**Moderator:** Some people like to dry it all the way through and some people like it pink inside, a little bit pink inside.

**Participant:** Yes, because of the embalming, before you embalm the body you need to put some **[unintelligible 01:21:53]**

**Participant:** He's not giving another one, embalming the meat.

**Participant:** Yes, we do it also.

**Moderator:** Embalming.

**Participant:** You never see it.

**Participant:** How do you embalm the meat?

**Participant:** Tell them in Cape Coast, we put them in certain water sauce.

**Participant:** I only know smoke, why you smoke it.

**Participant:** We put on the special water before--

**Participant:** No, for embalming.

**Participant:** We have special spaces, they preserve it a special way.

**Participant:** We are talking about bushmeat.

**Participant:** No, we are talking about bushmeat. Bushmeat also we have a certain bushmeat before you dry it, you put them in a sauce before you dry it to get a good taste.

**Participant:** Which sauce?

**Participant:** Like the pink one she's talking about, it's not pink--

**Moderator:** No, I was talking about-- I meant some people in the group, they don't like it too dry, so it's still a little bit moisture inside.

**Participant:** What I know, especially what we know, I have stayed in the North for a long time, the meat they sold them and they dried them. A lot of salt and they dry them and you can keep it for years.

**Participant:** It's embalming with salt?

[crosstalk]

**Participant:** It took them far away from the North, they walked to this coast and boarded to America, some died, some stayed. Those who didn't die were the strong because they were eating good food.

**Moderator:** Yes, nutrition.

[crosstalk]

**Participant:** Like what you are saying, some people wanted 50/50 like the pink and-- it depends on how you want it.

**Participant:** The question goes, how do we make sure that no bacteria, that's the question.

**Participant:** It comes to my point of view that because of the way we use the sauces and the, how do we say clouding?

**Moderator:** Vegetable herbs.

**Participant:** Herbs and spices, how we preserve it with that.

**Participant:** No, it's not in the kitchen. How do you make sure that there's no bacteria?

**Participant:** I'm **[unintelligible 01:25:03]** .

**Participant:** No, let the woman talk.

**Participant:** Maybe later.

**Participant:** How do you make sure that bacteria is not in our food?

**Participant:** With bushmeat, you have to, when you have it you receive it from Ghana, you have to put it in the oven to be more dry so that there's not too much water in it, not too dry, not too moist, so that you can keep it longer.

**Moderator:** In the oven?

**Participant:** Yes. I would smoke it in Ghana and you can also put it in the oven or you put it in the freezer. You do it into small pieces so that anytime you need it, you just take it and you use it.

**Moderator:** Yes, because that's the other thing that they said is that you don't buy bushmeat by the kilo because it's so expensive, you buy it by the piece.

**Participant:** It's true. I think temperature cause bacteria, isn't it?

**Moderator:** Pardon?

**Participant:** Temperature.

**Participant:** When it's moist.

**Moderator:** Yes.

**Participant:** About 100 degrees, you kill any bacteria except the stubborn ones, maybe the Ebolas but I know when I cook a lot, so when you come from Africa, you put it in the oven and make sure it's above 100 degrees. The taste is still there, fresh meat, everything before I use it to cook. What I've realized is that I cook for white people, I use cold beers all this time but I've noticed there's such a way, I think you use liquor. I do this as I prepare it. After eating it I say, "Don't worry you've eaten some **[unintelligible 01:26:54]**

**Moderator:** They didn't know?

**Participant:** Yes.

**Moderator:** That's what my mom did to me when I was a child. She made me eat a horse meat but I used to ride horses.

**Participant: [unintelligible 01:27:10]** but me, my main problem with bushmeat which you don't know the source is that sometimes-- When you see these friends more and more--

[background conversation]

**Participant:** What he is saying is anytime like what I was talking about, the trap that I was using to catch this bush animals. Sometimes, I go there once in a week in the village to check my trap if it has gotten any animal. Sometime I'll get a grass cuter, I'll get **[unintelligible 01:27:49]** and those things, different animals. Sometimes it is half decomposed but that decay side is the sweetest part, one because it gets sometime special scent.

What I do is, I put it on a fire, he was talking of this worms. When you put them on fire, the worms they die, they are burned. Automatically there are some who stay but those ones which will stay for the benefit of any doubt, that is why we use these spices to spice the food, to make it 100% for the mindset. I don't know what he thinks, and that is why.

**Participant:** The spice is not anti-bacterial.

**Participant:** Talking about the bacteria--

**Participant:** They are cooking.

**Participant:** Not only the cooking, some bacterias can stay more than 100 degrees. Some bacterias can stay. Most of the bacterias are **[unintelligible 01:28:54]** Bacterias are, there are research going on to search for-- **[unintelligible 01:29:01]** In order to be precise, just preserve it with what our forefathers were doing, and add the ginger and pepper. Eventually, if you want extra pepper and all those, have the, because when those things go into our body, it kills other bacterias and it makes us fit. The decay of the bushmeat occurs, that is how we cure it and we don't have no problem.

**Participant:** Look at the train **[unintelligible 01:29:37]** those days at the train--

**Participant:** The railway line.

**Participant:** There were special weeks granted along, that one when you have to get this fresh meat and so forth, you cook with them.

**Participant:** Alan knows a lot.

**Participant:** You put them, no ginger they believe that the prices might **[unintelligible 01:30:02].**

**Participant:** Reduce the bacterial level of--

**Participant:** Do not believe it because of the data. Conspiracy theories.

[laughter]

**Participant:** I didn't believe that [crosstalk].

**Participant:** I was.

**Participant:** [crosstalk] whether Ghana or Cameroon has this crisis. They don't eat their [crosstalk].

**Participant:** I'm going to add that.

**Participant:** For the bacterial, they eat too much spice.

**Participant:** Too much spice, yes. It's too much.

**Participant:** He wants us to push it? He still have poor **[unintelligible 01:30:40]** and he **[unintelligible 01:30:40].**

**Participant:** Yes. Thank you. That I don't think I've ever seen it. It's important because--

**Participant:** When you go to Ghana you can't do the same thing.

**Participant:** Yes, I am.

**Participant:** [foreign language]

[laughter]

[crosstalk]

**Participant:** They already told us that **[inaudible 01:31:16].** I'm going to give the survey like I do every week again. If you could, please. Those who have not done it before, you can. If you can answer all of the questions, then, I can-- It's a questionnaire. Don't put your name, just put female or male, that's all. Just because it's easier to know who's female and who's male. Thank you. Did you do one already?

**Participant:** No.

**Participant:** No, okay. Don't be late because then it's hard for me to compare between groups. Thank you. You already did it, I know.

**Participant:** I did it also.

**Participant:** I know. Thank you, and you also. I learned a lot from the questionnaire.

**Participant:**  Which means you are--

**[01:32:46] [END OF AUDIO]**
