# Supplementary material for: Exploring the characteristics of a local demand for African wild meat: A focus group study of long-term Ghanaian residents in the Netherlands
Source: PLoS One. 2021 Feb 16;16(2):e0246868. doi: 10.1371/journal.pone.0246868 (PMC7886224; doi:10.1371/journal.pone.0246868)
Supplement: S4 File — (DOCX) [file pone.0246868.s008.docx]

Tabulated Results - African Focus Group Data Compilation 2018-2019

|  | Rounded Percentage (%) | Calculations | Notes |
| --- | --- | --- | --- |
| Age |  |  |  |
| 18-29 | 4 |  |  |
| 30-50 | 18 |  |  |
| 51-65 | 61 |  |  |
| 65-80 | 18 |  |  |
| Total |  |  |  |
| Participant |  |  |  |
| Ghanaian |  |  |  |
| Gender |  |  |  |
| Female | 46 |  |  |
| Male | 54 |  |  |
| Total |  |  |  |
| How long have you lived in the Netherlands? | | | |
| < 1 year | 7 |  |  |
| 5-10 years | 11 |  |  |
| 11-25 years | 18 |  |  |
| 26-35 years | 50 |  |  |
| 36-50 years | 14 |  |  |
| Total |  |  |  |
| How often do you travel to your country of origin? | | | |
| Less than once a year | 33 |  |  |
| 1-2 times a year | 52 |  |  |
| More than 2 times a year | 15 |  |  |
| Total |  |  |  |
| Name of favorite food/recipe? | | | |
| Grasscutter/Light Soup | 25 |  |  |
| Fufu with Bushmeat | 14 | 46% | Denominator adjusted to remove non-answers |
| Palmnut Soup | 7 |  |  |
| Fufu/Hwee Nkee | 14 |  |  |
| Fish | 4 |  |  |
| No meat | 18 |  |  |
| No answer | 14 |  |  |
| Fufu with domestic meat | 4 |  |  |
| Total |  |  |  |
| What is the best way to kill infectious diseases in African wild meat? | | | |
| Boiled | 40 |  | 1st |
| Smoked | 25 |  |  |
| Smoked and Boiled | 15 |  |  |
| Grilled | 5 |  |  |
| No answer | 15 |  |  |
| Total (Group 1 was not asked this question (28-8=0)) |  |  |  |
| How do you feel about substituting local domestic meat instead of using African meat for African recipes? | | | |
| Negative | 64 | 0.7 | Denominator adjusted to remove non-answers |
| Positive | 29 |  |  |
| No answer | 7 |  |  |
| Total |  |  |  |
| What if the wild meat comes from Europe? | | | |
| Positive | 8 |  |  |
| Negative | 92 |  |  |
| No answer | 0 |  |  |
| Total |  |  |  |
| Do you have any concerns regarding the health risks of meat originating from Africa? | | | |
| Yes | 36 |  |  |
| No | 54 | 0.6 | Denominator adjusted to remove non-answers |
| No answer | 11 |  |  |
| Total |  |  |  |
| What do you like about African wild meat? |  |  |  |
| Tradition and Culture, Health (B&E) | 4 |  |  |
| Taste (A) | 39 |  |  |
| Tradition and Culture (B) | 14 | 0.6 | Denominator adjusted to remove non-answers |
| Taste and Tradition and Culture (A,B) | 4 |  |  |
| Taste, Tradition and Culture and Community Building (A,B,C) | 4 |  |  |
| Community Building | 4 |  |  |
| Religion | 4 |  |  |
| Health Benefits | 7 |  |  |
| Taste and Health | 4 |  |  |
| Taste, Tradition and Culture plus Health | 7 |  |  |
| No answer | 11 |  |  |
| Total |  |  |  |
| What do you dislike about African wild meat? |  |  |  |
| Nothing | 25 | 0.333333333 | Denominator adjusted to remove non-answers |
| How animals are trapped/hunted | 18 |  |  |
| Preservation | 7 |  |  |
| Not knowing source of wild meat | 11 |  |  |
| Not knowing how animal was killed (i.e., poison) | 4 |  |  |
| Difficulty acquiring it | 4 |  |  |
| What the animal eats? | 4 |  |  |
| Not knowing about the quality of the meat (infected?) | 4 |  |  |
| No answer | 25 |  |  |
| Total |  |  |  |
| How many times a year do you consume bushmeat? | | | |
| No answer | 18 |  |  |
| Never | 11 |  |  |
| Less than once a year | 25 |  |  |
| 1-2 times a year | 21 | 0.565217391 | Denominator adjusted to remove non-answers |
| 3-5 times a year | 11 |  |  |
| 5-10 times a year | 0 |  |  |
| 10+ times a year | 14 |  |  |
| Total |  |  |  |
| Are there particular times of the year or special occasions when African wild meat is consumed? | | | |
| No | 57 | 0.941176471 | Denominator adjusted to remove non-answers |
| No answer | 39 |  |  |
| Traditional Gatherings | 4 |  |  |
| Total |  |  |  |
| How do you acquire it in the Netherlands? |  |  |  |
| No answer | 14 |  |  |
| Through friends | 54 | 0.625 | Denominator reflects the removal of non-answers |
| Mail/Courier | 0 |  |  |
| Local Butcher | 18 | 0.208333 | Denominator reflects the removal of non-answers |
| Local Restaurant | 0 |  |  |
| I do not buy bushmeat in the Netherlands | 7 |  |  |
| Bring it myself | 7 |  |  |
| Other | 0 |  |  |
| Total |  |  |  |
| Is African wild meat more expensive in the Netherlands? | | | |
| Yes | 82 |  |  |
| No | 4 |  |  |
| Not allowed | 4 |  |  |
| No idea | 7 |  |  |
| Total |  |  |  |

Drivers are shaded yellow. Health Considerations are shaded green. Willingness to Pay (WTP) factors are shaded blue. Local Demand is shaded orange. Hunting Methods are shaded turquoise.
